# Supplementary material for: Photocatalytic Multicomponent Annulation of Amide-Anchored 1,7-Diynes Enabled by Deconstruction of Bromotrichloromethane
Source: Molecules. 2024 Feb 8;29(4):782. doi: 10.3390/molecules29040782 (PMC10893216; doi:10.3390/molecules29040782)
Supplement: Supplementary file 1 [file molecules-29-00782-s001.zip › molecules-2820688-supplementary.pdf]

Supporting Information

Photocatalytic Multicomponent Annulation of Amide-Anchored 1,7-Diynes Enabled by Deconstruction of Bromotrichloromethane

Daixiang Chen [1], Yu Bao [1], Shenghu Yan [1], Jiayin Wang [1,*], Yue Zhang [1,*] and Guigen Li [2]

[1] School of Pharmacy, Changzhou University, Changzhou 213164, China
[2] Department of Chemistry and Biochemistry, Texas Tech University, Lubbock, TX 79409, USA; guigen.li@ttu.edu
* Correspondence: wjychem@cczu.edu.cn (J.W.); zyjs@cczu.edu.cn (Y.Z.)

General Information…………………………………………………………………….. S2

Preparation of Substrates **1**…………………………..……………………………..… S2

Luminescence Quenching Experiment………………………………………………… S2-S3

Control Experiment with $H_2{}^{18}O$…………………………………………………… S3

X-ray Crystallography Structure…………………………………………………… S3-S5

Deuterium-Labeled Experiments…………………………………………………….. S5

General Procedure for the Synthesis of Compound **3**…………………………………. S5-S6

Characterization Data of Compound **3**……………………………………………..… S6

General Procedure for the Synthesis of Compound **4**…………………………………. S6

Characterization Data of Compound **4**……………………………………………..… S6-S7

General Procedure for the Synthesis of Compounds **2**………………………………… S7

Characterization Data of Compounds **2**………………..…………………………… S7-S14

Copies of ^{1}H and ^{13}C NMR Spectra for Compounds **2**, **3** and **4**……………………..… S15-S64

General Information

^{1}H NMR (^{13}C NMR) spectra were measured on a Bruker DPX 400 MHz spectrometer in $CDCl_3$ (DMSO-d_6) with chemical shift (δ) given in ppm relative to TMS as internal standard [(s = singlet, d = doublet, t = triplet, brs = broad singlet, m = multiplet), coupling constant (Hz)]. HRMS (ESI) was determined by using microTOF-QII HRMS/MS instrument (BRUKER). The melting points were measured with digital melting point detector. PE refers to petroleum ether (bp 60-90 °C), and EA refers to ethyl acetate. Other reagents, unless otherwise noted, were purchased from commercial vendors and used without further purification. X-Ray crystallographic analysis was performed with a Siemens SMART CCD and a Siemens P4 diffractomete.

Preparation of Substrates 1

1,7-diynes are known compounds and were prepared according to literature procedures (*Org. Lett.* **2022**, *24*, 5126-5131; *Org. Lett.* **2018**, *20*, 6765-6768). The general procedure was described using substrate **1a** as example.

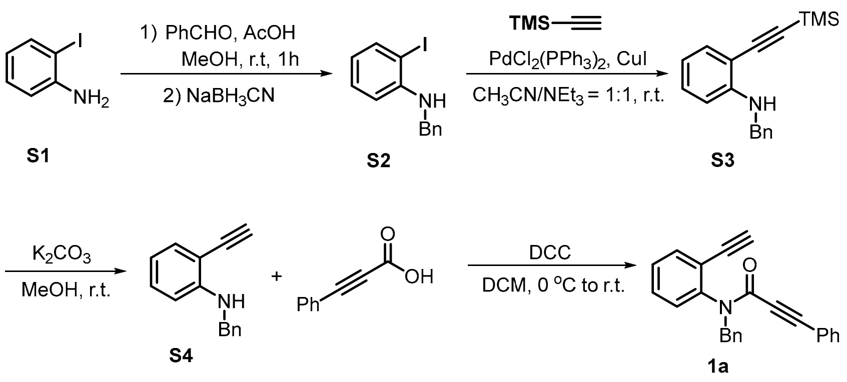

Luminescence Quenching Experiment

The luminescence quenching experiment was taken using a FS5 Spectrophotometer (Edinburgh FS5). The excitation wavelength was 387 nm. The emission intensity was collected at 529 nm. The samples were prepared by mixing *fac*-Ir(ppy)$_3$ (1.0× 10^{-4} mol/L) and different amount of quenchers (1,7-diyne **1a** and $BrCCl_3$) in EtOH (total volume = 2.0 mL) in a light path quartzfluorescence cuvette. The concentration of 1,7-diyne (**1a**) stock solution is 1.0× 10^{-4} mol/L in EtOH. The concentration of $BrCCl_3$ stock solution is 1.0× 10^{-4} mol/L in EtOH. For each quenching experiment, each volume of quenchers stock solution was titrated to a mixed solution of *fac*-Ir(ppy)$_3$ (0.2, 0.2, 0.2, 0.2, 0.2, 0.2, 0.2 μL, in a total volume = 2.0 mL, ignoring changes in volume). Then the emission intensity was collected and the results were presented in Figure S1 and Figure S2.

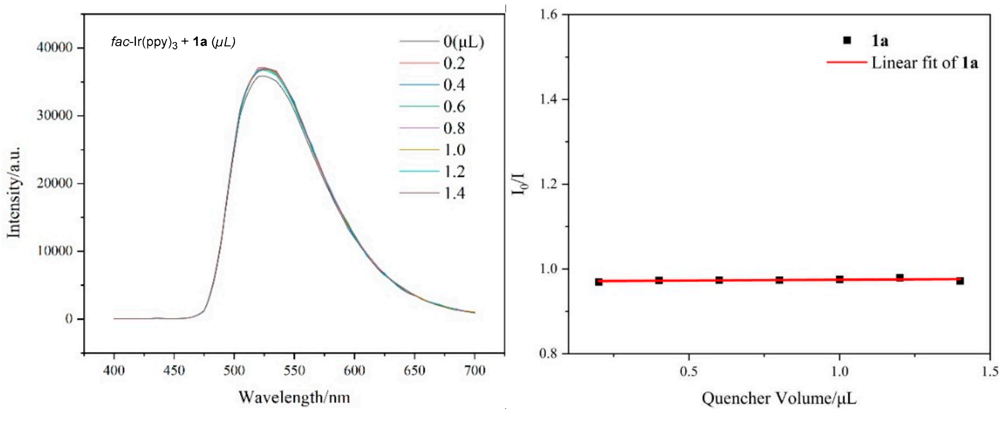

Figure S1. Stern-Volmer analysis for *fac*-Ir(ppy)$_3$ with **1a**

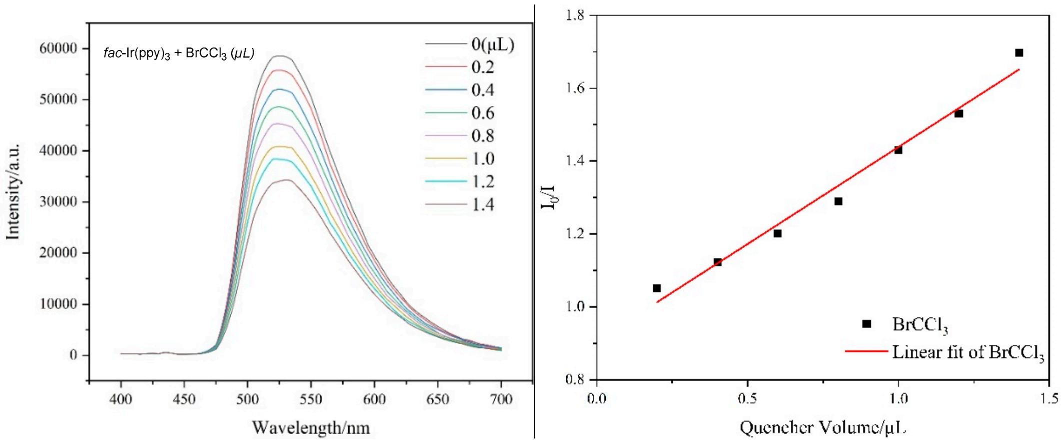

Figure S2. Stern-Volmer analysis for *fac*-Ir(ppy)$_3$ with $BrCCl_3$

Control Experiment with $H_2{}^{18}O$

In a 25-mL Schlenk tube, 1,7-diyne **1a** (0.1 mmol, 33.5 mg, 1.0 equiv), $BrCCl_3$ (0.2 mmol, 39.6 mg, 2.0 equiv), K_2CO_3 (0.2 mmol,27.6 mg, 2.0 equiv), *fac*-Ir(ppy)$_3$ (0.6 mg, 1 mol%) and EtOH/H_2O^{18} (1.0 mL, 100:1 V/V) were successively added under Ar conditions. Then, the tube was stirred at 60 °C for 36 h under 30 W light (blue LEDs) irradiation until complete consumption of **1a** as monitored by TLC analysis. After the reaction was completed, the product **2a** was detected by HR-MS. $^{16}O/^{18}O$ = 3.18:1.

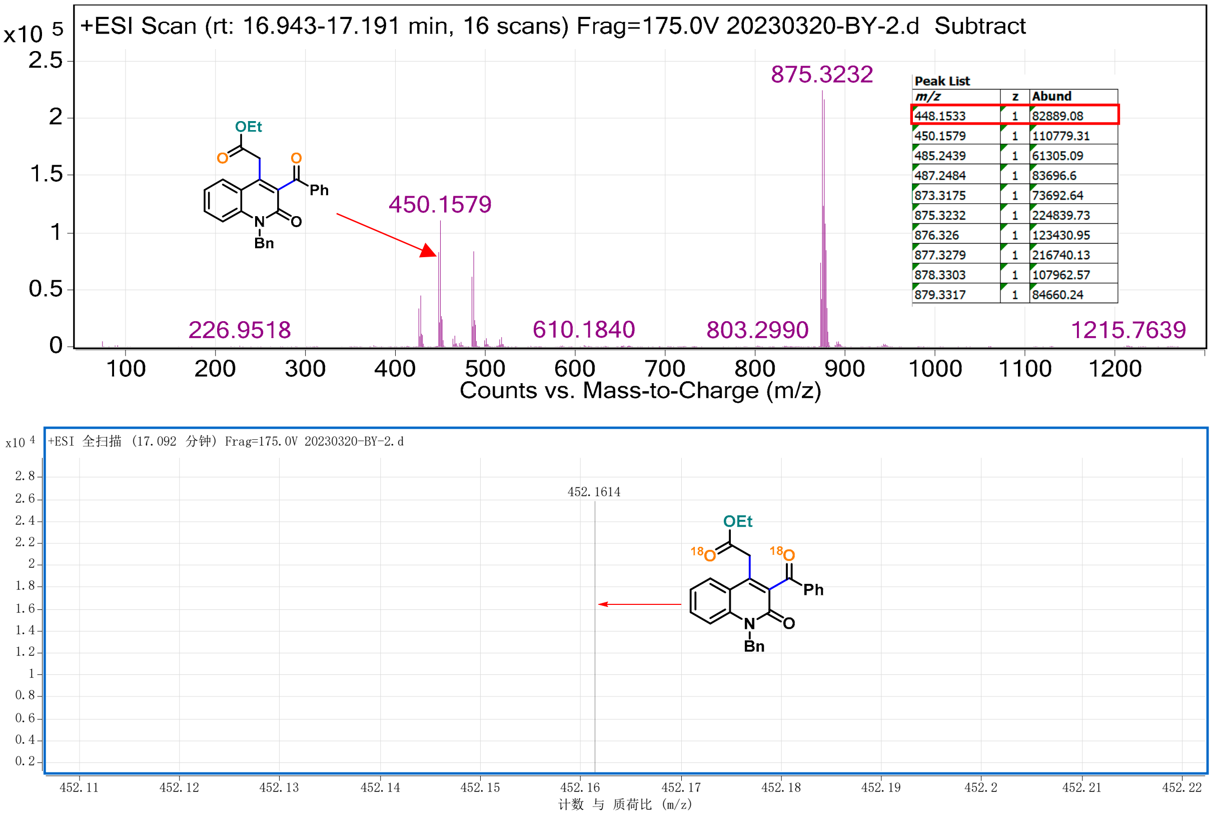

Figure S3. Copy of HR-MS Spectrum of Product 2a (^{16}O and ^{18}O)

X-ray Crystallography Structure

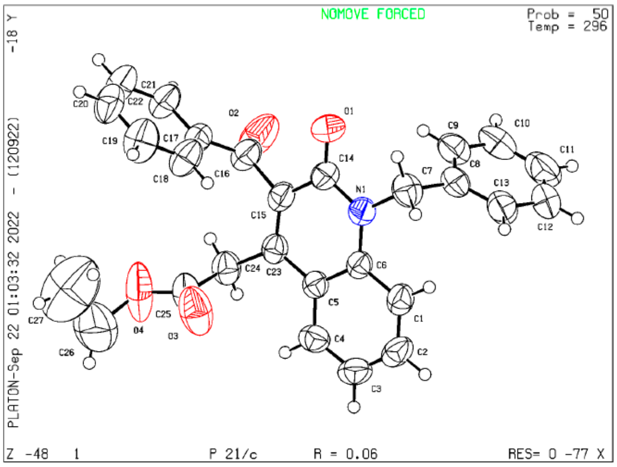

Figure S4. X-Ray Structure of Product **2a** (the ellipsoid contour 30% probability levels)
CCDC (2294859)

A single crystal **2a** was obtained by slowly evaporating in EtOH at room temperature under the air conditions.

Table S1 Crystal data and structure refinement for 2a

Identification code	1
Empirical formula	$C_{27}H_{22}NO_4$
Formula weight	424.45
Temperature/K	296.15
Crystal system	monoclinic
Space group	$P2_1/c$
a/Å	10.4286(10)
b/Å	17.3591(17)
c/Å	12.5754(13)
α/°	90
β/°	101.187(2)
γ/°	90
Volume/Å^3	2233.3(4)
Z	4
ρ_{calc}g/cm^3	1.262
μ/mm^{-1}	0.085
F(000)	892.0
Crystal size/mm^3	0.26 × 0.2 × 0.15

Radiation	MoKα (λ = 0.71073)
2Θ range for data collection/°	5.738 to 49.994
Index ranges	-12 ≤ h ≤ 10, -20 ≤ k ≤ 19, -7 ≤ l ≤ 14
Reflections collected	11228
Independent reflections	3919 [R_{int} = 0.0311, R_{sigma} = 0.0347]
Data/restraints/parameters	3919/1/290
Goodness-of-fit on F^2	1.071
Final R indexes [I>=2σ (I)]	R_1 = 0.0586, wR_2 = 0.1647
Final R indexes [all data]	R_1 = 0.0968, wR_2 = 0.1815
Largest diff. peak/hole / e Å^{-3}	0.35/-0.29

Deuterium-Labeled Experiments

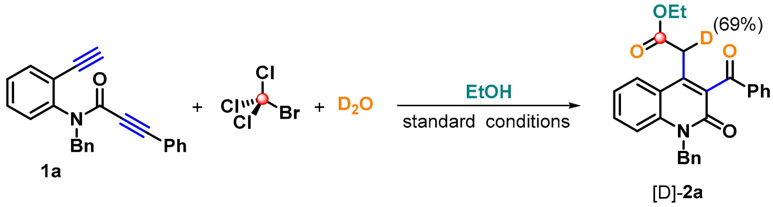

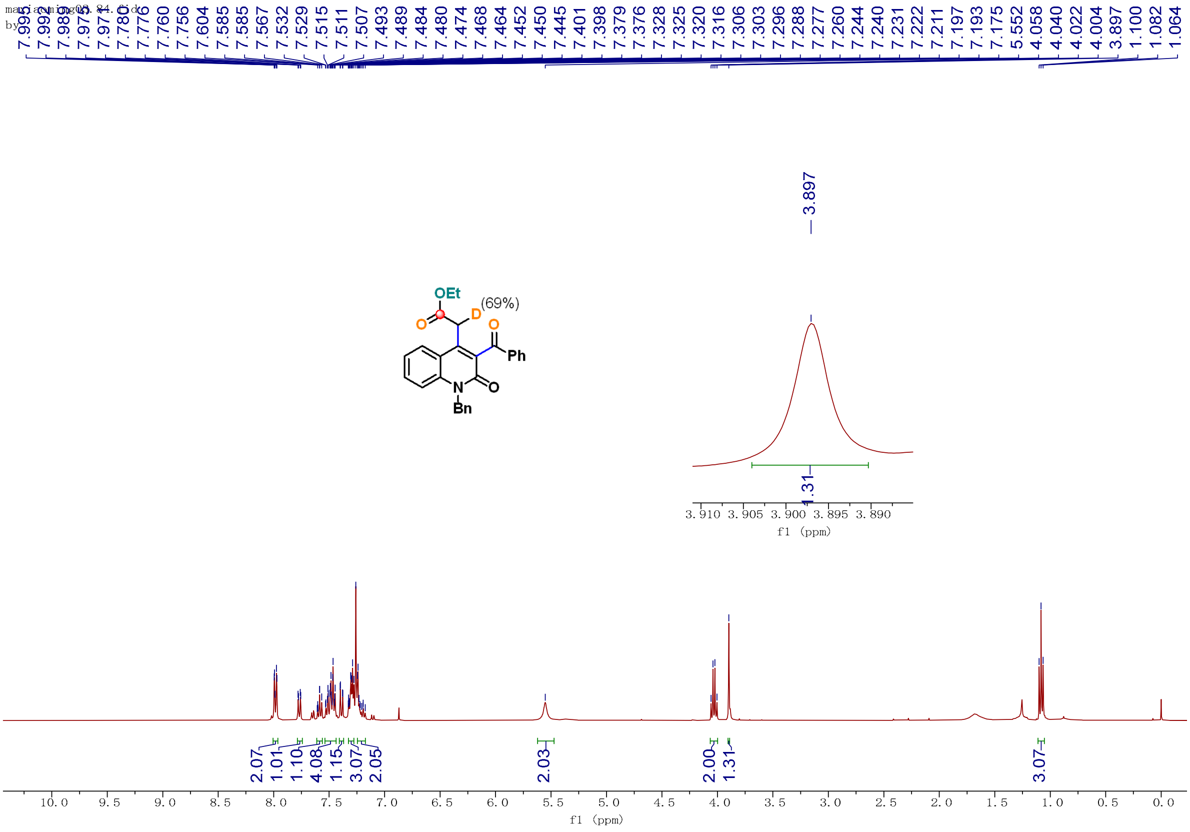

^{1}H NMR Spectrum of Compound [D]-2a

General procedure for the synthesis of compounds 3

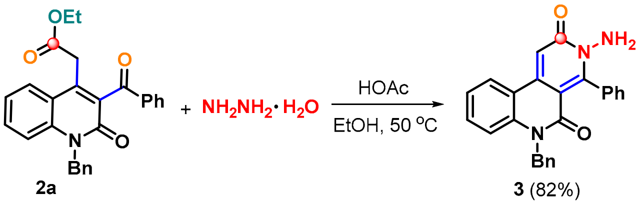

To compound **2a** (0.1 mmol, 42.5 mg) a solution of hydrazine hydrate (0.5 mL) and acetic acid (one drop) in 1.0 mL EtOH was added and the mixture was stirred at 50 °C for 2 hours until TLC analysis showed that **2a** was completely consumed. The reaction mixture was then diluted with water, extracted with ethyl acetate, washed with water until neutral, and concentrated the organic layer under reduced pressure. The residue was purified through preparative thin layer chromatography (petroleum ether/ethyl acetate = 1:1 v/v) to afford compound **3** as white solid.

3-amino-6-benzyl-4-phenylbenzo[c][2,7]naphthyridine-2,5(3H,6H)-dione (3)

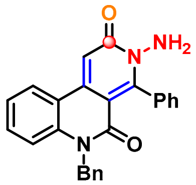

White solid; 32.2 mg, 82% yield; mp: 241-243 °C; ^{1}H NMR (400 MHz, DMSO-*d*$_6$) (δ, ppm): 8.40-8.38 (m, 1H), 7.50-7.42 (m, 5H), 7.38-7.33 (m, 2H), 7.30-7.17 (m, 5H), 7.14-7.09 (m, 2H), 6.04 (s, 2H), 5.27 (s, 2H). ^{13}C NMR (100 MHz, DMSO) (δ, ppm) 159.2, 159.0, 153.0, 141.6, 138.8, 137.3, 135.5, 132.4, 129.0, 128.5, 128.5, 127.9, 127.3, 126.7, 125.6, 123.0, 117.0, 116.4, 105.3, 104.4, 45.1, 39.3. HRMS (ESI) m/z calcd for $C_{25}H_{19}N_3O_2Na$ $[M+Na]^+$ 416.1375, found 416.1375.

General procedure for the synthesis of compounds 4

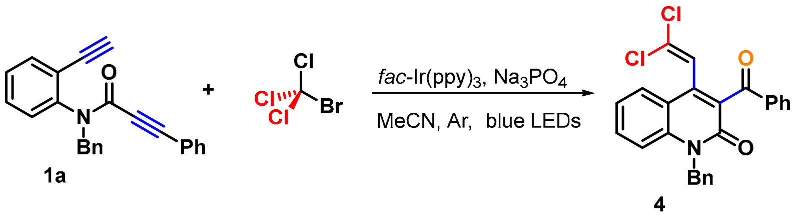

In a 10-mL Schlenk tube, 1,7-diyne **1a** (0.1 mmol, 33.5 mg, 1.0 equiv), $BrCCl_3$ (0.2 mmol, 39.6 mg, 2.0 equiv), Na_3PO_4 (0.2 mmol, 32.8 mg, 2.0 equiv), *fac*-Ir(ppy)$_3$ (0.6 mg, 1 mol%) and MeCN (1.0 mL) were successively added under Ar conditions. Then, the tube was stirred at room temperature for 12 h under 30 W blue light (blue LEDs) irradiation until complete consumption of **1a** as monitored by TLC analysis. After the reaction was completed, the reaction mixture was concentrated in vacuum and the resulting residue was purified by column chromatography on silica gel (eluent, petroleum ether/ethyl acetate = 15:1) to afford the desired product **4** (16.0 mg, 37%) as a white solid.

3-benzoyl-1-benzyl-4-(2,2-dichlorovinyl)quinolin-2(1H)-one (4)

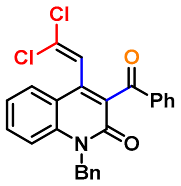

White solid; 16.0 mg, 37% yield; mp: 133-135 °C; ^{1}H NMR (400 MHz, $CHCl_3$) (δ, ppm): 8.02-7.91 (m, 2H), 7.75-7.73 (m, 1H), 7.65-7.41 (m, 5H), 7.37-7.27 (m, 6H), 6.88 (s, 1H), 5.60 (s, 2H). ^{13}C NMR (100 MHz, $CDCl_3$) (δ, ppm): 193.6, 159.8, 141.4, 139.6, 136.4, 135.9, 133.8, 132.0, 131.3, 129.3, 128.9, 128.7, 127.5, 127.4, 127.3, 126.9, 122.9, 122.4, 118.3, 115.6, 46.2. HRMS (ESI) m/z calcd for $C_{25}H_{17}Cl_2NO_2Na$ $[M+Na]^+$ 456.0534, found 456.0528.

General procedure for the synthesis of compounds 2

Example for the synthesis of **2a**:

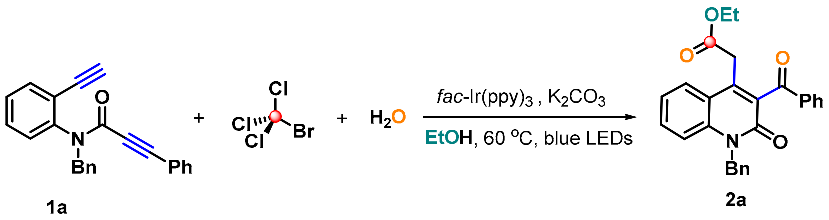

In a 25-mL Schlenk tube, 1,7-diyne **1a** (0.2 mmol, 67.0 mg, 1.0 equiv), $BrCCl_3$ (0.4 mmol, 79.2 mg, 2.0 equiv), K_2CO_3 (0.4 mmol, 55.2 mg, 2.0 equiv), *fac*-Ir(ppy)$_3$ (1.3 mg, 1 mol%) and EtOH/H_2O (2.0 mL, 100:1 V/V) were successively added under Ar conditions. Then, the tube was stirred at 60 °C in oil bath for 36 h under 30 W blue light (blue LEDs) irradiation until complete consumption of **1a** as monitored by TLC analysis. After the reaction was completed, the reaction mixture was concentrated in vacuum and the resulting residue was purified by column chromatography on silica gel (eluent, petroleum ether/ethyl acetate = 10:1) to afford the desired product **2a** (57.0 mg, 67%) as a white solid.

Ethyl 2-(3-benzoyl-1-benzyl-2-oxo-1,2-dihydroquinolin-4-yl)acetate (2a)

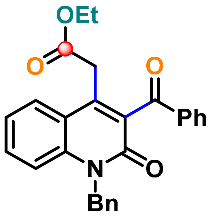

White solid after purification by column chromatography (petroleum ether/ethyl acetate = 10/1); 57.0 mg, 67% yield; mp: 153-155 °C; ^{1}H NMR (400 MHz, $CDCl_3$) (δ, ppm): 8.04-7.93 (m, 2H), 7.77 (d, *J* = 8.0 Hz, 1H), 7.60-7.56 (m, 1H), 7.53-7.44 (m, 3H), 7.39 (d, *J* = 8.4 Hz, 1H), 7.31-7.24 (m, 6H), 5.55 (s, 2H), 4.03 (q, *J* = 7.1 Hz, 2H), 3.90 (s, 2H), 1.08 (t, *J* = 7.1 Hz, 3H). ^{13}C NMR (100 MHz, $CDCl_3$) (δ, ppm): 195.2, 169.0, 160.1, 140.8, 139.3, 137.0, 136.0, 133.8, 132.7, 131.5, 129.4, 128.9, 128.6, 127.4, 126.8, 125.9, 122.8, 120.4, 115.6, 61.5, 46.0, 35.2, 13.9. HRMS (ESI) m/z calcd for $C_{27}H_{23}NO_4Na$ $[M+Na]^+$ 448.1525, found 448.1524.

Ethyl 2-(1-benzyl-3-(4-methylbenzoyl)-2-oxo-1,2-dihydroquinolin-4-yl)acetate (2b)

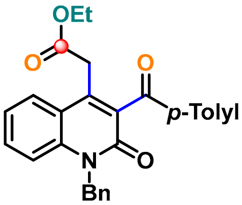

White solid after purification by column chromatography (petroleum ether/ethyl acetate = 10/1); 57.1 mg, 65% yield; mp: 162-164 °C; ^{1}H NMR (400 MHz, $CDCl_3$) (δ, ppm): 7.88 (d, *J* = 8.2 Hz, 1H), 7.76-7.74 (m, 1H), 7.52-7.48 (m, 1H), 7.39-7.37 (m, 1H), 7.34-7.26 (m, 5H), 7.24 (d, *J* = 4.1 Hz, 3H), 5.55 (s, 2H), 4.04 (q, *J* = 7.1 Hz, 2H), 3.89 (s, 2H), 2.40 (s, 3H), 1.10 (t, *J* = 7.1 Hz, 3H). ^{13}C NMR (100 MHz, $CDCl_3$) (δ, ppm): 194.7, 169.1, 160.1, 144.8,

140.56, 139.3, 136.0, 134.6, 133.0, 131.4, 129.6, 129.4, 128.8, 127.4, 126.8, 125.9, 122.8, 120.4, 115.6, 61.5, 46.0, 35.3, 21.8, 13.9. HRMS (ESI) m/z calcd for $C_{28}H_{25}NO_4Na$ $[M+Na]^+$ 462.1681, found 462.1677.

Ethyl 2-(1-benzyl-3-(3-methylbenzoyl)-2-oxo-1,2-dihydroquinolin-4-yl)acetate (2c)

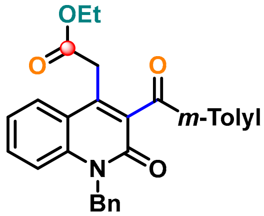

White solid after purification by column chromatography (petroleum ether/ethyl acetate = 10/1); 48.3 mg, 55% yield; mp: 159-161 °C; ^{1}H NMR (400 MHz, $CDCl_3$) (δ, ppm): 7.90 (d, *J* = 8.0 Hz, 2H), 7.78 (d, *J* = 8.0 Hz, 1H), 7.52 (t, *J* = 7.9 Hz, 1H), 7.40 (d, *J* = 8.5 Hz, 1H), 7.35-7.28 (m, 7H), 7.26 (s, 1H), 5.58 (s, 2H), 4.07 (q, *J* = 7.3 Hz, 2H), 3.91 (s, 2H), 2.43 (s, 3H), 1.12 (t, *J* = 7.1 Hz, 3H). ^{13}C NMR (100 MHz, $CDCl_3$) (δ, ppm): 195.3, 169.1, 156.6, 140.7, 139.4, 137.1, 133.7, 132.8, 131.5, 129.4, 128.6, 128.4, 127.2, 125.7, 123.9, 122.7, 120.9, 120.3, 115.8, 110.3, 61.5, 55.5, 40.6, 35.2, 13.9. HRMS (ESI) m/z calcd for $C_{28}H_{25}NO_4Na$ $[M+Na]^+$ 462.1681, found 462.1677.

Ethyl 2-(1-benzyl-3-(4-methoxybenzoyl)-2-oxo-1,2-dihydroquinolin-4-yl)acetate (2d)

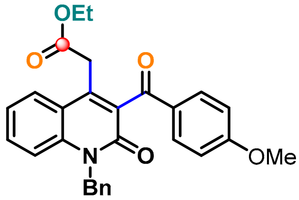

White solid after purification by column chromatography (petroleum ether/ethyl acetate = 10/1); 53.7 mg, 59% yield; mp: 165-167 °C; ^{1}H NMR (400 MHz, $CDCl_3$) (δ, ppm): 8.00-7.94 (m, 2H), 7.75-7.73 (m, 1H), 7.52-7.48 (m, 1H), 7.38 (d, *J* = 8.4 Hz, 1H), 7.34-7.26 (m, 4H), 7.24 (d, *J* = 3.0 Hz, 2H), 6.96-6.90 (m, 2H), 5.56 (s, 2H), 4.05 (q, *J* = 7.2 Hz, 2H), 3.87 (d, *J* = 8.7 Hz, 5H), 1.11 (t, *J* = 7.1 Hz, 3H). ^{13}C NMR (100 MHz, $CDCl_3$) (δ, ppm): 193.5, 169.2, 164.2, 160.1, 140.3, 139.2, 136.1, 133.1, 132.0, 131.3, 130.1, 128.8, 127.4, 126.8, 125.8, 122.7, 120.4, 115.6, 114.0, 61.5, 55.5, 46.0, 35.3, 14.0. HRMS (ESI) m/z calcd for $C_{28}H_{25}NO_5Na$ $[M+Na]^+$ 478.1630, found 478.1629.

Ethyl 2-(1-benzyl-3-(4-(tert-butyl)benzoyl)-2-oxo-1,2-dihydroquinolin-4-yl)acetate (2e)

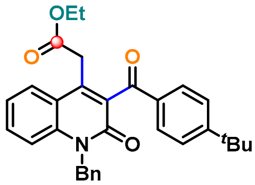

White solid after purification by column chromatography (petroleum ether/ethyl acetate = 10/1); 47.1 mg, 49% yield; mp: 164-166 °C; ^{1}H NMR (400 MHz, $CDCl_3$) (δ, ppm): 7.92 (d, *J* = 8.5 Hz, 1H), 7.77-7.74 (m, 1H), 7.52-7.45 (m, 3H), 7.40-7.39 (m, 1H), 7.33-7.26 (m, 5H), 7.24 (s, 1H), 5.56 (s, 2H), 4.03 (q, *J* = 7.1 Hz, 2H), 3.88 (s, 2H), 1.33 (s, 9H), 1.06 (t, *J* = 7.1 Hz, 3H). ^{13}C NMR (100 MHz, $CDCl_3$) (δ, ppm): 194.7, 169.1, 160.1, 157.6, 140.5, 139.3, 136.0, 134.3, 133.0, 131.3, 129.5, 128.8, 127.4, 126.8, 125.9, 125.7, 122.8, 120.4, 115.6, 61.4, 46.0, 35.3, 35.2, 31.1, 13.9. HRMS (ESI) m/z calcd for $C_{31}H_{31}NO_4Na$ $[M+Na]^+$504.2151, found 504.2148.

Ethyl 2-(1-benzyl-3-(4-fluorobenzoyl)-2-oxo-1,2-dihydroquinolin-4-yl)acetate (2f)

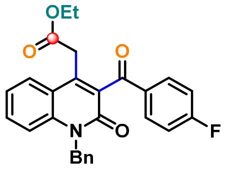

White solid after purification by column chromatography (petroleum ether/ethyl acetate = 10/1); 35.4 mg, 40% yield; mp: 194-196 °C; ^{1}H NMR (400 MHz, $CDCl_3$) (δ, ppm): 8.05-7.98 (m, 2H), 7.78-7.75 (m, 1H), 7.54-7.50 (m, 1H), 7.41-7.38 (m, 1H), 7.33-7.27 (m, 3H), 7.26-7.22 (m, 3H), 7.13 (t, *J* = 8.6 Hz, 2H), 5.55 (s, 2H), 4.05 (q, *J* = 7.1 Hz, 2H), 3.90 (s, 2H), 1.11 (t, *J* = 7.1 Hz, 3H). ^{13}C NMR (100 MHz, $CDCl_3$) (δ, ppm): 193.6, 169.0, 166.2 (d, *J* = 256.0 Hz), 160.0, 141.1, 139.3, 135.9, 133.5 (d, *J* = 2.8 Hz), 132.4, 132.2 (d, *J* = 9.5 Hz), 131.6, 128.9, 127.5, 126.8, 125.9, 122.9, 120.3, 115.95, 115.7 (d, *J* = 5.2 Hz), 61.6, 46.0, 35.2, 13.9. HRMS (ESI) m/z calcd for $C_{27}H_{22}FNO_4Na$ $[M+Na]^+$ 466.1431, found 466.1462.

Ethyl 2-(3-benzoyl-1-(2-methoxybenzyl)-2-oxo-1,2-dihydroquinolin-4-yl)acetate (2g)

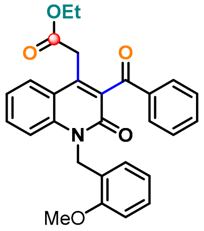

White solid after purification by column chromatography (petroleum ether/ethyl acetate = 10/1); 62.8 mg, 69% yield; mp: 150-152 °C; ^{1}H NMR (400 MHz, $CDCl_3$) (δ, ppm): 7.93-7.88 (m, 2H), 7.71-7.68 (m, 1H), 7.53-7.48 (m, 1H), 7.45-7.36 (m, 3H), 7.28 (d, *J* = 8.6 Hz, 1H), 7.21 (d, *J* = 7.7 Hz, 1H), 7.17-7.12 (m, 1H), 6.87-6.73 (m, 3H), 5.49 (s, 2H), 3.96 (q, *J* = 7.1 Hz, 2H), 3.88 (s, 3H), 3.84 (s, 2H), 1.01 (t, *J* = 7.1 Hz, 3H). ^{13}C NMR (100 MHz, $CDCl_3$) (δ, ppm): 194.7, 169.0, 160.0, 141.1, 139.3, 138.8, 136.8, 135.9, 132.0, 131.6, 129.2, 128.9, 127.5, 126.8, 126.0, 122.9, 120.4, 115.7, 98.0, 61.5, 59.6, 46.0, 35.2, 13.9. HRMS (ESI) m/z calcd for $C_{28}H_{25}NO_5Na$ $[M+Na]^+$ 478.1630, found 478.1625.

Ethyl 2-(3-benzoyl-1-(3-fluorobenzyl)-2-oxo-1,2-dihydroquinolin-4-yl)acetate (2h)

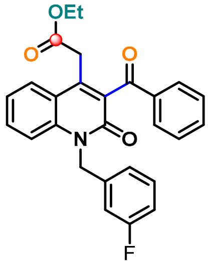

White solid after purification by column chromatography (petroleum ether/ethyl acetate = 10/1); 49.6 mg, 56% yield; mp: 168-170 °C; ^{1}H NMR (400 MHz, $CDCl_3$) (δ, ppm): 8.03-7.97 (m, 2H), 7.82-7.79 (m, 1H), 7.63-7.47 (m, 4H), 7.38-7.29 (m, 3H), 7.06 (d, *J* = 7.7 Hz, 1H), 6.96 (t, *J* = 7.5 Hz, 2H), 5.56 (s, 2H), 4.06 (q, *J* = 7.1 Hz, 2H), 3.93 (s, 2H), 1.11 (t, *J* = 7.1 Hz, 3H). ^{13}C NMR (100 MHz, $CDCl_3$) (δ, ppm): 195.1, 169.0, 163.1 (d, *J* = 246.7 Hz), 160.0, 141.1, 139.1, 138.5 (d, *J* = 7.2 Hz), 136.9, 133.9, 132.6, 131.6, 130.5 (d, *J* = 8.3 Hz), 129.4, 128.7, 126.1, 123.1, 122.4 (d, *J* = 2.9 Hz), 120.4, 115.4, 114.5 (d, *J* = 21.0 Hz), 113.8 (d, *J* = 22.4 Hz), 61.6, 45.5, 35.3, 13.9. HRMS (ESI) m/z calcd for $C_{27}H_{22}FNO_4Na$ $[M+Na]^+$ 466.1431, found 466.1433.

Ehyl 2-(3-benzoyl-1-(3-chlorobenzyl)-2-oxo-1,2-dihydroquinolin-4-yl)acetate (2i)

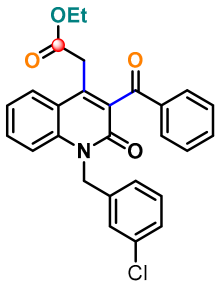

White solid after purification by column chromatography (petroleum ether/ethyl acetate = 10/1); 54.1 mg, 59% yield; mp: 173-175 °C; ^{1}H NMR (400 MHz, $CDCl_3$) (δ, ppm): 8.02-7.97 (m, 2H), 7.81 (d, *J* = 10.8 Hz, 1H), 7.65-7.47 (m, 4H), 7.36-7.30 (m, 2H), 7.26 (d, *J* = 4.0 Hz, 3H), 7.17-7.14 (m, 1H), 5.54 (s, 2H), 4.06 (q, *J* = 7.2 Hz, 2H), 3.93 (s, 2H), 1.11 (t, *J* = 7.2 Hz, 3H). ^{13}C NMR (100 MHz, $CDCl_3$) (δ, ppm): 195.1, 169.0, 160.0, 141.2, 139.1, 138.1, 136.9, 134.8, 133.9, 131.7, 130.2, 129.4, 128.7, 127.8, 126.9, 126.1, 125.0, 123.1, 120.4, 115.4, 61.6, 45.5, 35.3, 13.9. HRMS (ESI) m/z calcd for $C_{27}H_{22}ClNO_4Na$ $[M+Na]^+$ 482.1135, found 482.1133.

Ethyl 2-(3-benzoyl-1-(4-methylbenzyl)-2-oxo-1,2-dihydroquinolin-4-yl)acetate (2j)

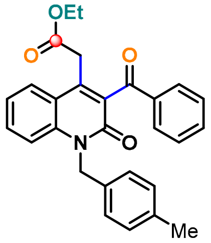

White solid after purification by column chromatography (petroleum ether/ethyl acetate = 10/1); 58.9 mg, 67% yield; mp: 126-128 °C; ^{1}H NMR (400 MHz, $CDCl_3$) (δ, ppm): 7.95-7.88 (m, 2H), 7.70-7.68 (m, 1H), 7.54-7.49 (m, 1H), 7.46-7.33 (m, 4H), 7.21 (d, *J* = 7.4 Hz, 1H), 7.11-7.02 (m, 4H), 5.44 (s, 2H), 3.96 (q, *J* = 7.2 Hz, 2H), 3.82 (s, 2H), 2.23 (s, 3H), 1.01 (t, *J* = 7.1 Hz, 3H). ^{13}C NMR (100 MHz, $CDCl_3$) (δ, ppm): 195.2, 169.0, 160.1, 140.7, 139.4, 137.1, 137.0, 133.7, 133.0, 131.4, 129.5, 129.4, 128.6, 126.8, 125.9, 122.7, 120.4, 115.6, 61.5, 45.8, 35.2, 21.1, 13.9. HRMS (ESI) m/z calcd for $C_{28}H_{25}NO_4Na$ $[M+Na]^+$ 462.1681, found 462.1682.

Ethyl 2-(3-benzoyl-1-(4-fluorobenzyl)-2-oxo-1,2-dihydroquinolin-4-yl)acetate (2k)

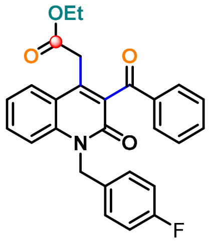

White solid after purification by column chromatography (petroleum ether/ethyl acetate = 10/1); 54.9 mg, 62% yield; mp: 175-177 °C; ^{1}H NMR (400 MHz, $CDCl_3$) (δ, ppm): 8.00-7.95 (m, 2H), 7.79-7.76 (m, 1H), 7.61-7.52 (m, 2H), 7.49-7.45 (m, 2H), 7.37 (d, *J* = 8.4 Hz, 1H), 7.32-7.28 (m, 1H), 7.26-7.21 (m, 2H), 7.02-6.97 (m, 2H), 5.51 (s, 2H), 4.03 (q, *J* = 7.1 Hz, 2H), 3.89 (s, 2H), 1.09 (t, *J* = 7.1 Hz, 3H). ^{13}C NMR (100 MHz, $CDCl_3$) (δ, ppm): 195.0, 169.0, 162.1 (d, *J* = 245.7 Hz), 160.0, 140.9, 139.2, 136.9, 133.8, 132.8, 131.7 (d, *J* = 3.2 Hz), 131.5, 129.4, 128.7, 128.6 (d, *J* = 5.2 Hz), 126.1, 122.9, 120.4, 115.8 (d, *J* = 21.6 Hz), 115.4, 61.5, 45.3, 35.2, 13.9. HRMS (ESI) m/z calcd for $C_{27}H_{22}FNO_4Na$ $[M+Na]^+$ 466.1431, found 466.1433.

Ethyl 2-(3-benzoyl-1-(4-chlorobenzyl)-2-oxo-1,2-dihydroquinolin-4-yl)acetate (2l)

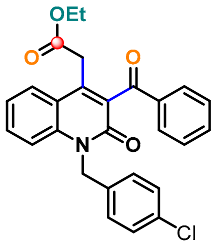

White solid after purification by column chromatography (petroleum ether/ethyl acetate = 10/1); 43.1 mg, 47% yield; mp: 163-165 °C; ^{1}H NMR (400 MHz, $CDCl_3$) (δ, ppm): 8.01-7.95 (m, 2H), 7.79-7.77 (m, 1H), 7.61-7.45 (m, 4H), 7.35-7.27 (m, 4H), 7.20 (d, *J* = 8.4 Hz, 2H), 5.51 (s, 2H), 4.04 (q, *J* = 7.1 Hz, 2H), 3.90 (s, 2H), 1.09 (t, *J* = 7.1 Hz, 3H). ^{13}C NMR (100 MHz, $CDCl_3$) (δ, ppm): 195.0, 169.0, 160.0, 141.0, 139.1, 136.9, 134.5, 133.9, 133.3, 132.7, 131.6, 129.4, 129.0, 128.7, 128.3, 126.1, 123.0, 120.4, 115.4, 61.5, 45.4, 35.2, 13.9. HRMS (ESI) m/z calcd for $C_{27}H_{22}ClNO_4Na$ $[M+Na]^+$ 482.1135, found 482.1138.

Ethyl 2-(3-benzoyl-1-(4-bromobenzyl)-2-oxo-1,2-dihydroquinolin-4-yl)acetate (2m)

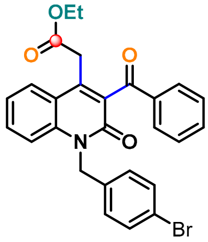

White solid after purification by column chromatography (petroleum ether/ethyl acetate = 10/1); 52.3 mg, 52% yield; mp: 177-179 °C; ^{1}H NMR (400 MHz, $CDCl_3$) (δ, ppm): 8.03-7.97 (m, 2H), 7.81 (d, *J* = 8.0 Hz, 1H), 7.65-7.47 (m, 4H), 7.34 (d, *J* = 8.6 Hz, 2H), 7.26 (d, *J* = 3.1 Hz, 3H), 7.17-7.14 (m, 1H), 5.54 (s, 2H), 4.06 (q, *J* = 7.2 Hz, 2H), 3.93 (s, 2H), 1.11 (t, *J* = 7.1 Hz, 3H). ^{13}C NMR (100 MHz, $CDCl_3$) (δ, ppm): 195.0, 168.9, 160.0, 141.0, 139.1, 136.9, 135.1, 133.8, 132.7, 132.0, 131.5, 129.4, 128.7, 128.6, 126.1, 123.0, 121.4, 120.4, 115.3, 61.5, 45.5, 35.2, 13.9. HRMS (ESI) m/z calcd for $C_{27}H_{22}BrNO_4Na$ $[M+Na]^+$ 526.0630, found 526.0631.

Ethyl 2-(3-benzoyl-1-benzyl-6-methyl-2-oxo-1,2-dihydroquinolin-4-yl)acetate (2n)

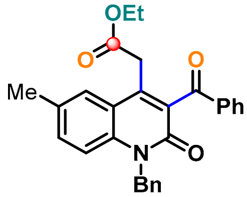

White solid after purification by column chromatography (petroleum ether/ethyl acetate = 10/1); 48.3 mg, 55% yield; mp: 175-177 °C; ^{1}H NMR (400 MHz, $CDCl_3$) (δ, ppm): 7.83-7.76 (m, 3H), 7.56-7.51 (m, 1H), 7.44-7.29 (m, 7H), 7.27 (d, *J* = 2.4 Hz, 2H), 5.58 (s, 2H), 4.06 (q, *J* = 7.1 Hz, 2H), 3.91 (s, 2H), 2.41 (s, 3H), 1.11 (t, *J* = 7.1 Hz, 3H). ^{13}C NMR (100 MHz, $CDCl_3$) (δ, ppm): 195.3, 169.0, 160.1, 140.6, 139.3, 138.4, 137.0, 136.1, 134.6, 132.9, 131.4, 129.8, 128.8, 128.5, 127.4, 126.8, 126.7, 125.9, 122.8, 120.4, 115.6, 61.4, 45.9, 35.3, 21.3, 13.9. HRMS (ESI) m/z calcd for $C_{28}H_{25}NO_4Na$ $[M+Na]^+$ 462.1681, found 462.1704.

Ethyl 2-(3-benzoyl-1-benzyl-7-methyl-2-oxo-1,2-dihydroquinolin-4-yl)acetate (2o)

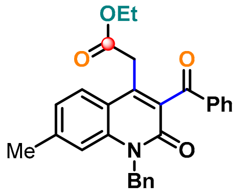

White solid after purification by column chromatography (petroleum ether/ethyl acetate = 10/1); 53.6 mg, 61% yield; mp: 151-153 °C; ^{1}H NMR (400 MHz, $CDCl_3$) (δ, ppm): 8.05-7.98 (m, 2H), 7.69 (d, *J* = 8.4 Hz, 1H), 7.65-7.58 (m, 1H), 7.51-7.48 (m, 2H), 7.38-7.32 (m, 2H), 7.30-7.26 (m, 3H), 7.23 (s, 1H), 7.15-7.12 (m, 1H), 5.57 (s, 2H), 4.06 (q, *J* = 7.1 Hz, 2H), 3.92 (s, 2H), 2.44 (s, 3H), 1.12 (t, *J* = 7.1 Hz, 3H). ^{13}C NMR (100 MHz, $CDCl_3$) (δ, ppm): 195.4, 169.1, 160.2, 142.3, 140.9, 139.5, 137.2, 136.1, 133.6, 131.6, 129.4, 128.8, 128.6, 127.4, 126.8, 125.8, 124.2, 118.2, 115.7, 61.4, 45.9, 35.2, 22.1, 13.9. HRMS (ESI) m/z calcd for $C_{28}H_{25}NO_4Na$ $[M+Na]^+$ 462.1681, found 462.1682.

Methyl 2-(3-benzoyl-1-benzyl-2-oxo-1,2-dihydroquinolin-4-yl)acetate (2p)

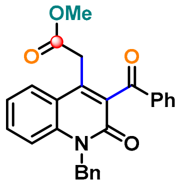

White solid after purification by column chromatography (petroleum ether/ethyl acetate = 10/1); 53.4 mg, 65% yield; mp: 157-159 °C; ^{1}H NMR (400 MHz, $CDCl_3$) (δ, ppm): 8.05-7.99 (m, 2H), 7.81-7.78 (m, 1H), 7.65-7.61 (m, 1H), 7.58-7.48 (m, 3H), 7.45-7.42 (m, 1H), 7.38-7.29 (m, 6H), 5.59 (s, 2H), 3.95 (s, 2H), 3.61 (s, 3H). ^{13}C NMR (100 MHz, $CDCl_3$) (δ, ppm): 195.1, 169.4, 160.0, 140.6, 139.4, 137.0, 136.0, 133.8, 132.8, 131.5, 129.4, 128.9, 128.6, 127.5, 126.8, 125.8, 122.9, 120.3, 115.6, 52.4, 46.0, 34.9. HRMS (ESI) m/z calcd for $C_{26}H_{21}NO_4Na$ $[M+Na]^+$ 434.1368, found 434.1371.

Isopropyl 2-(3-benzoyl-1-benzyl-2-oxo-1,2-dihydroquinolin-4-yl)acetate (2q)

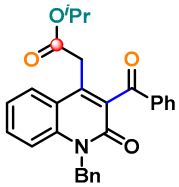

White solid after purification by column chromatography (petroleum ether/ethyl acetate = 10/1); 56.2 mg, 64% yield; mp: 151-153 °C; ^{1}H NMR (400 MHz, $CDCl_3$) (δ, ppm): 8.02-7.97 (m, 2H), 7.77-7.75 (m, 1H), 7.60-7.57 (m, 1H), 7.51-7.44 (m, 3H), 7.39 (d, *J* = 8.4 Hz, 1H), 7.33-7.27 (m, 3H), 7.26-7.22 (m, 3H), 5.56 (s, 2H), 4.95-4.86 (m, 1H), 3.87 (s, 2H), 1.07 (d, *J* = 6.2 Hz, 6H). ^{13}C NMR (100 MHz, $CDCl_3$) (δ, ppm): 195.1, 168.6, 160.1, 141.0, 139.3, 137.0, 136.0, 133.7, 132.7, 131.4, 129.4, 128.8, 128.6, 127.4, 126.8, 125.9, 122.7, 120.4, 115.6, 69.2, 45.9, 35.6, 21.5. HRMS (ESI) m/z calcd for $C_{28}H_{25}NO_4Na$ $[M+Na]^+$ 462.1681, found 462.1688.

Isobutyl 2-(3-benzoyl-1-benzyl-2-oxo-1,2-dihydroquinolin-4-yl)acetate (2r)

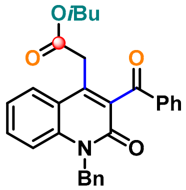

White solid after purification by column chromatography (petroleum ether/ethyl acetate = 10/1); 54.4 mg, 60% yield; mp: 144-146 °C; ^{1}H NMR (400 MHz, $CDCl_3$) (δ, ppm): 8.04-8.00 (m, 2H), 7.82-7.80 (m, 1H), 7.65-7.59 (m, 1H), 7.57-7.48 (m, 3H), 7.43-7.41 (m, 1H), 7.36-7.33 (m, 3H), 7.30-7.26 (m, 3H), 5.60 (s, 2H), 3.95 (s, 2H), 3.80 (d, J = 6.6 Hz, 2H), 1.84-1.74 (m, 1H), 0.78 (d, J = 6.8 Hz, 6H). ^{13}C NMR (100 MHz, $CDCl_3$) (δ, ppm): 195.0, 169.0, 160.0, 140.8, 139.3, 136.9, 136.0, 133.8, 132.7, 131.4, 129.4, 128.8, 128.7, 127.4, 126.8, 125.9, 122.8, 120.4, 115.6, 71.5, 45.9, 35.3, 27.5, 18.8. HRMS (ESI) m/z calcd for $C_{29}H_{27}NO_4Na$ $[M+Na]^+$ 476.1838, found 462.1849.

Cyclohexyl 2-(3-benzoyl-1-benzyl-2-oxo-1,2-dihydroquinolin-4-yl)acetate (2s)

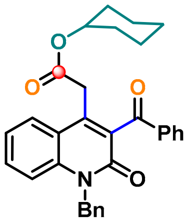

White solid after purification by column chromatography (petroleum ether/ethyl acetate = 10/1); 48.9 mg, 51% yield; mp: 148-150 °C; ^{1}H NMR (400 MHz, $CDCl_3$) (δ, ppm): 8.02-7.98 (m, 2H), 7.77-7.75 (m, 1H), 7.60-7.55 (m, 1H), 7.51-7.44 (m, 3H), 7.38 (d, J = 8.4 Hz, 1H), 7.32-7.28 (m, 3H), 7.25-7.21 (m, 3H), 5.56 (s, 2H), 4.71-4.66 (m, 1H), 3.88 (s, 2H), 1.64 (q, J = 5.6 Hz, 2H), 1.55-1.45 (m, 2H), 1.31-1.18 (m, 6H). ^{13}C NMR (100 MHz, $CDCl_3$) (δ, ppm): 195.2, 168.5, 160.1, 141.1, 139.2, 137.0, 136.0, 133.8, 132.6, 131.4, 129.5, 129.3, 128.8, 128.7, 127.4, 126.8, 126.8, 125.9, 122.7, 120.4, 115.6, 73.9, 45.9, 35.7, 31.2, 25.2, 23.4. HRMS (ESI) m/z calcd for $C_{31}H_{29}NO_4Na$ $[M+Na]^+$ 502.1994, found 502.1998.

But-3-yn-1-yl 2-(3-benzoyl-1-benzyl-2-oxo-1,2-dihydroquinolin-4-yl)acetate (2t)

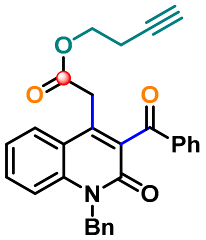

White solid after purification by column chromatography (petroleum ether/ethyl acetate = 10/1); 54.8 mg, 61% yield; mp: 135-137 °C; ^{1}H NMR (400 MHz, $CDCl_3$) (δ, ppm): 8.08-7.97 (m, 2H), 7.81 (d, J = 8.0 Hz, 1H), 7.65-7.61 (m, 1H), 7.57-7.49 (m, 3H), 7.43 (d, J = 8.4 Hz, 1H), 7.37-7.30 (m, 4H), 7.29-7.26 (m, 2H), 5.59 (s, 2H), 4.15-4.11 (m, 2H), 3.97 (s, 2H), 2.41-2.37 (m, 2H), 1.85-1.84 (m, 1H). ^{13}C NMR (100 MHz, $CDCl_3$) (δ, ppm): 195.1, 168.8, 160.0, 140.5, 139.3, 137.0, 136.0, 133.8, 132.9, 131.5, 129.4, 128.8, 128.7, 127.5, 126.8, 125.9, 122.8, 120.3, 115.6, 79.4, 69.9, 62.9, 53.4, 35.0, 18.7. HRMS (ESI) m/z calcd for $C_{29}H_{23}NO_4Na$ $[M+Na]^+$ 472.1525, found 472.1528.

Benzyl 2-(3-benzoyl-1-benzyl-2-oxo-1,2-dihydroquinolin-4-yl)acetate (2u)

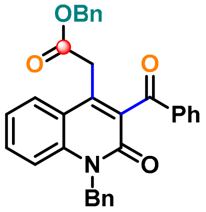

White solid after purification by column chromatography (petroleum ether/ethyl acetate = 10/1); 56.5 mg, 58% yield; mp: 161-163 °C; ^{1}H NMR (400 MHz, $CDCl_3$) (δ, ppm): 8.02-7.97 (m, 2H), 7.75-7.73 (m, 1H), 7.62-7.52 (m, 2H), 7.48-7.42 (m, 3H), 7.37-7.32 (m, 4H), 7.30-7.23 (m, 5H), 7.19-7.13 (m, 2H), 5.59 (s, 2H), 5.06 (s, 2H), 3.99 (s, 2H). ^{13}C NMR (100 MHz, $CDCl_3$) (δ, ppm): 195.1, 168.9, 160.0, 140.6, 139.3, 136.9, 136.0, 135.2, 133.8, 132.8, 131.5, 129.4, 128.9, 128.6, 128.5, 128.3, 128.1, 127.4, 126.8, 125.9, 122.8, 120.3, 115.6, 67.1, 53.4, 35.3. HRMS (ESI) m/z calcd for $C_{32}H_{25}NO_4Na$ $[M+Na]^+$ 510.1681, found 510.1681.

***S*-ethyl 2-(3-benzoyl-1-benzyl-2-oxo-1,2-dihydroquinolin-4-yl)ethanethioate (2v)**

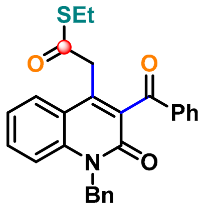

White solid after purification by column chromatography (petroleum ether/ethyl acetate = 10/1); 55.6 mg, 63% yield; mp: 136-138 °C; ^{1}H NMR (400 MHz, $CDCl_3$) (δ, ppm): 8.03-8.00 (m, 2H), 7.81-7.79 (m, 1H), 7.64-7.60 (m, 1H), 7.57-7.47 (m, 4H), 7.43-7.41 (m, 1H), 7.34 (d, J = 6.6 Hz, 3H), 7.29 (d, J = 3.9 Hz, 2H), 5.59 (s, 2H), 4.17 (s, 2H), 2.85 (q, J = 7.4 Hz, 2H), 1.18 (t, J = 7.4 Hz, 3H). ^{13}C NMR (100 MHz, $CDCl_3$) (δ, ppm): 195.0, 194.4, 160.0, 140.7, 139.3, 137.0, 136.0, 133.8, 133.1, 131.5, 129.5, 129.2, 128.9, 128.8, 128.8, 128.7, 127.4, 126.8, 126.3, 122.8, 120.4, 115.6, 53.4, 46.0, 23.9, 14.3. HRMS (ESI) m/z calcd for $C_{27}H_{23}NO_3SNa$ $[M+Na]^+$ 464.1296, found 464.1320.

***S*-isopropyl 2-(3-benzoyl-1-benzyl-2-oxo-1,2-dihydroquinolin-4-yl)ethanethioate (2w)**

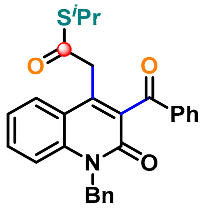

White solid after purification by column chromatography (petroleum ether/ethyl acetate = 10/1); 64.6 mg, 71% yield; mp: 158-160 °C; ^{1}H NMR (300 MHz, $CDCl_3$) (δ, ppm): 7.91 (d, J = 7.4 Hz, 2H), 7.69 (d, J = 8.1 Hz, 1H), 7.55-7.29 (m, 6H), 7.26-7.20 (m, 3H), 7.19-7.14 (m, 2H), 5.48 (s, 2H), 4.03 (s, 2H), 3.53-3.43 (m, 1H), 1.12 (d, J = 6.9 Hz, 6H). ^{13}C NMR (100 MHz, $CDCl_3$) (δ, ppm): 195.0, 194.4, 160.0, 140.8, 139.3, 137.0, 136.0, 133.8, 133.1, 131.5, 129.5, 128.8, 128.6, 127.4, 126.8, 126.3, 122.7, 120.4, 115.6, 46.0, 44.0, 35.5, 22.6, 18.4. HRMS (ESI) m/z calcd for $C_{28}H_{25}NO_3SNa$ $[M+Na]^+$ 478.1453, found 478.1476.

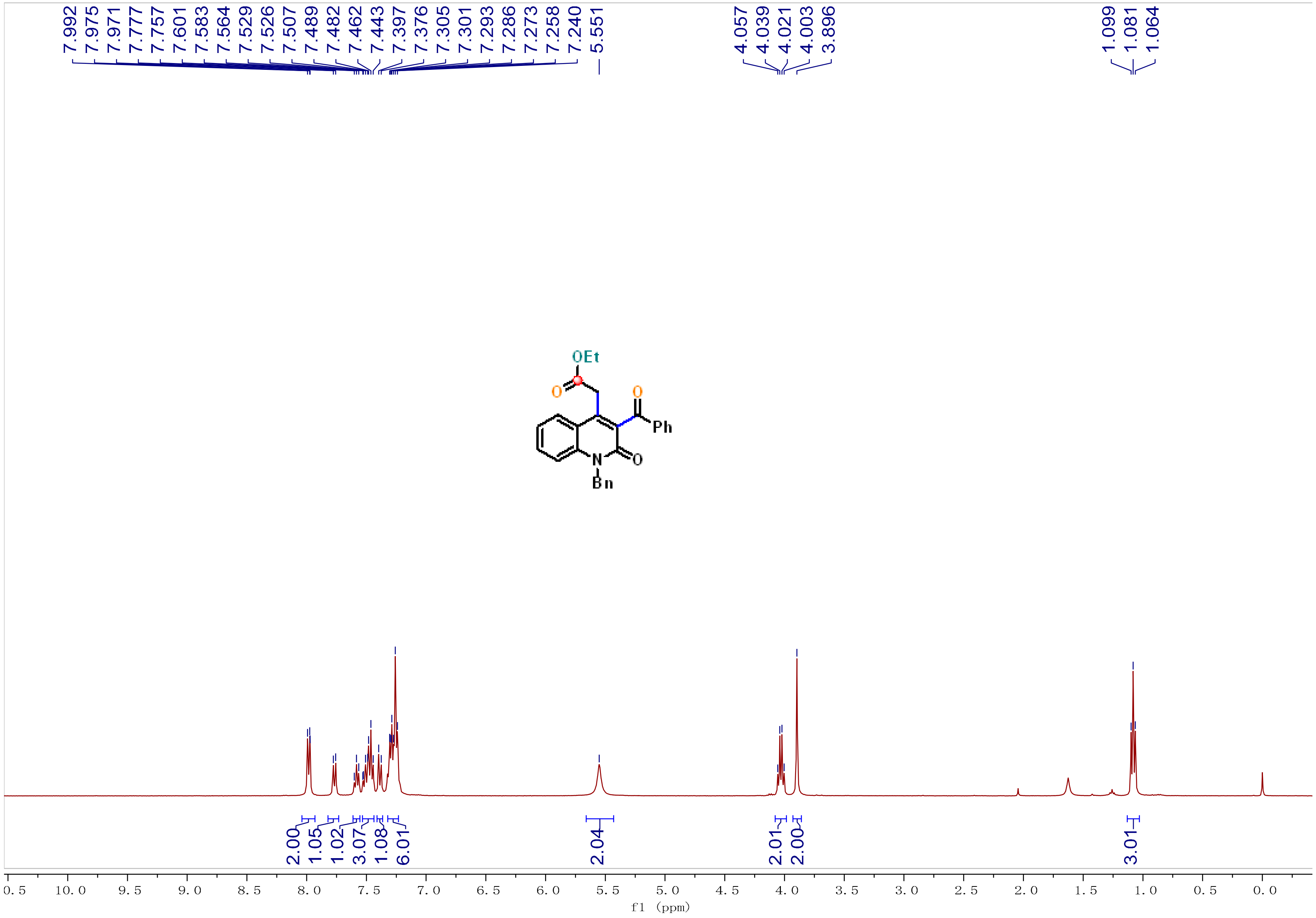

^{1}H NMR Spectrum of Compound 2a

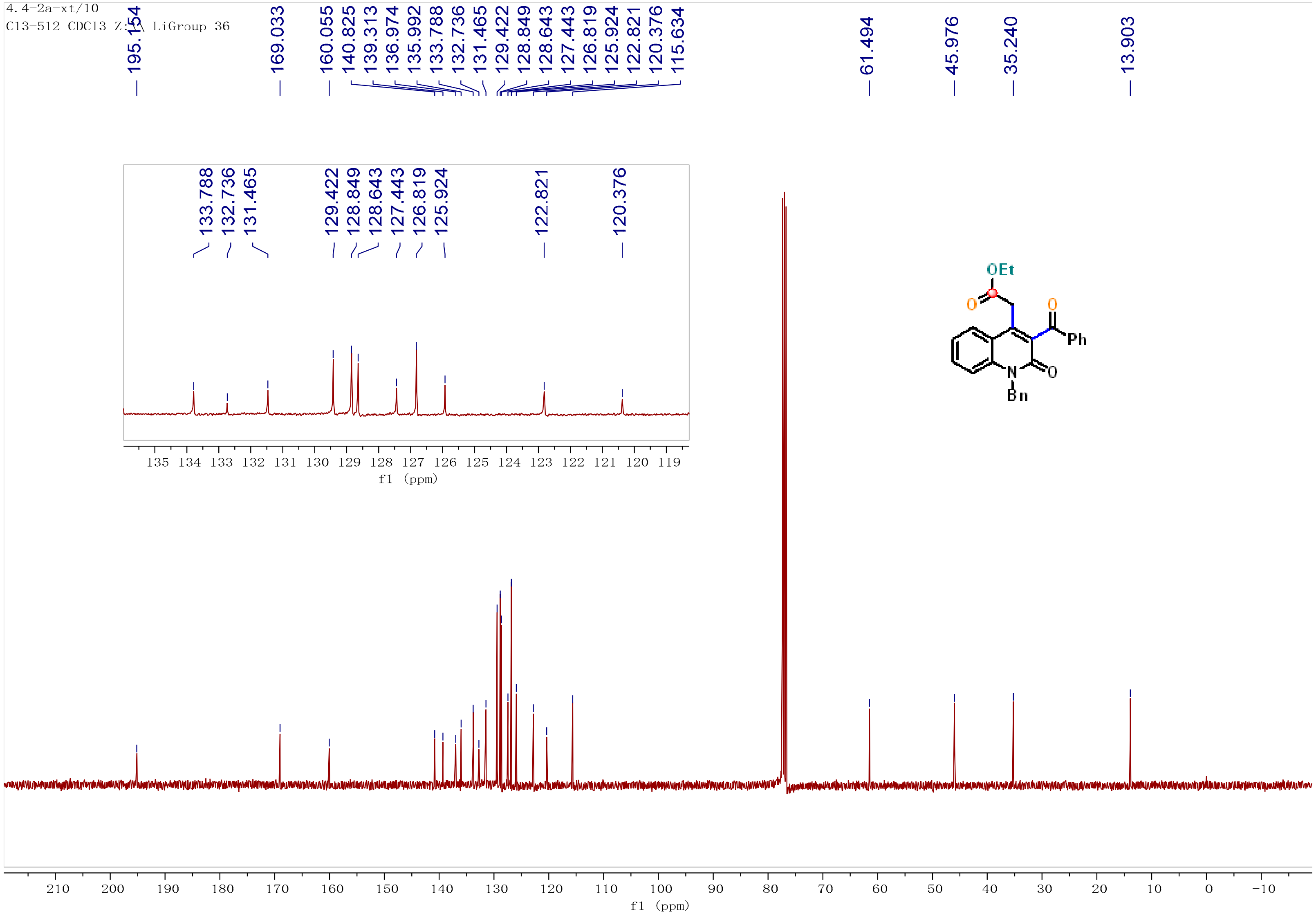

^{13}C NMR Spectrum of Compound 2a

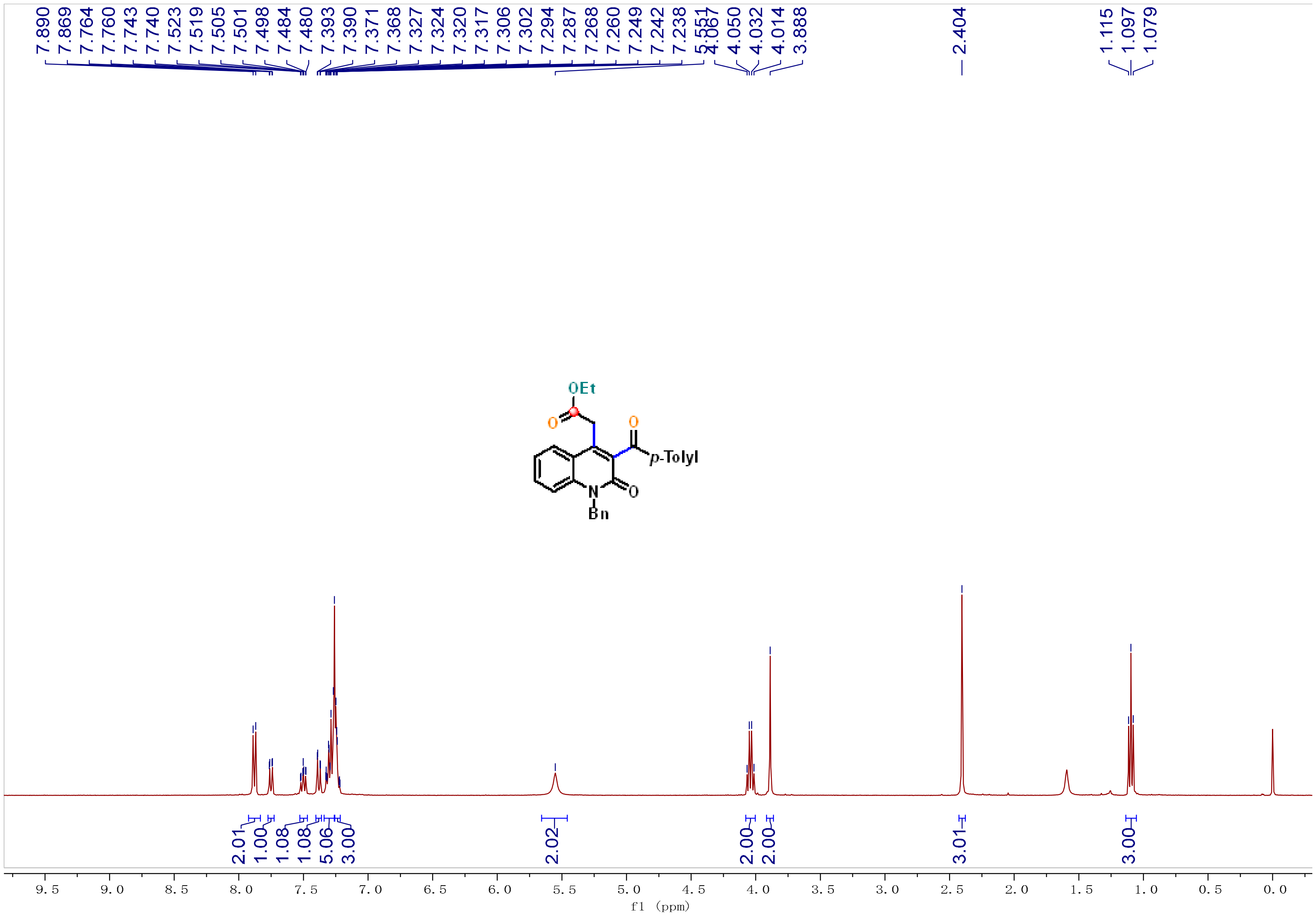

¹H NMR Spectrum of Compound 2b

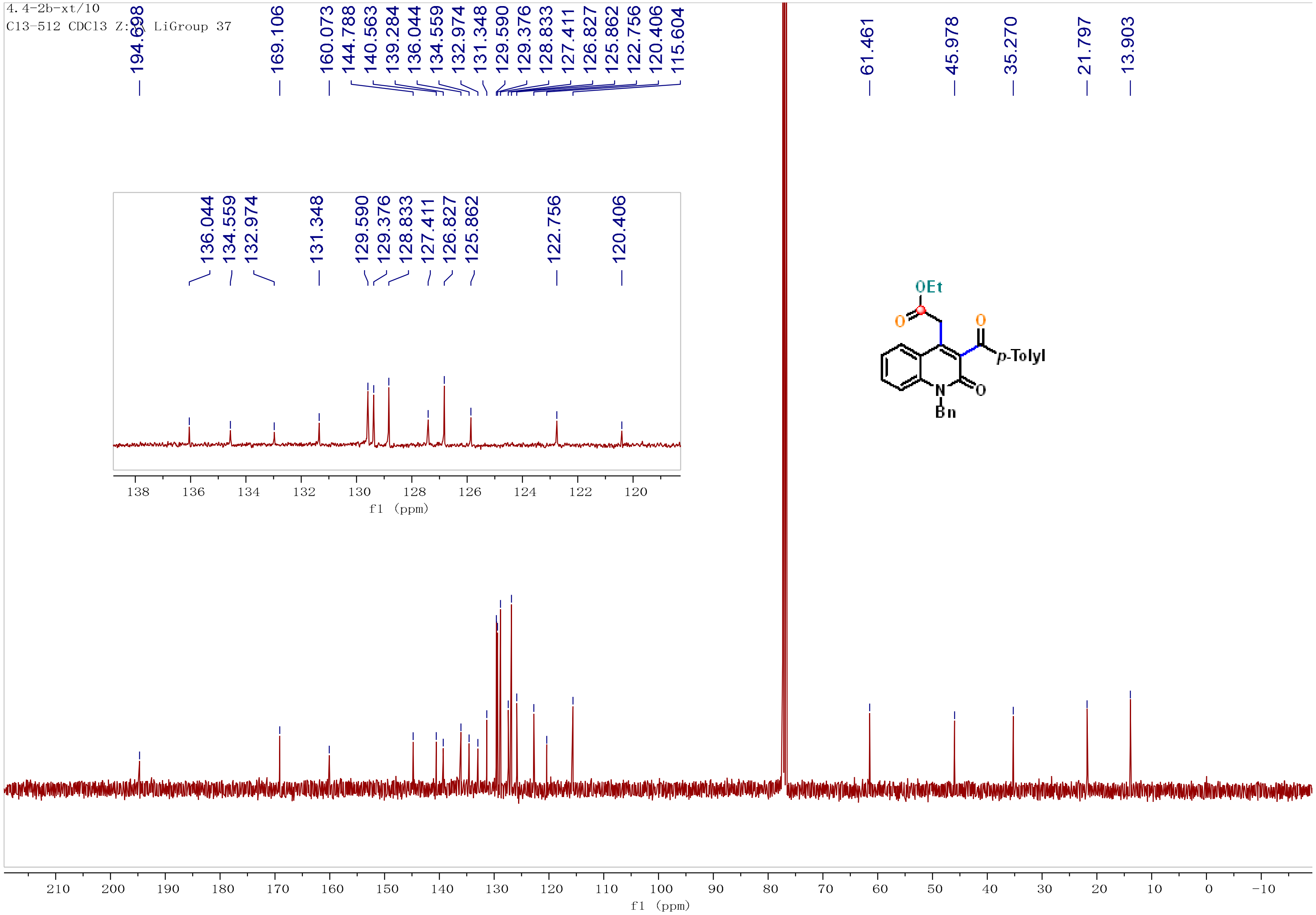

^{13}C NMR Spectrum of Compound 2b

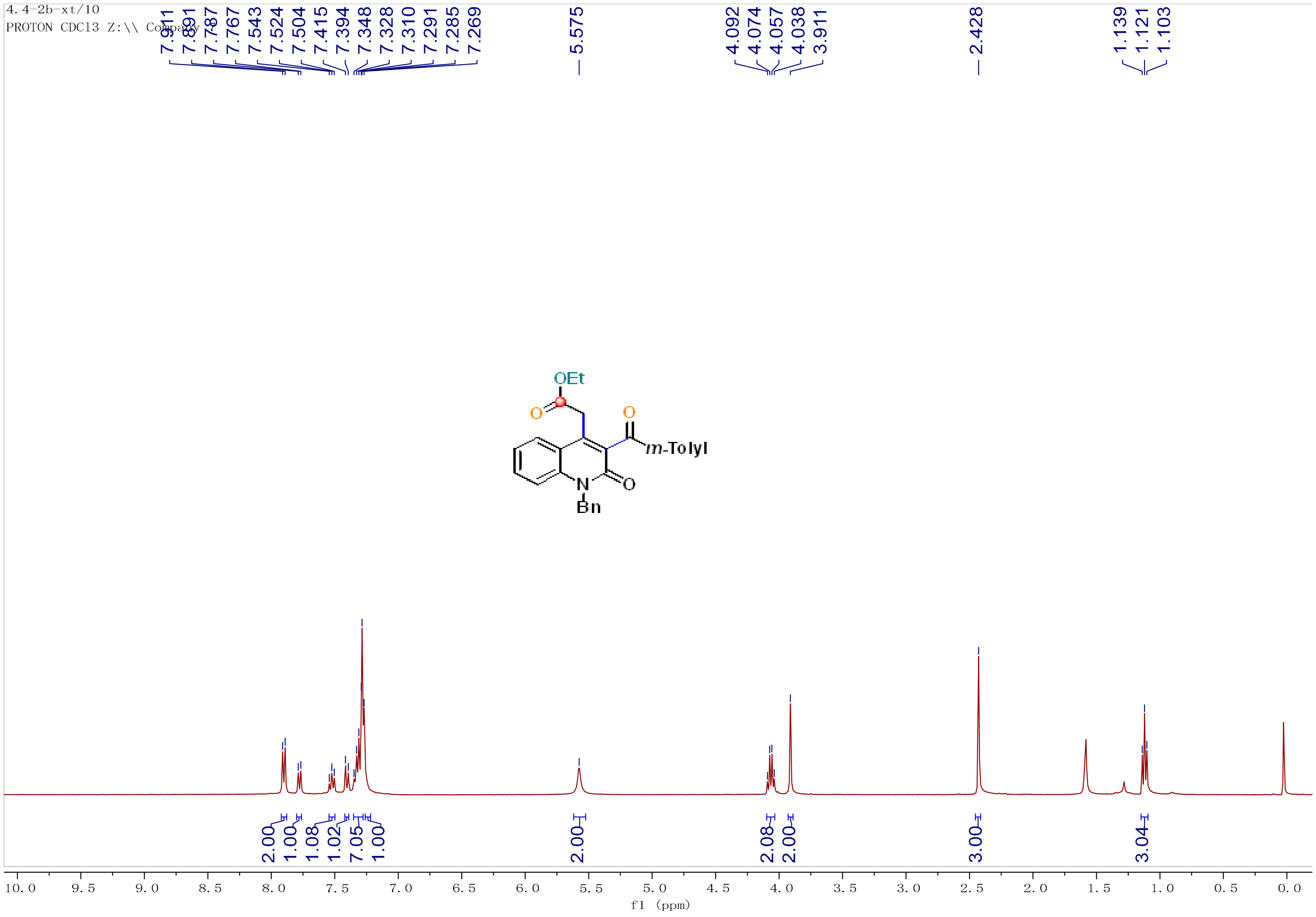

¹H NMR Spectrum of Compound 2c

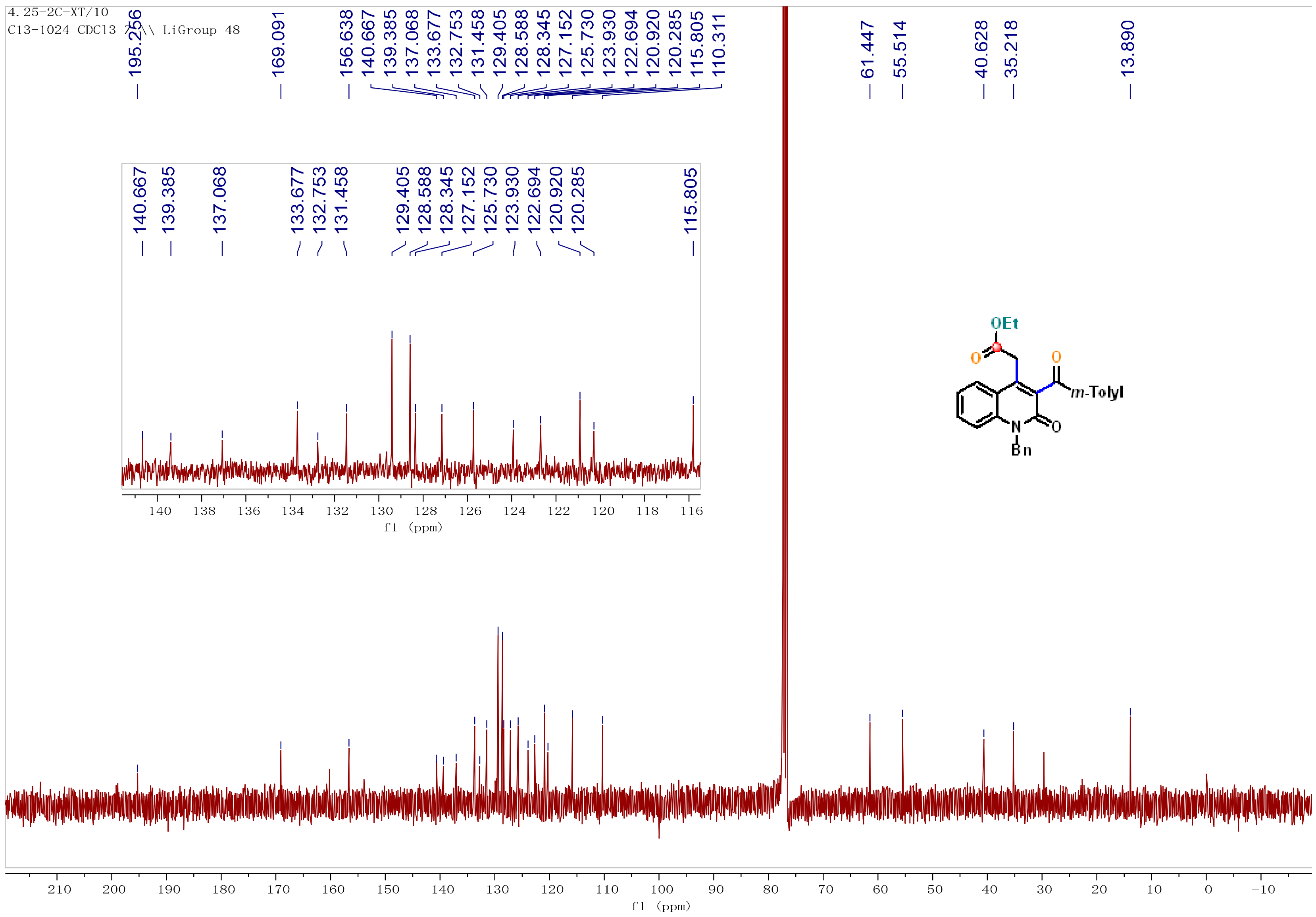

^{13}C NMR Spectrum of Compound 2c

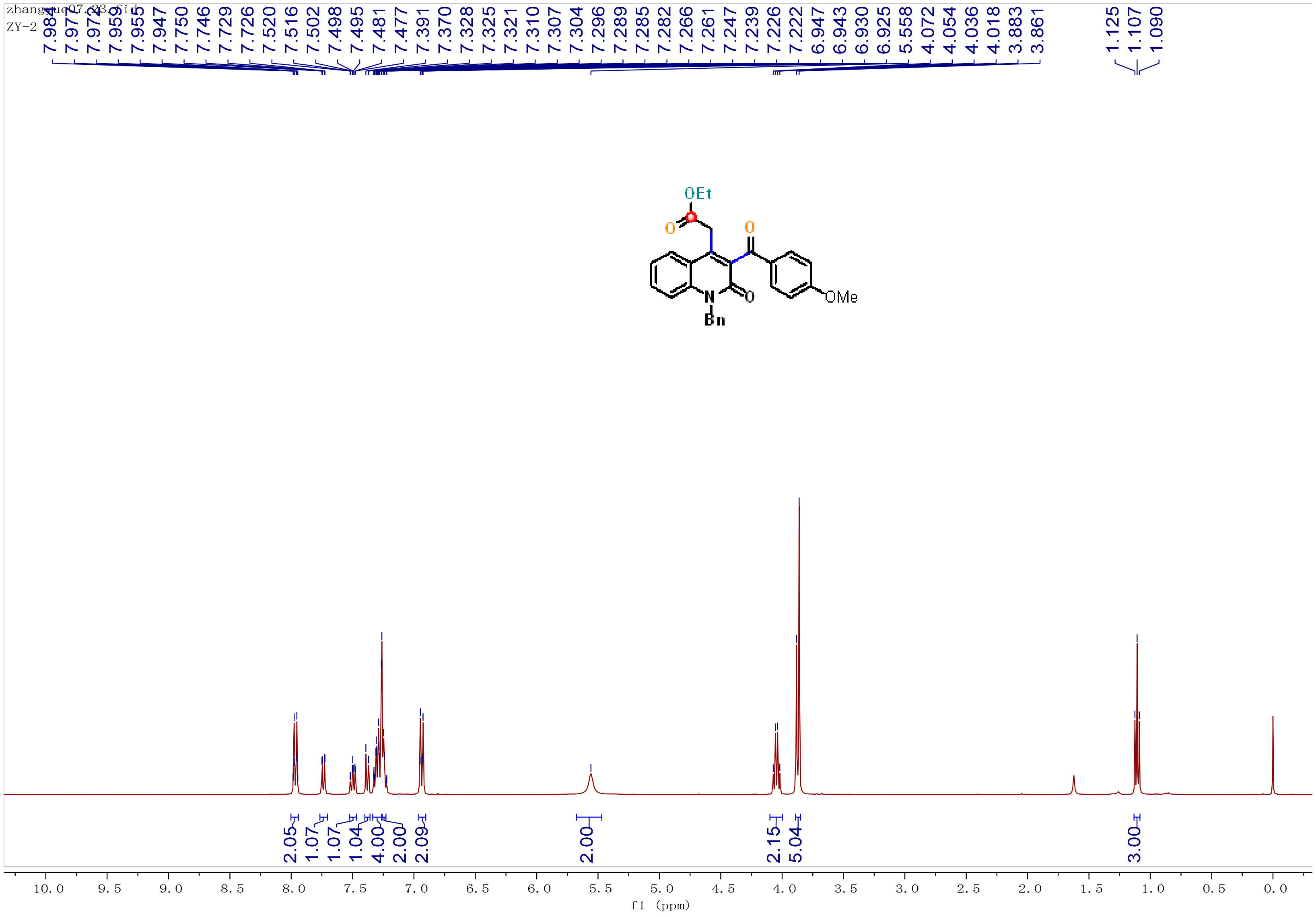

¹H NMR Spectrum of Compound 2d

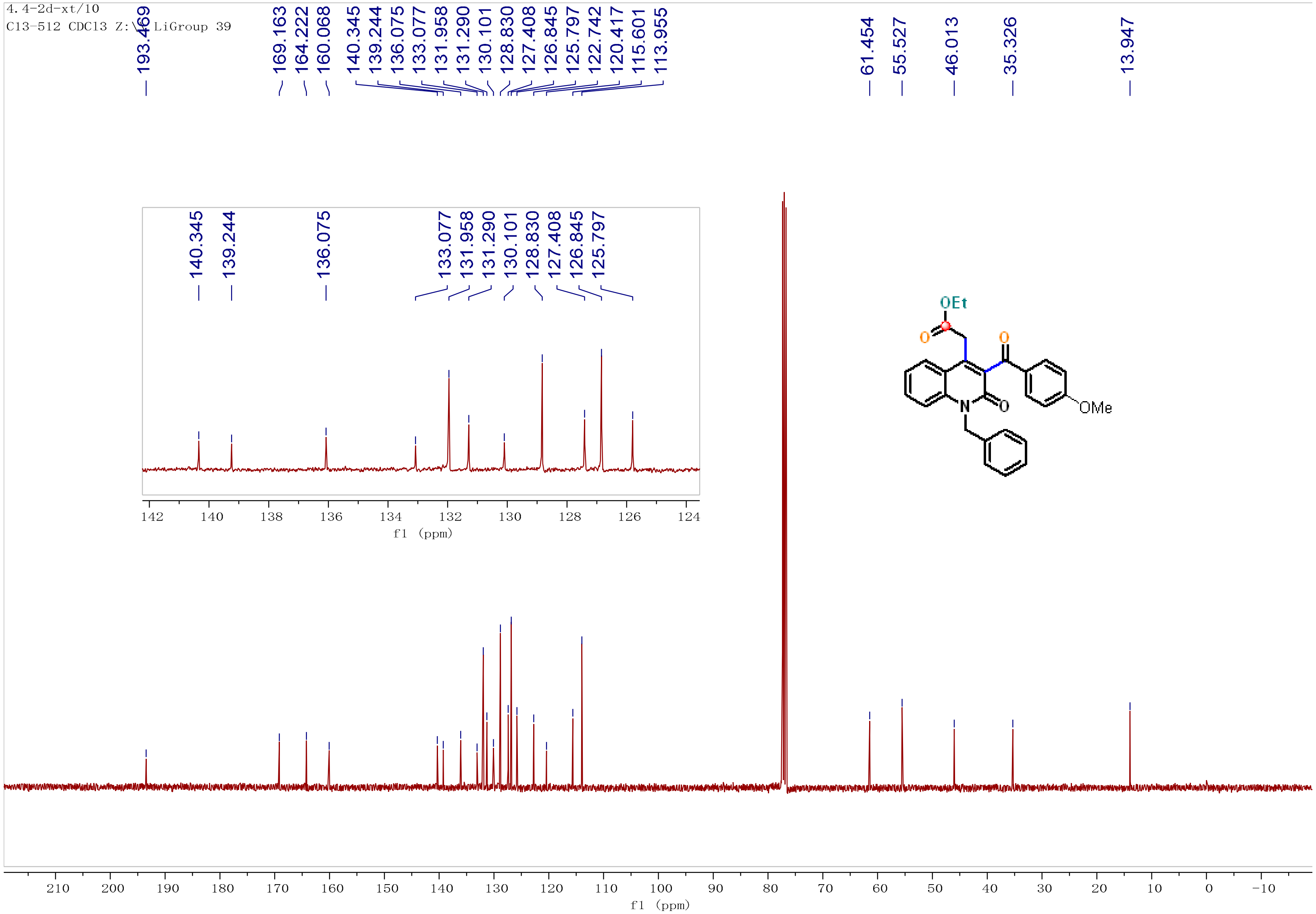

^{13}C NMR Spectrum of Compound 2d

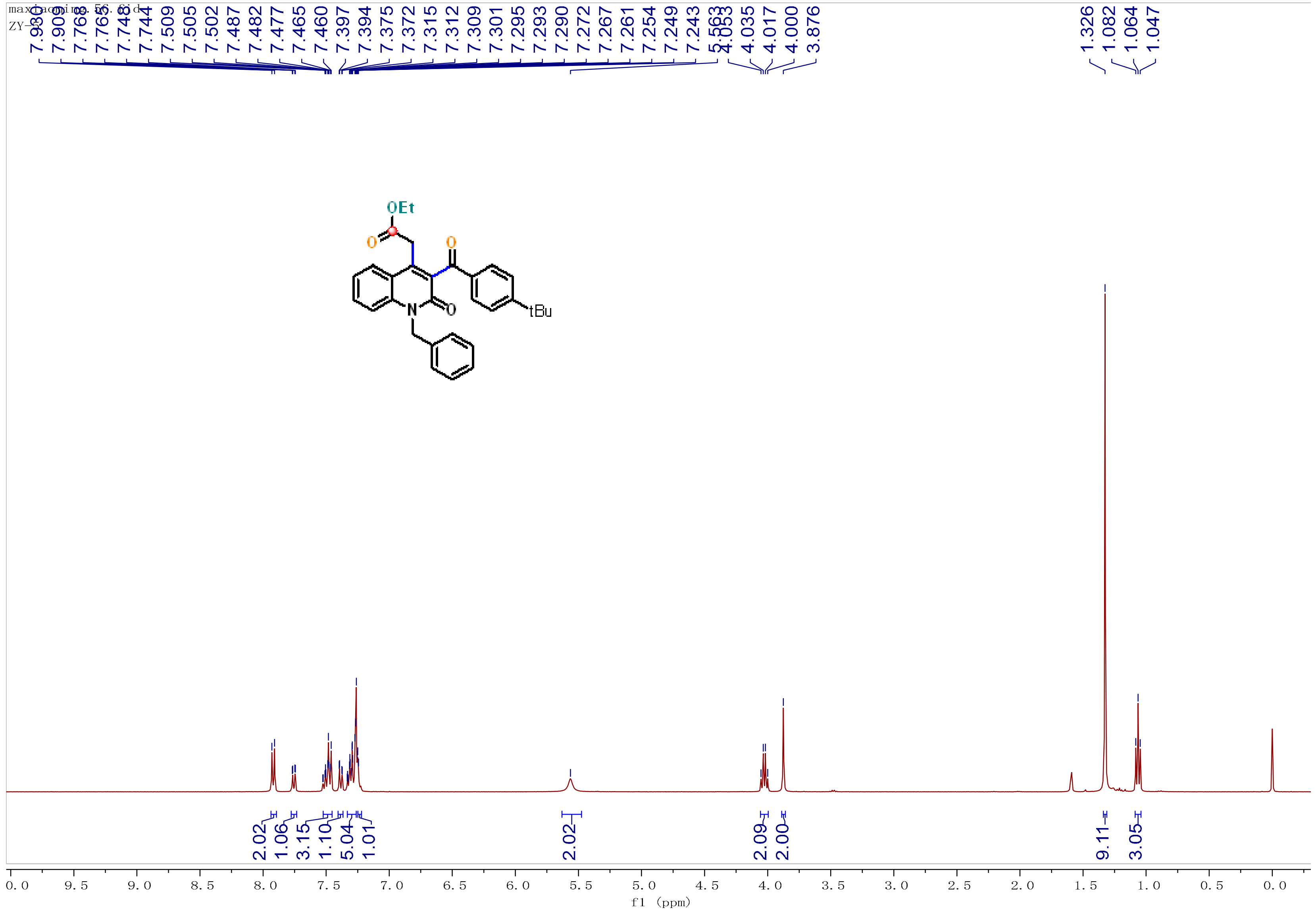

¹H NMR Spectrum of Compound 2e

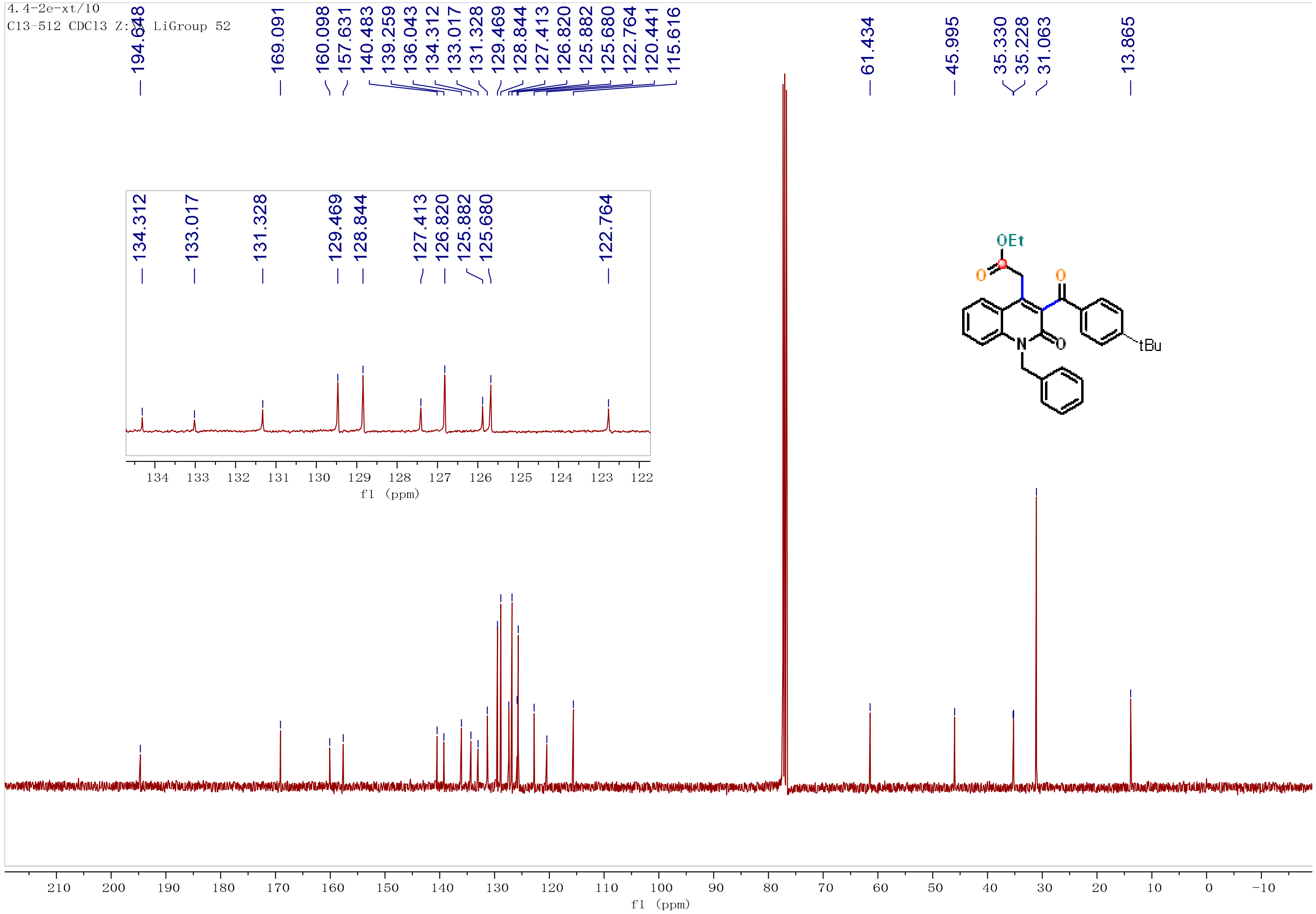

^{13}C NMR Spectrum of Compound 2e

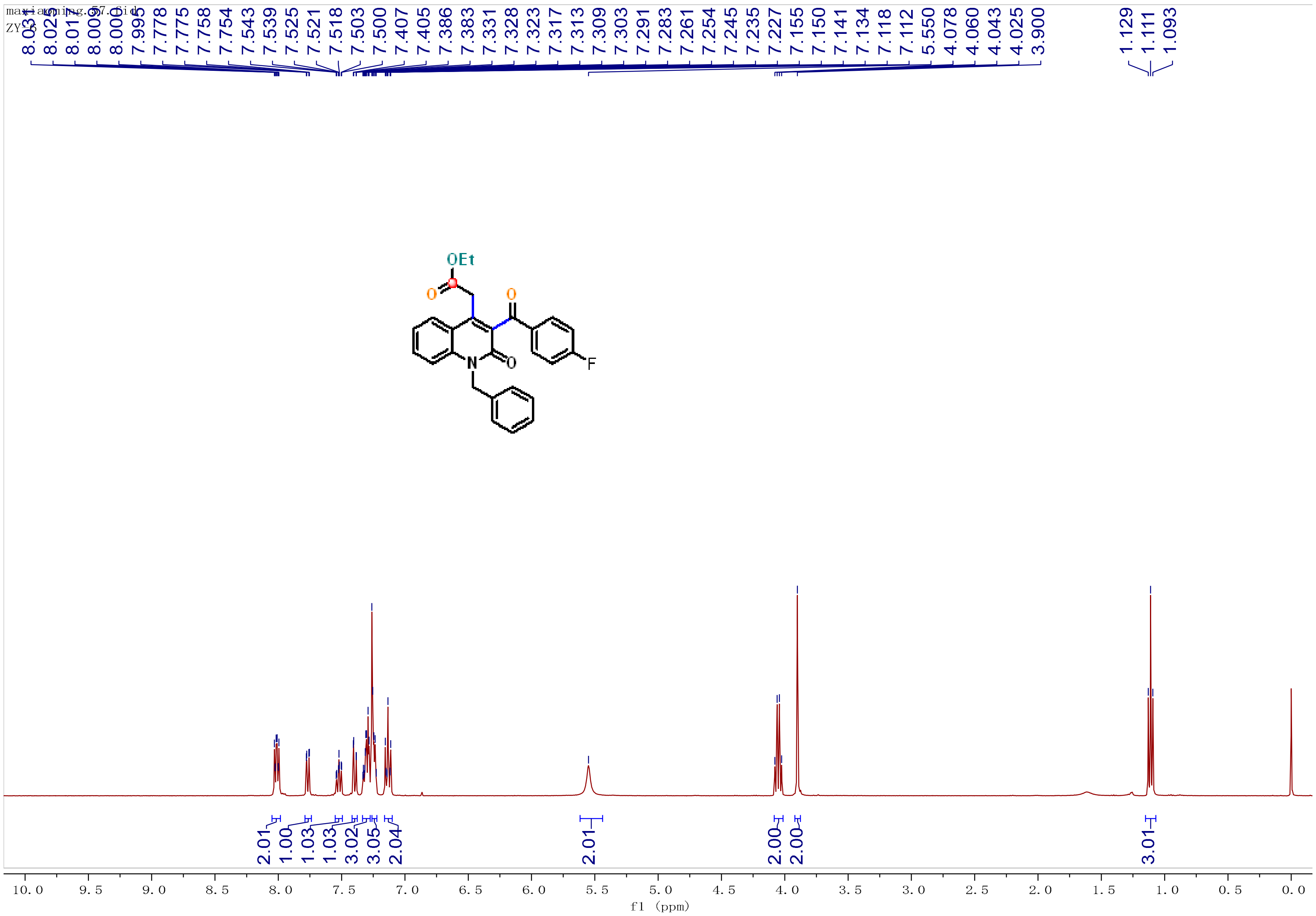

¹H NMR Spectrum of Compound 2f

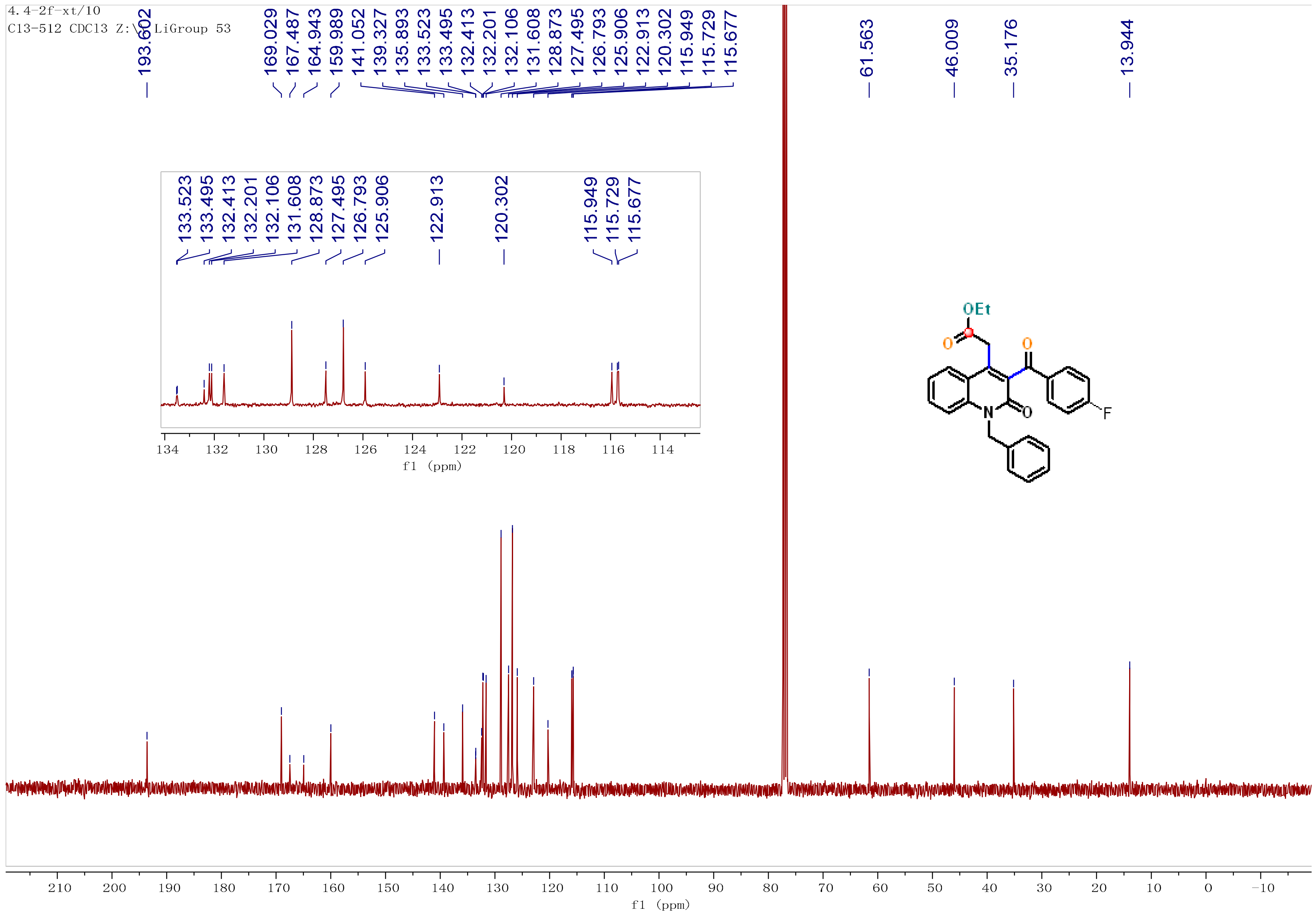

^{13}C NMR Spectrum of Compound 2f

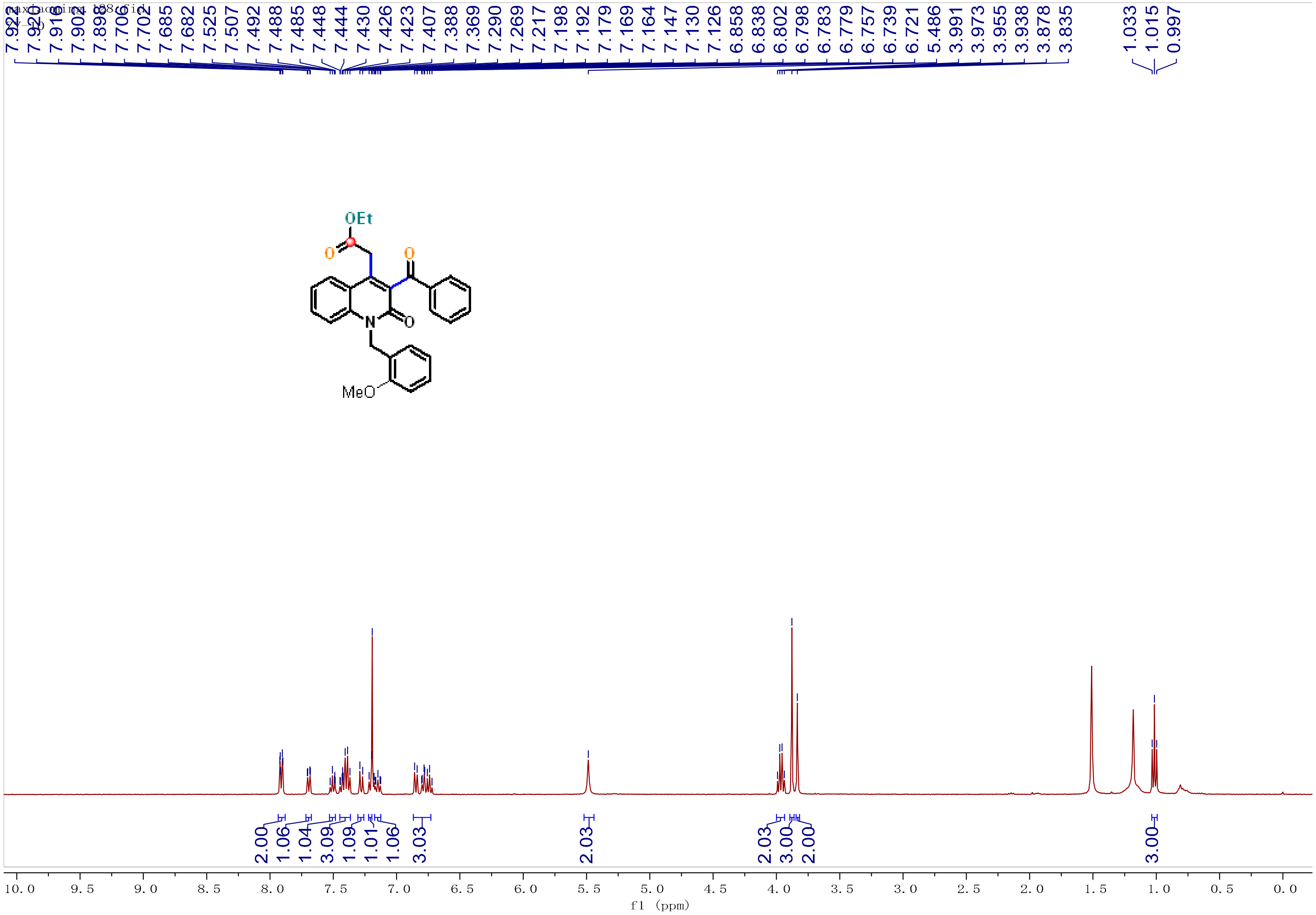

^{1}H NMR Spectrum of Compound 2g

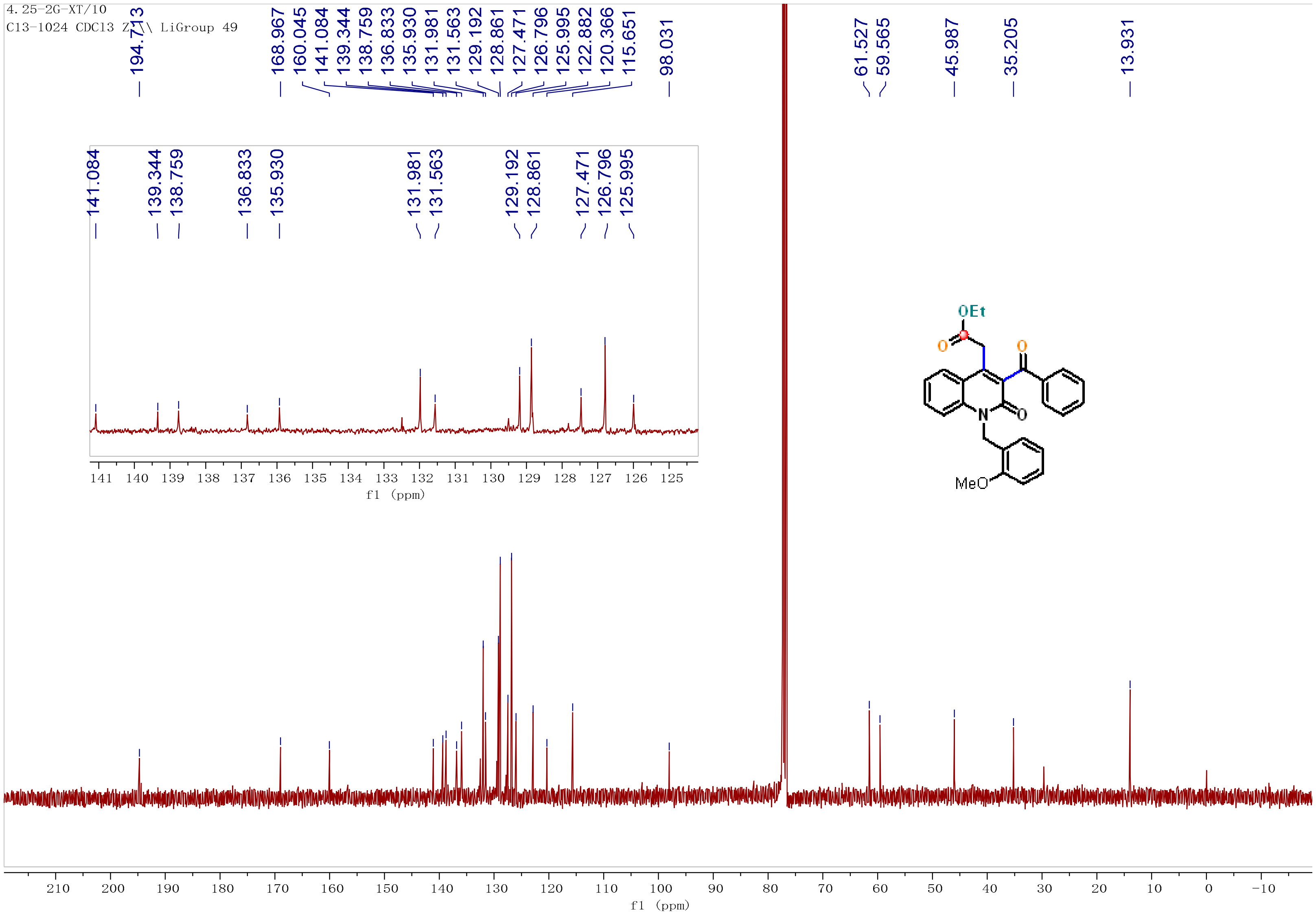

^{13}C NMR Spectrum of Compound 2g

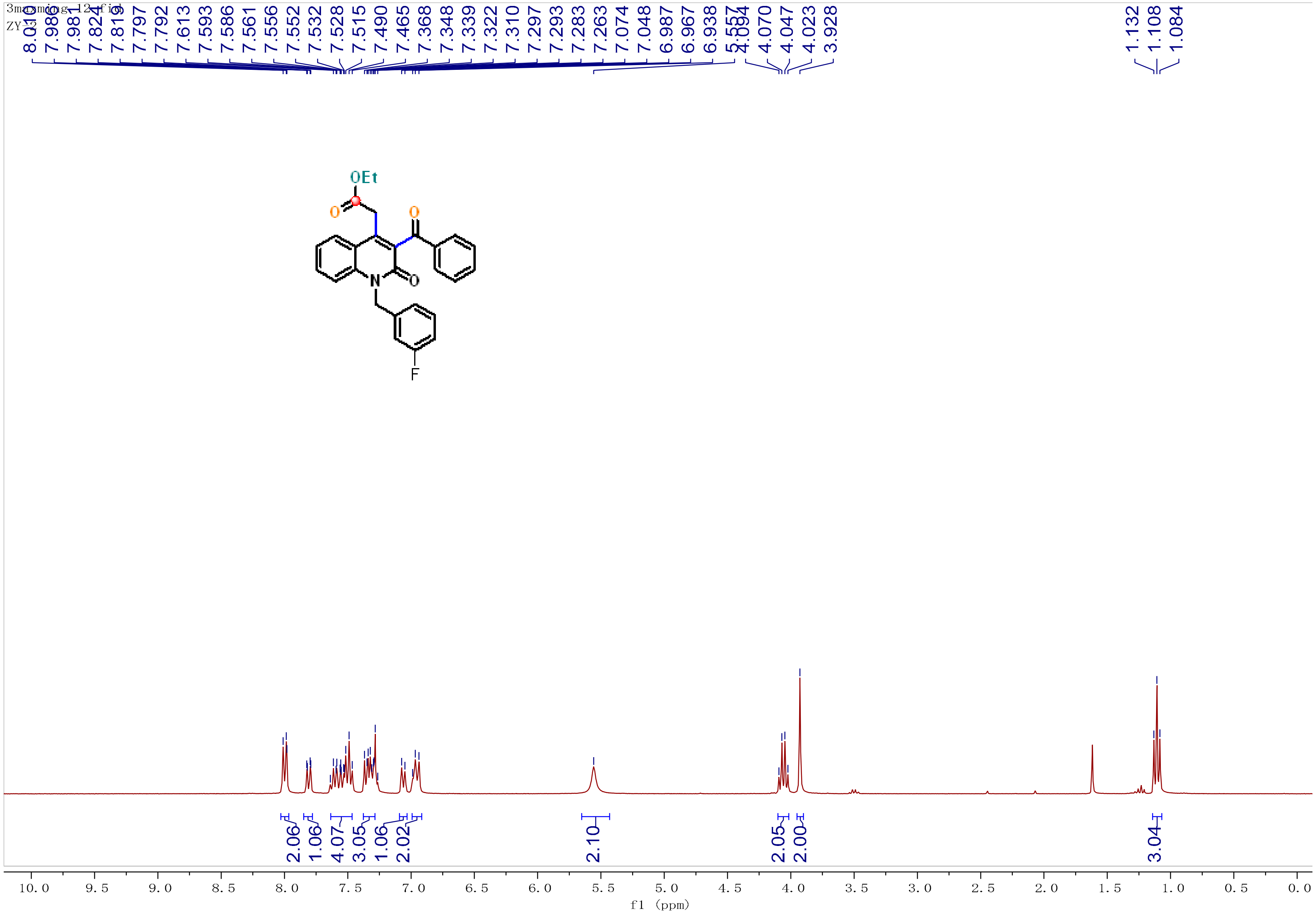

^{1}H NMR Spectrum of Compound 2h

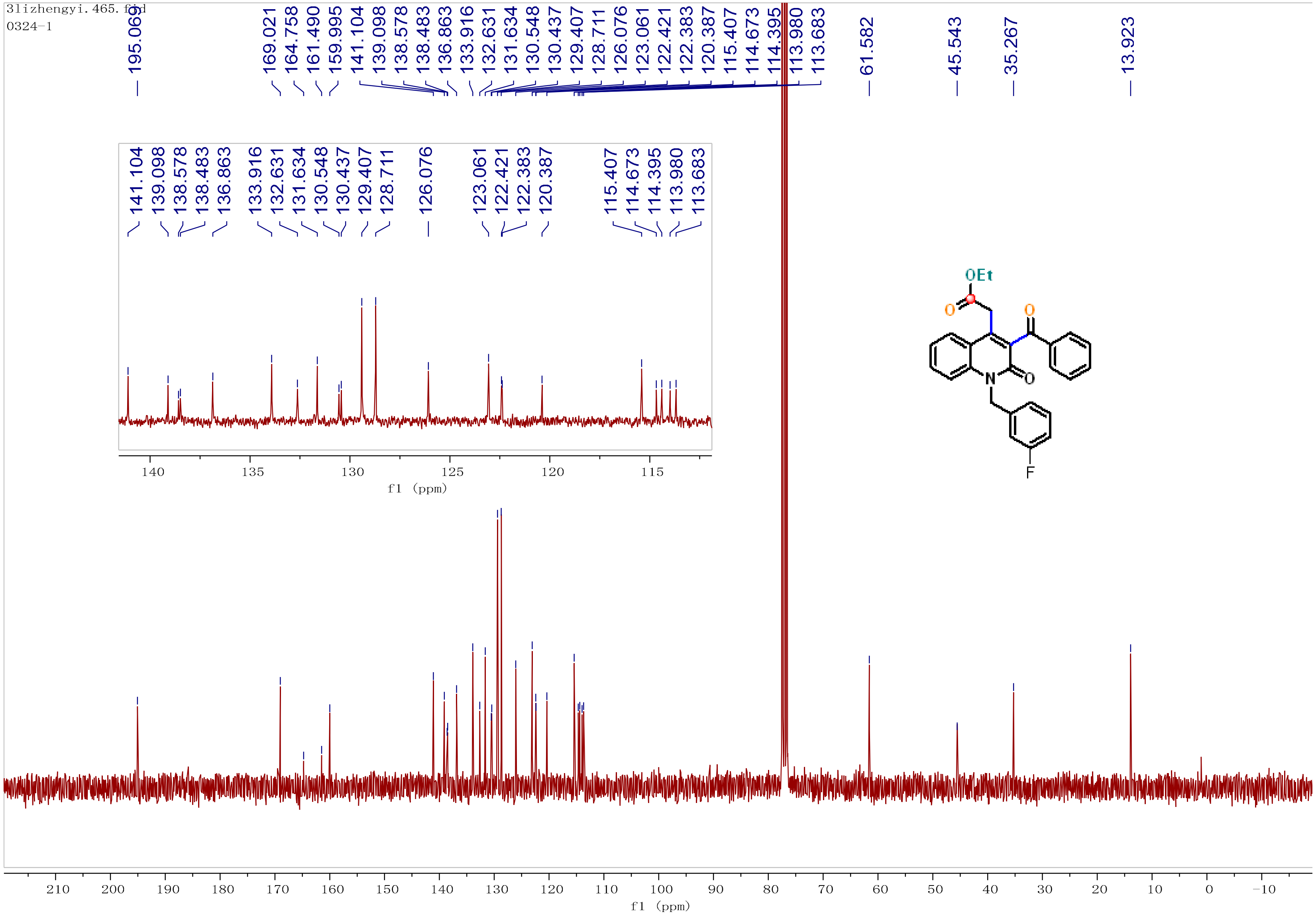

^{13}C NMR Spectrum of Compound 2h

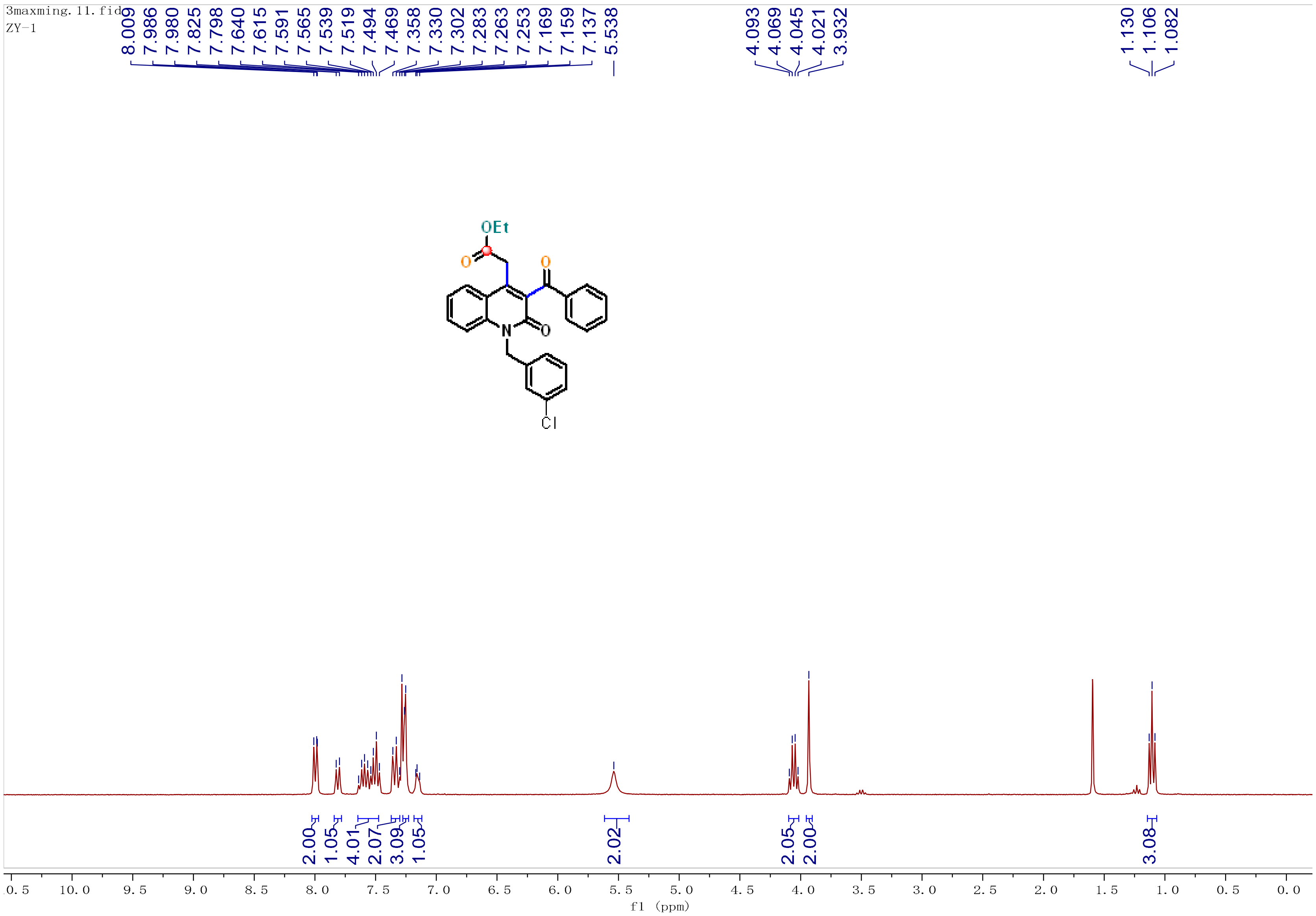

¹H NMR Spectrum of Compound 2i

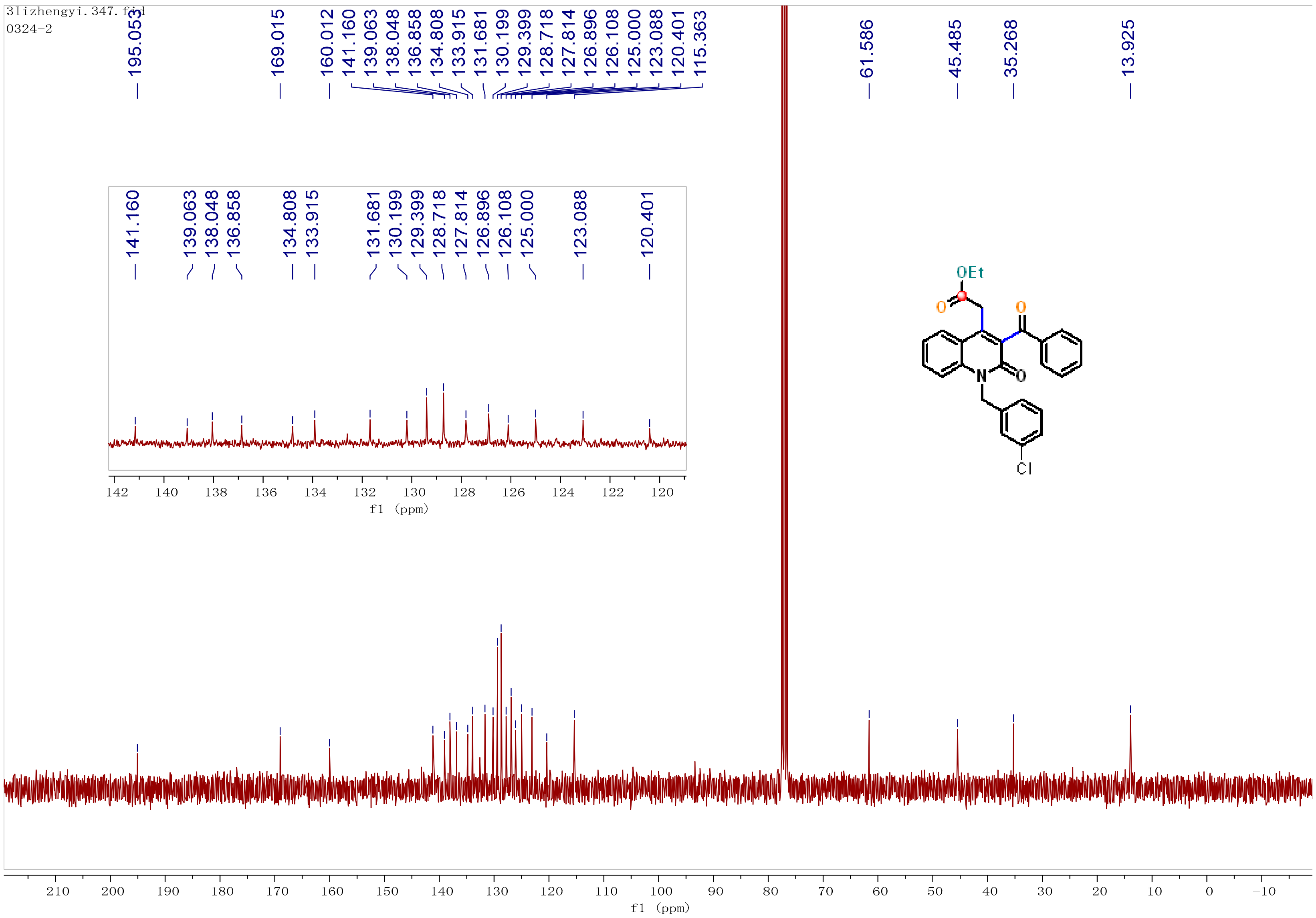

^{13}C NMR Spectrum of Compound 2i

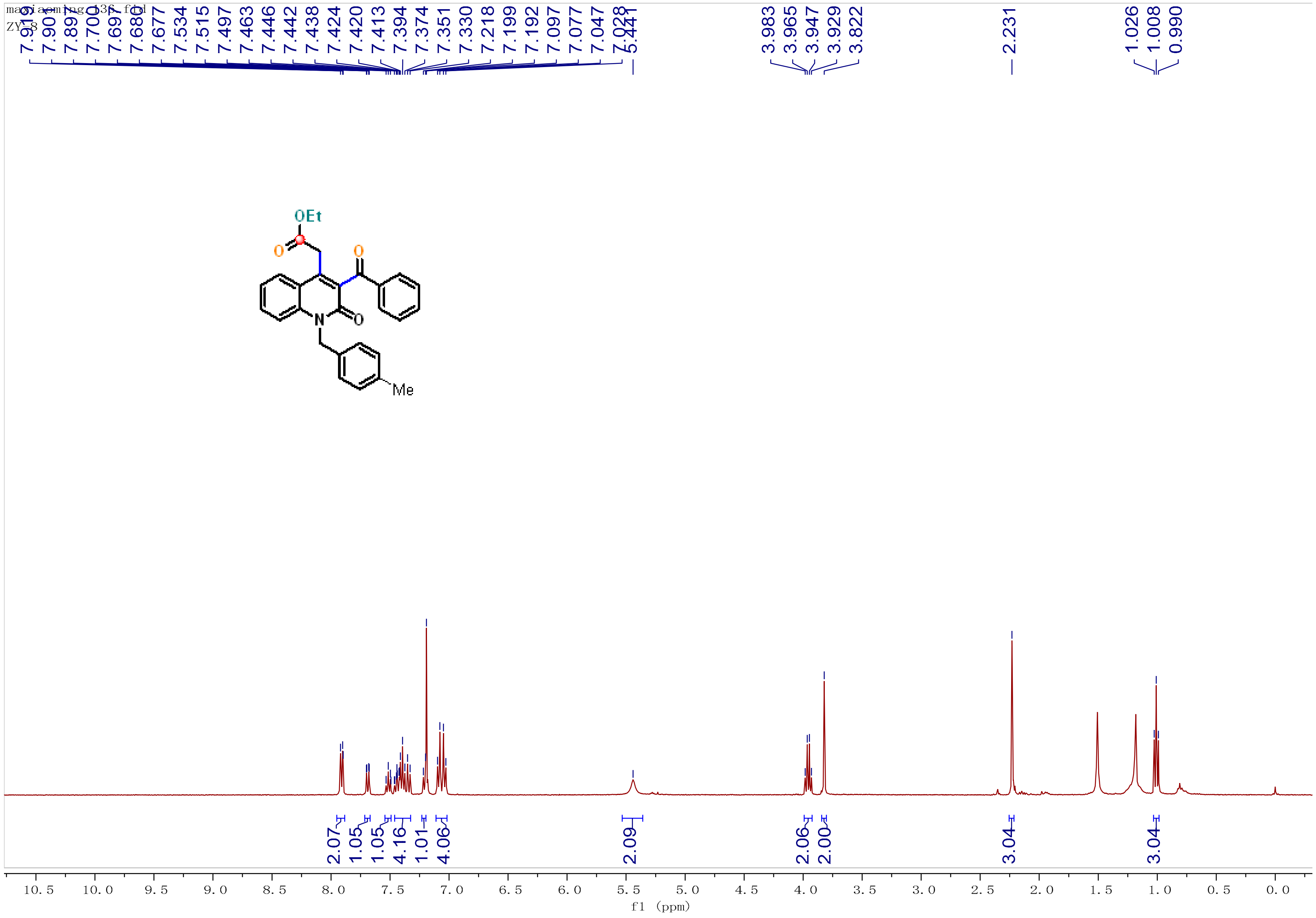

¹H NMR Spectrum of Compound 2j

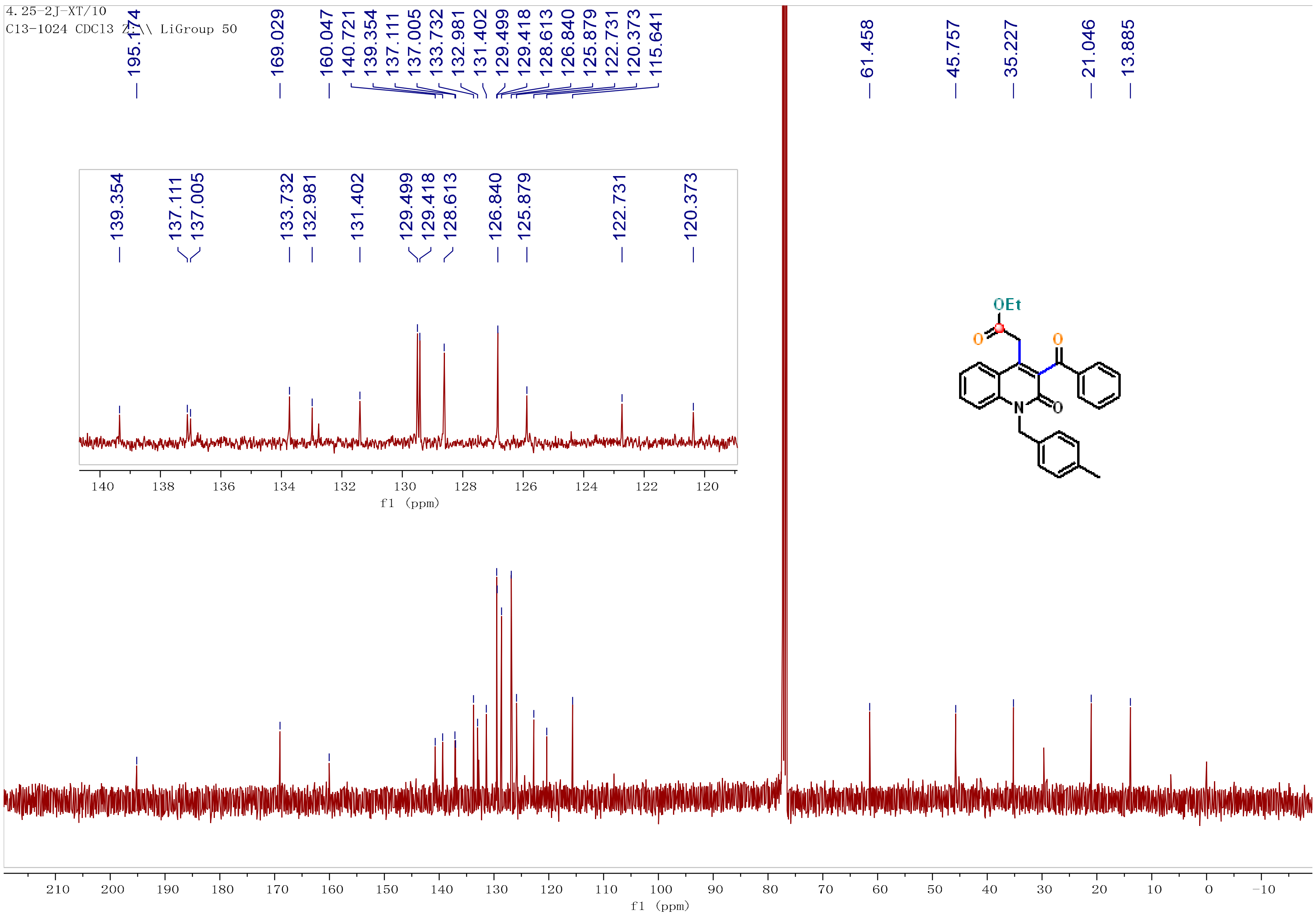

^{13}C NMR Spectrum of Compound 2j

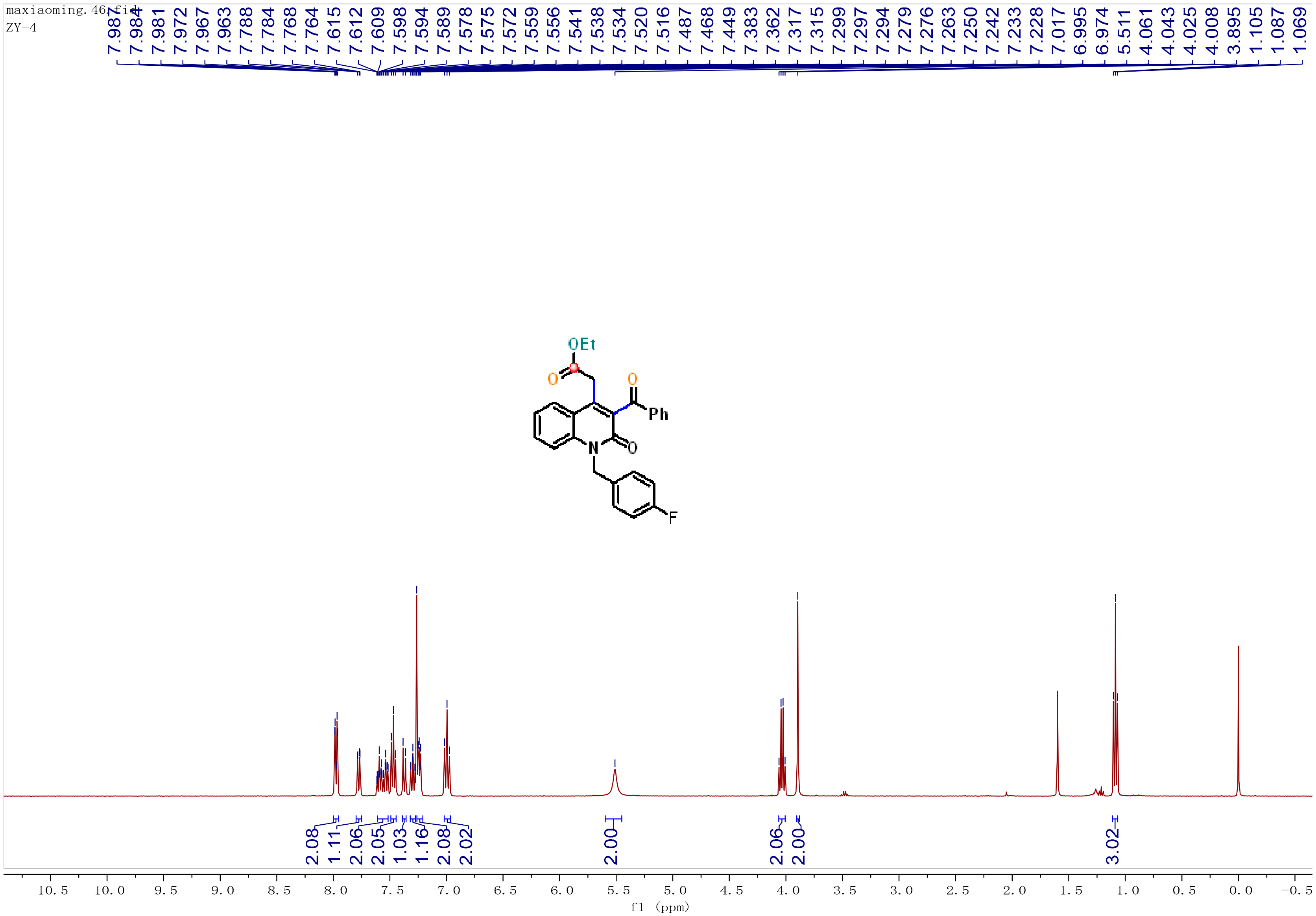

¹H NMR Spectrum of Compound 2k

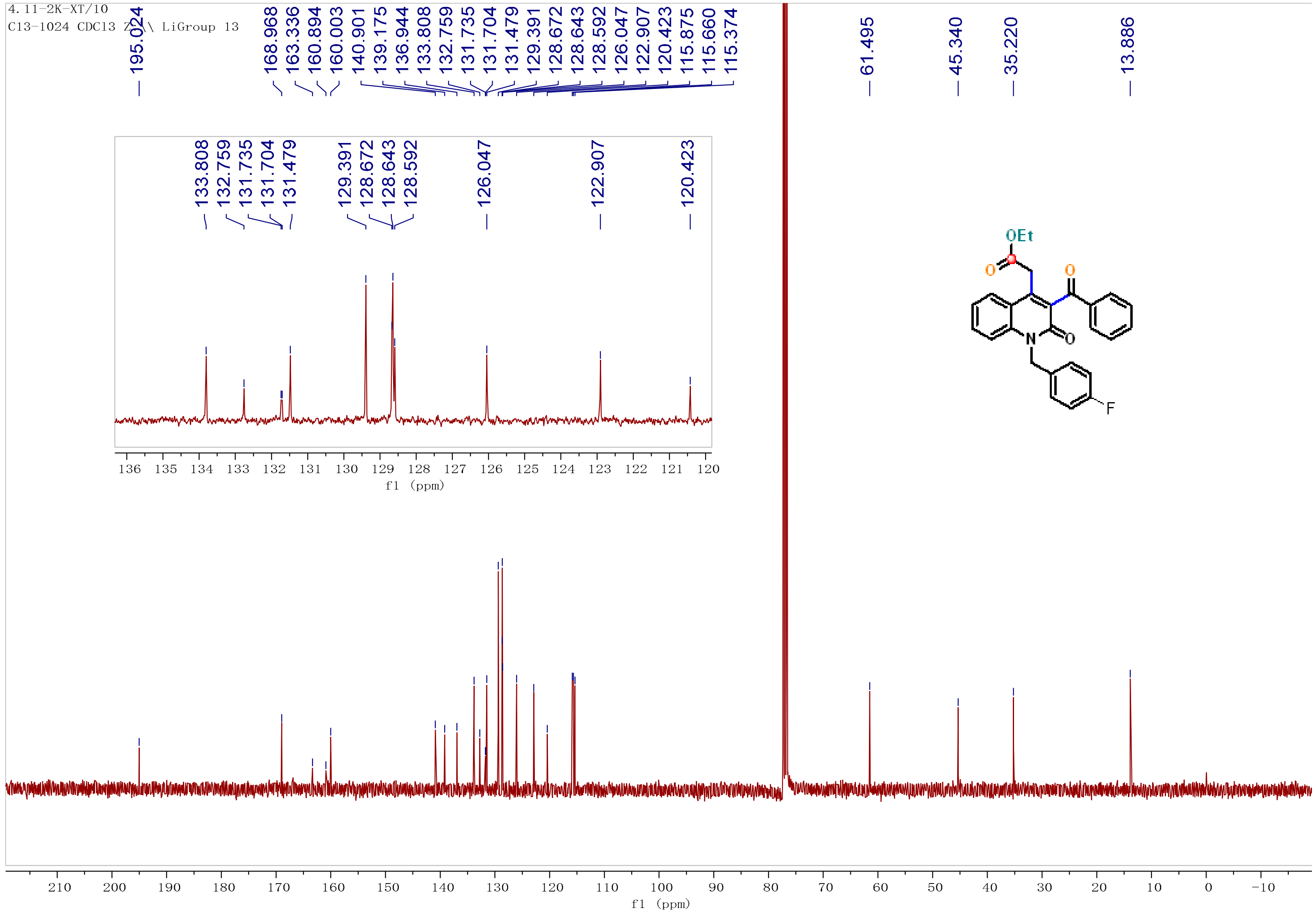

^{13}C NMR Spectrum of Compound 2k

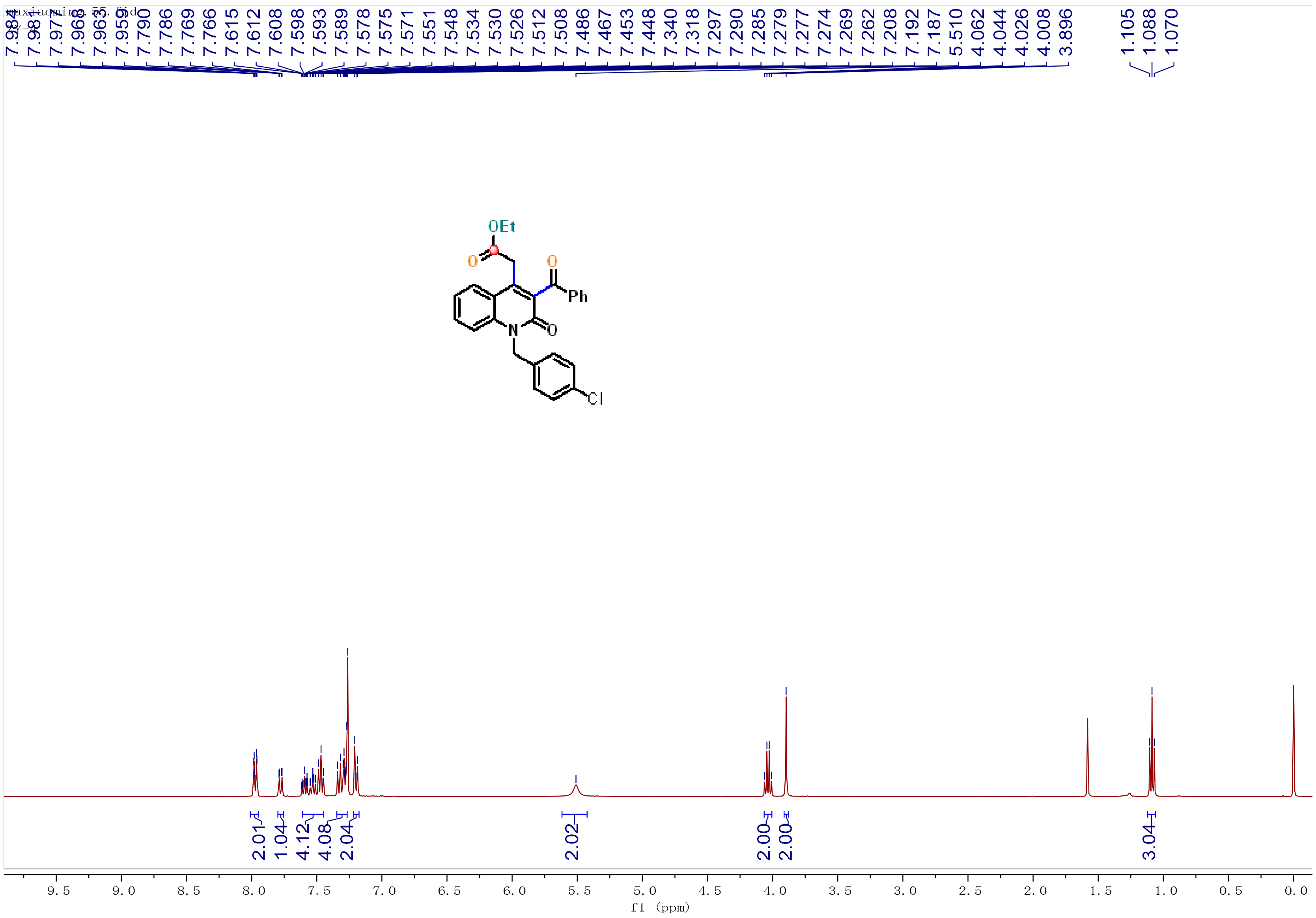

¹H NMR Spectrum of Compound 2l

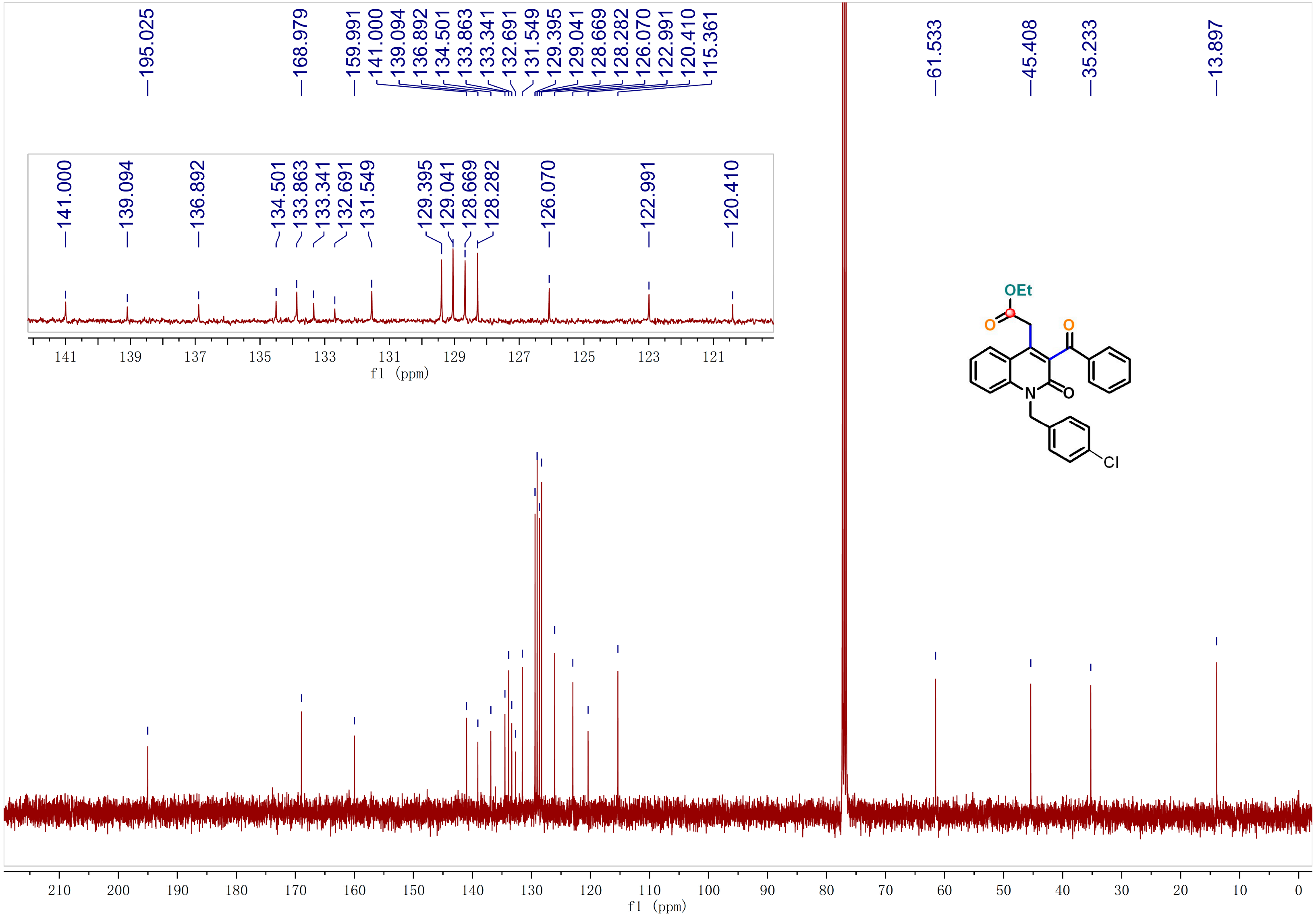

¹³C NMR Spectrum of Compound 2l

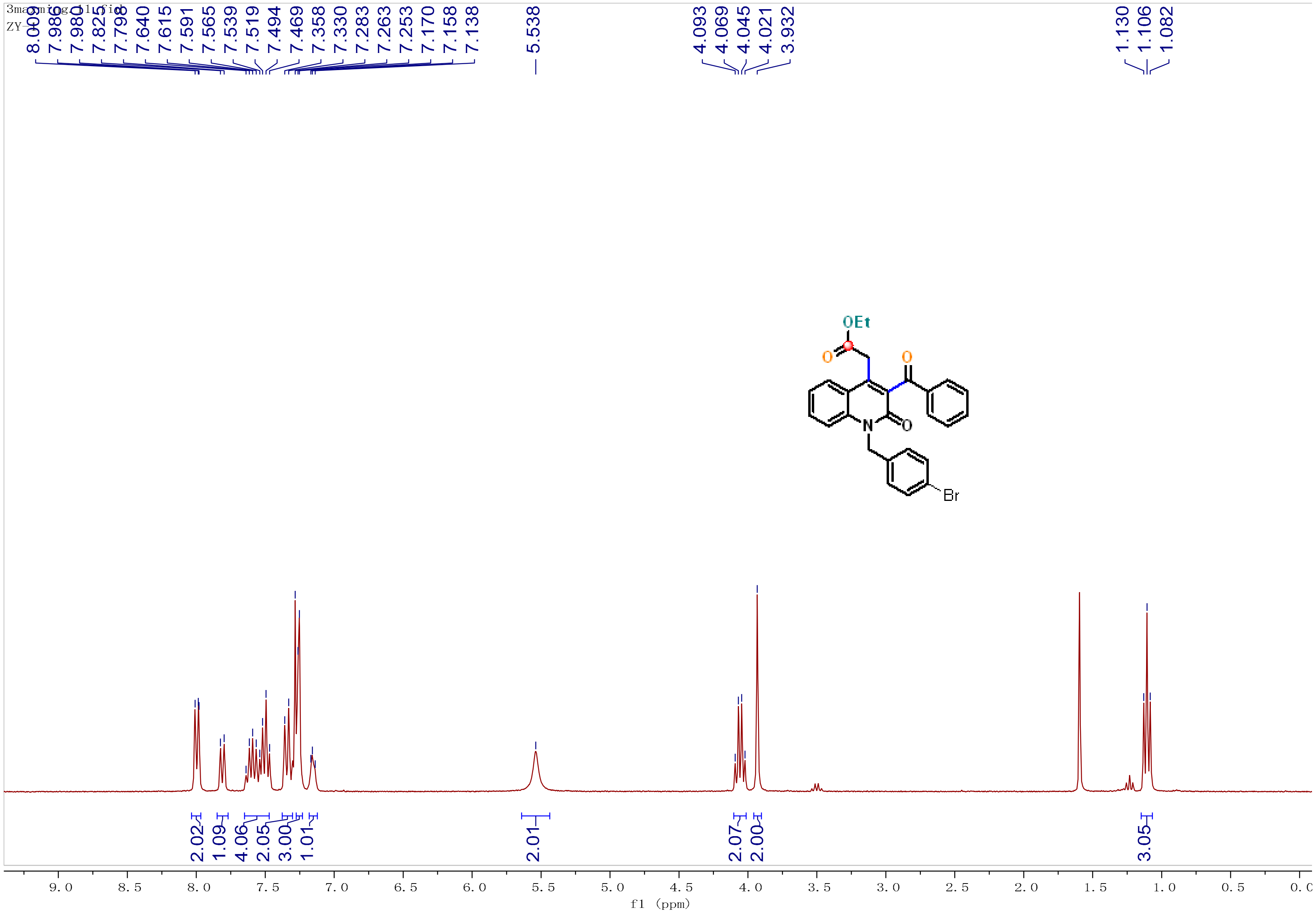

¹H NMR Spectrum of Compound 2m

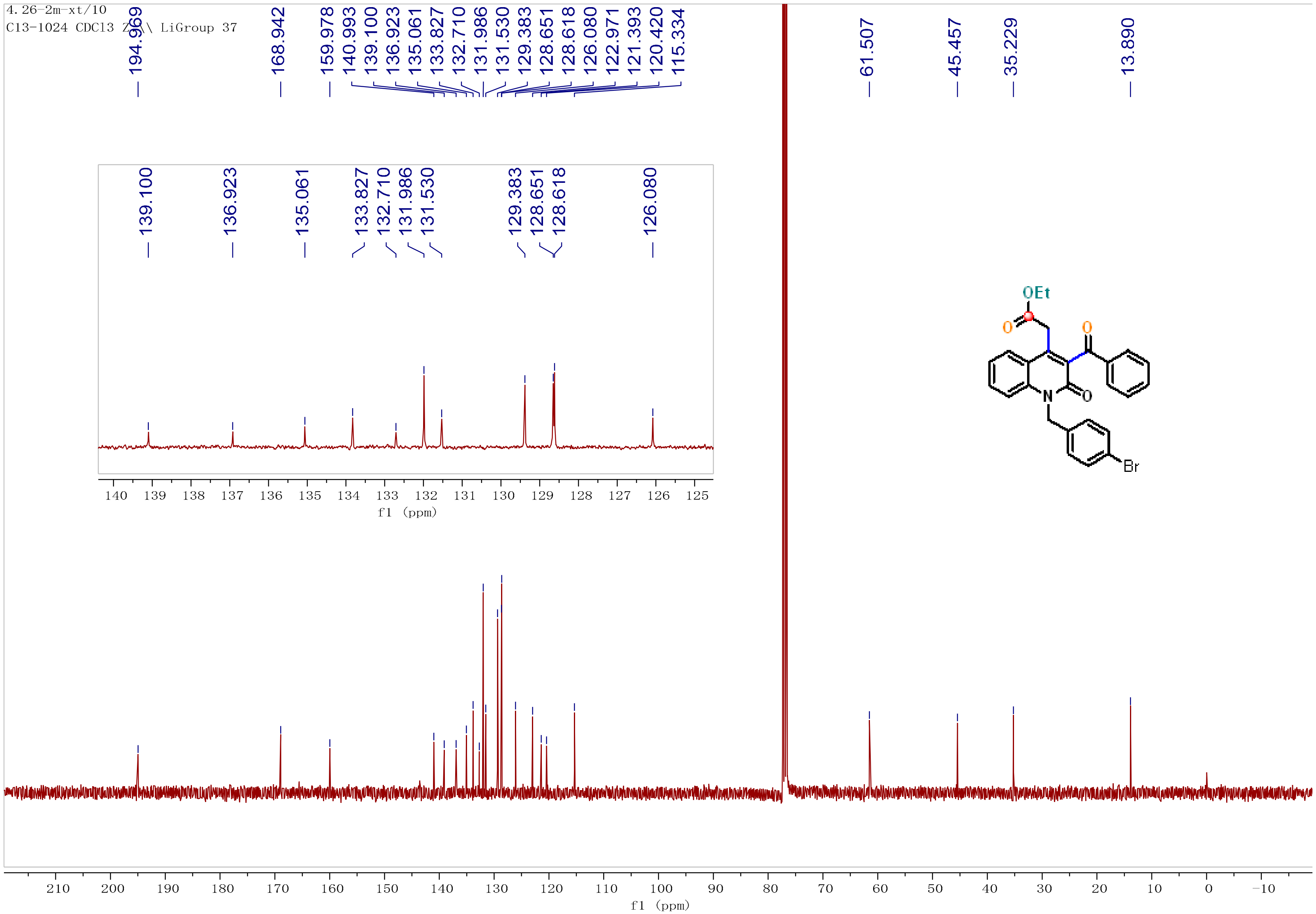

^{13}C NMR Spectrum of Compound 2m

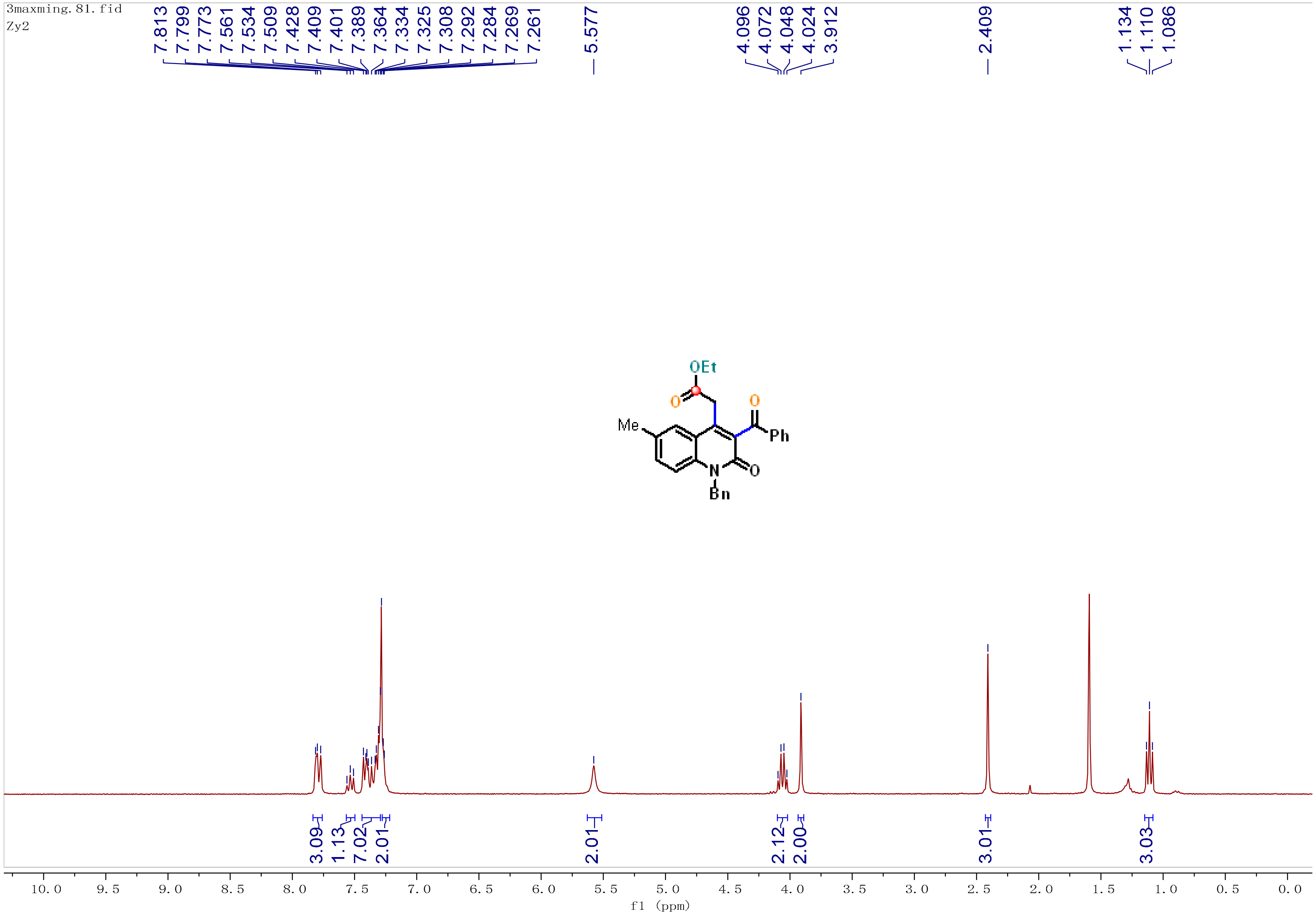

¹H NMR Spectrum of Compound 2n

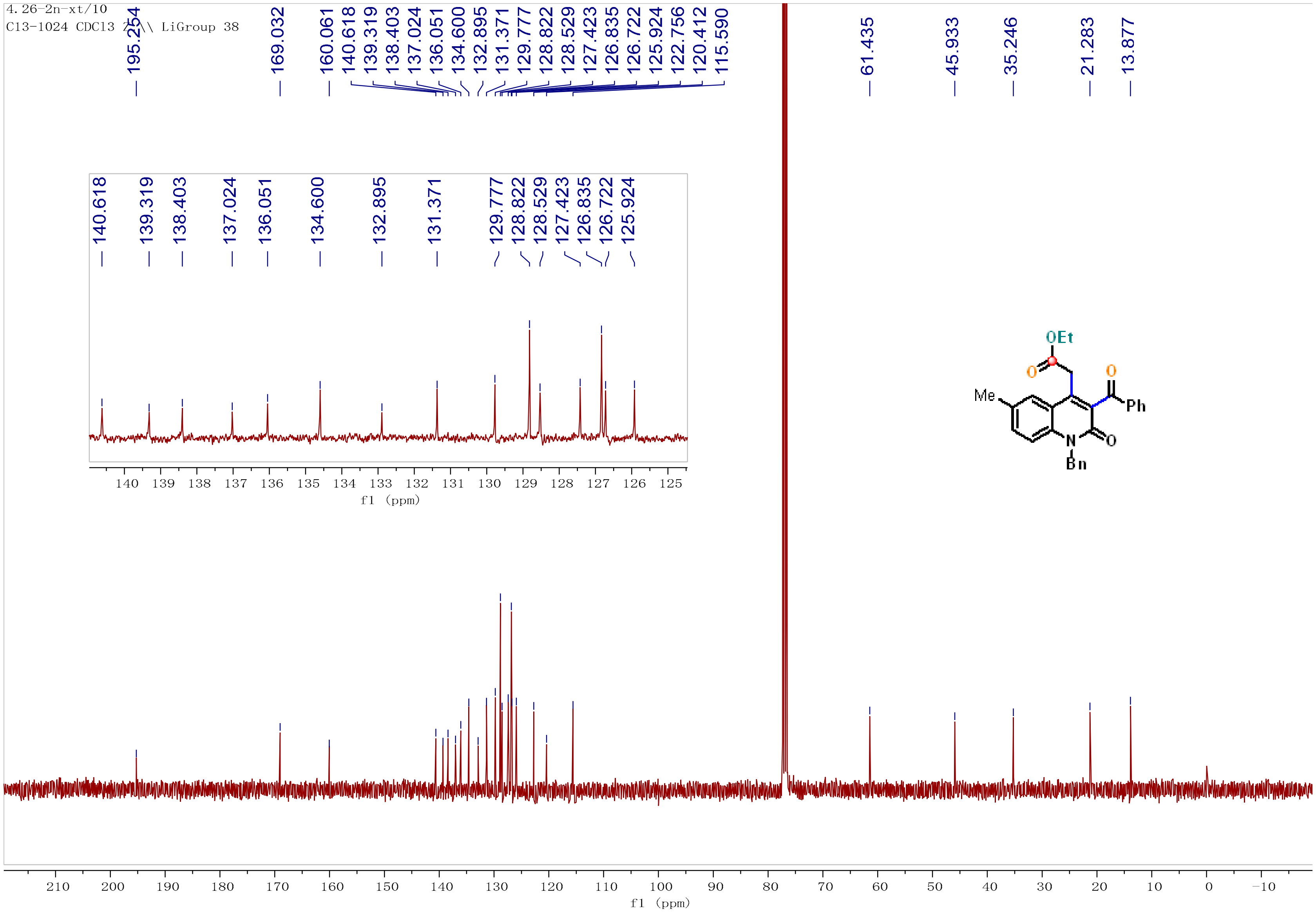

^{13}C NMR Spectrum of Compound 2n

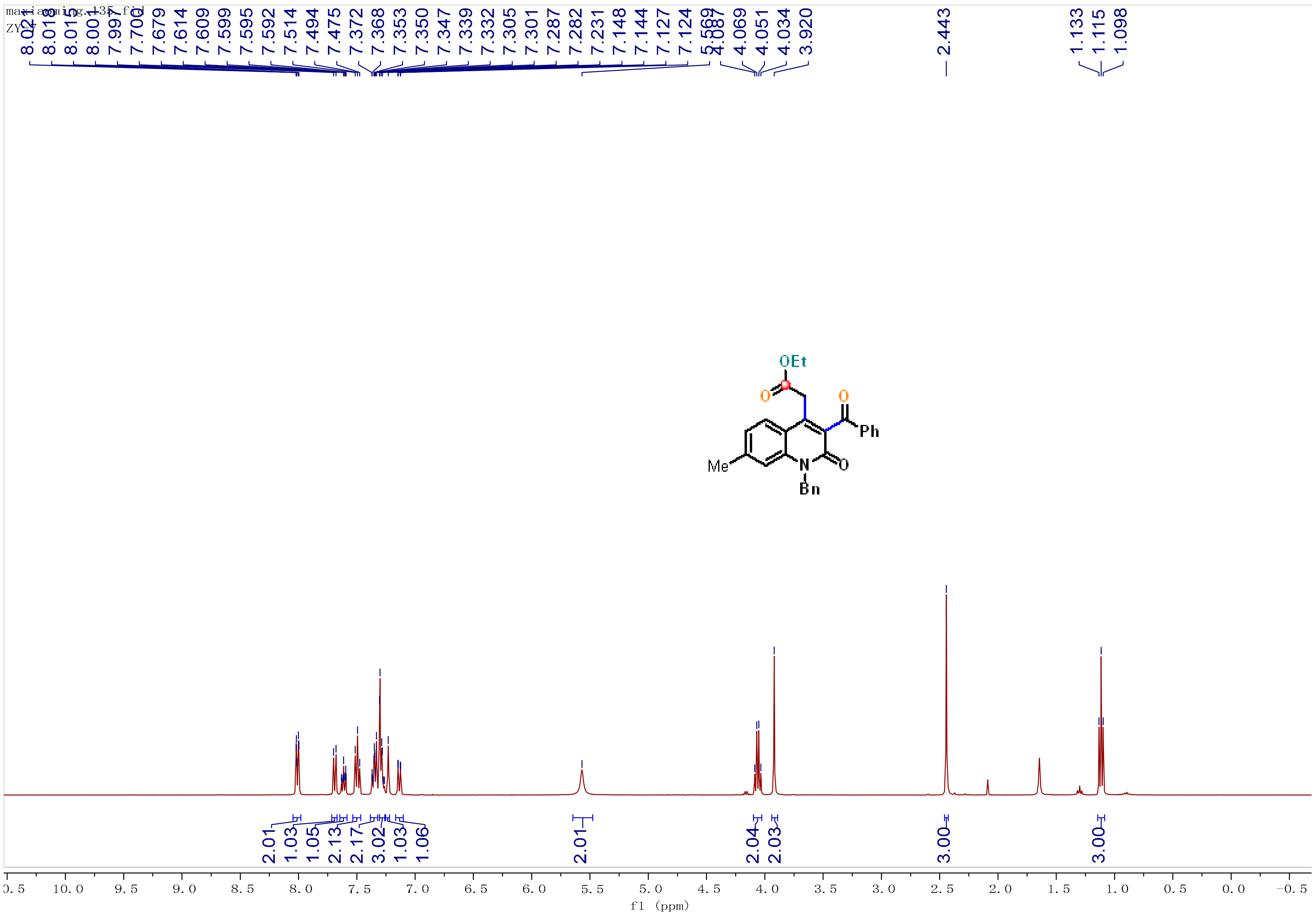

¹H NMR Spectrum of Compound 2o

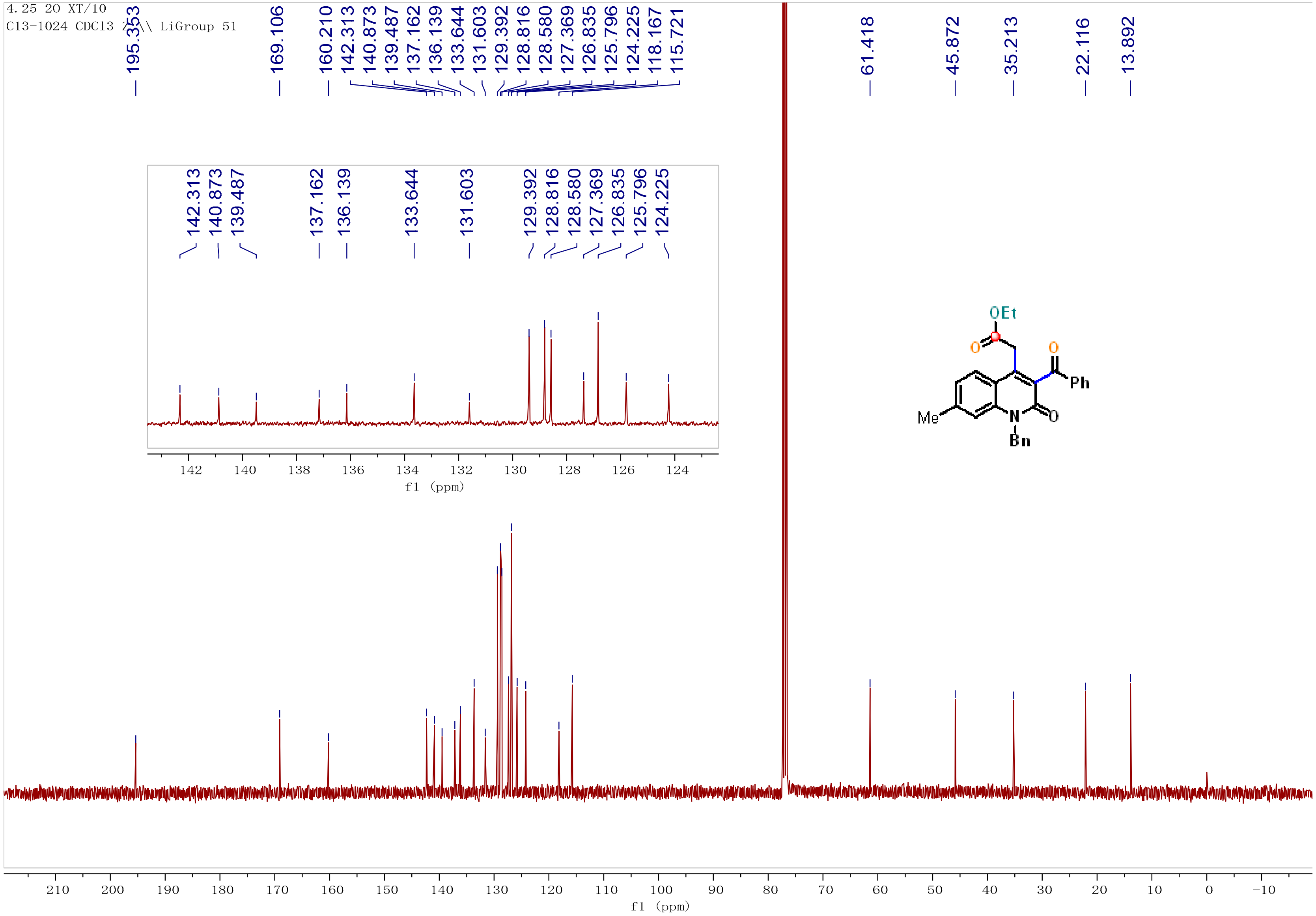

^{13}C NMR Spectrum of Compound 2o

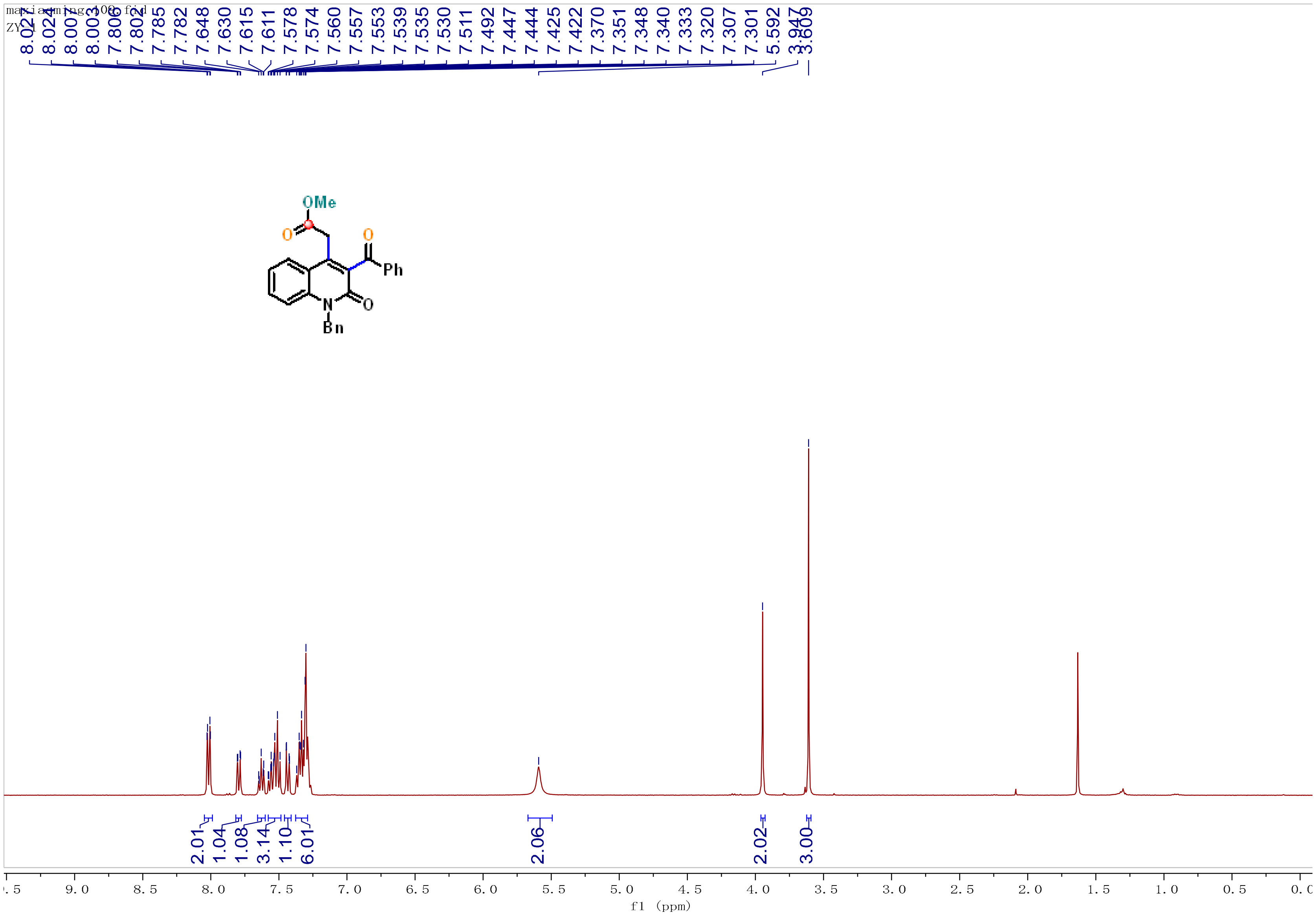

¹H NMR Spectrum of Compound 2p

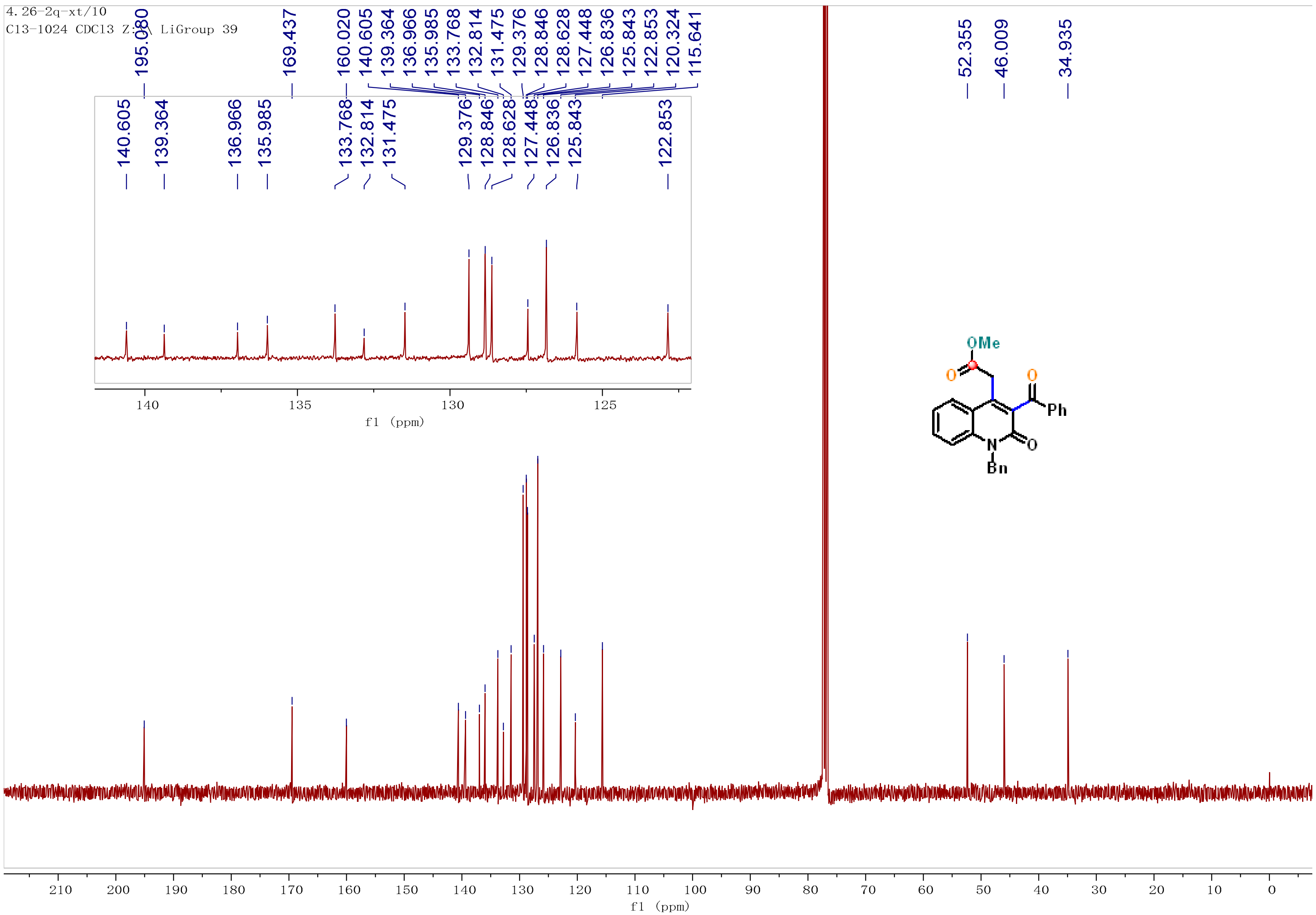

^{13}C NMR Spectrum of Compound 2p

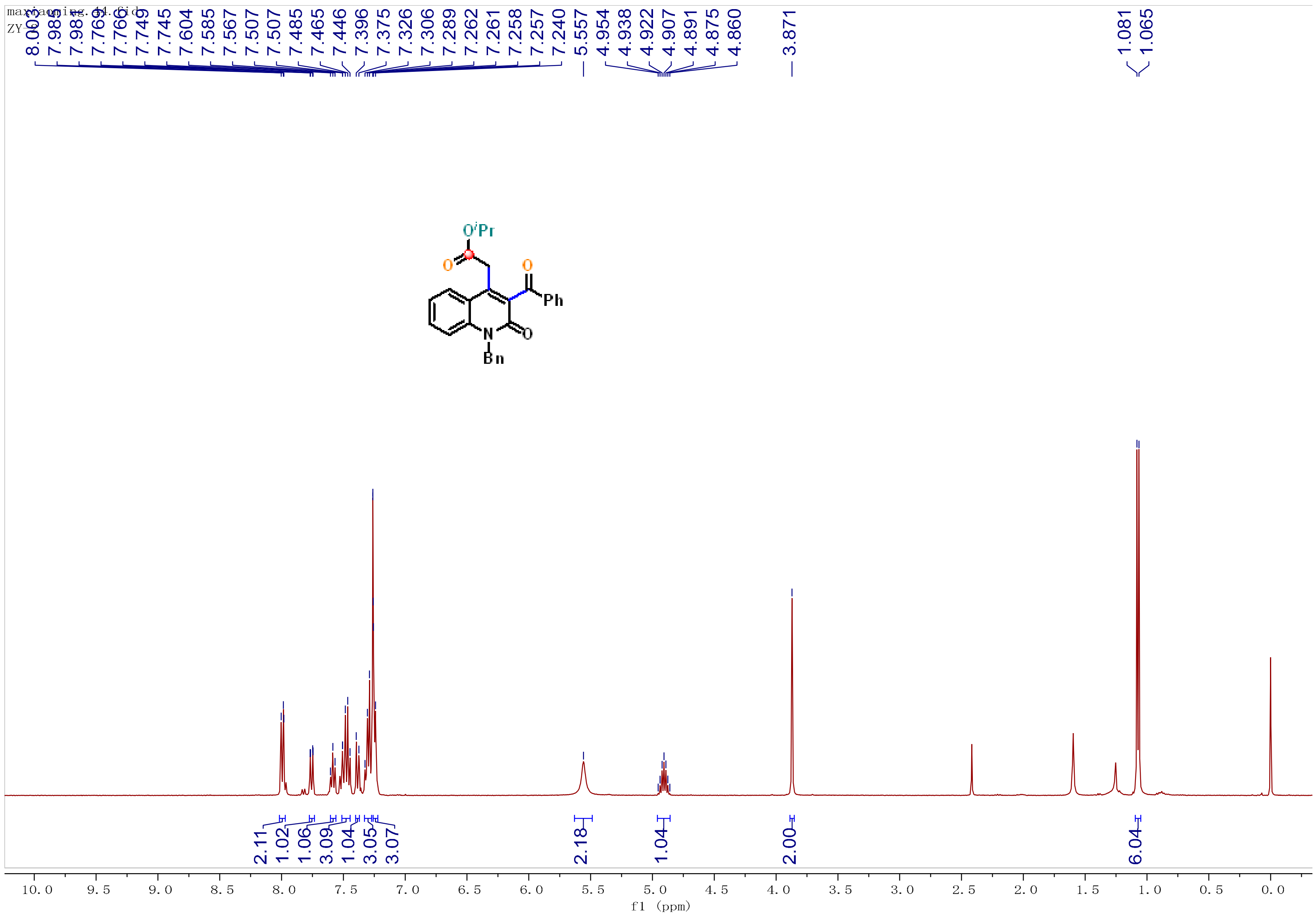

^{1}H NMR Spectrum of Compound 2q

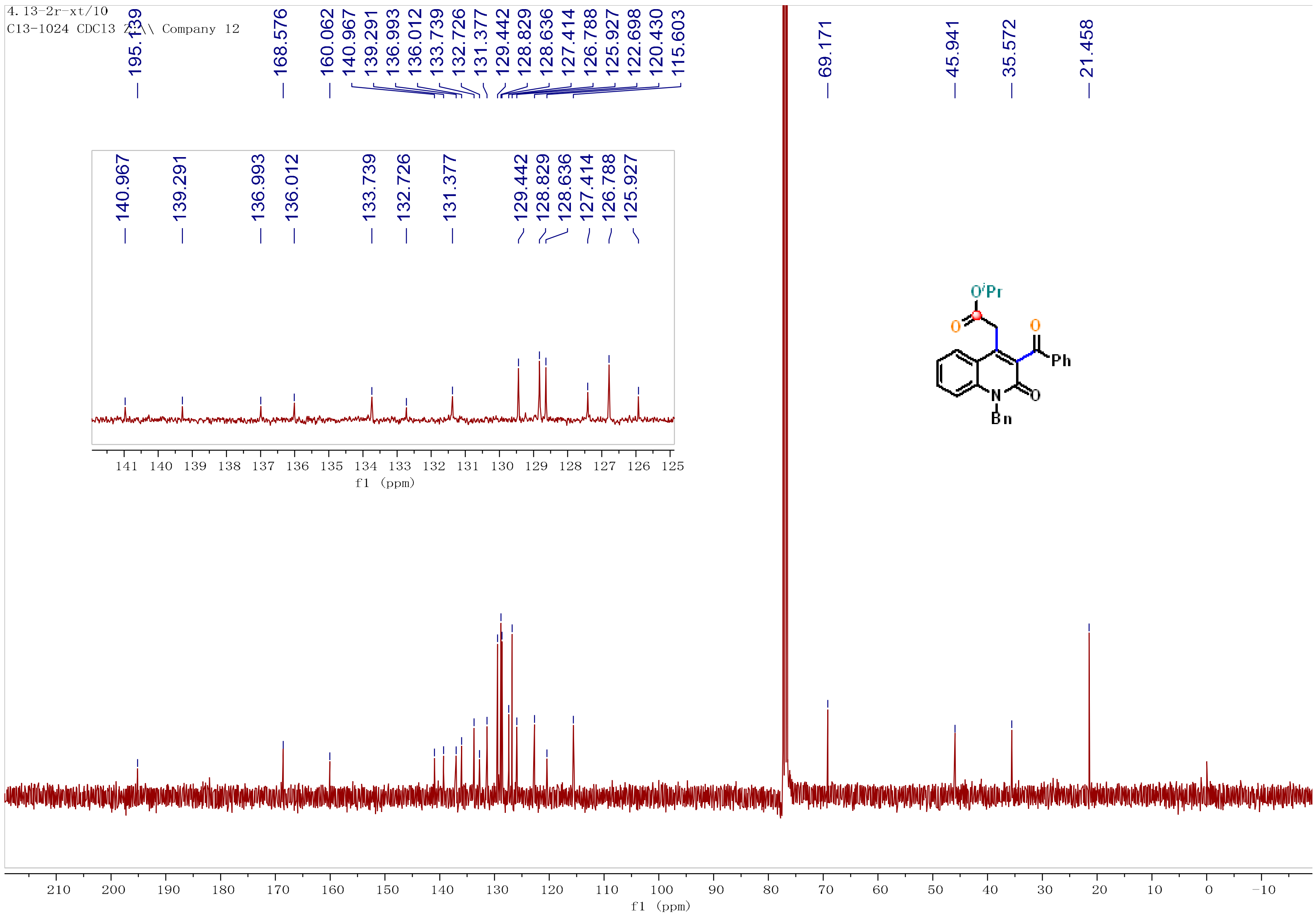

^{13}C NMR Spectrum of Compound 2q

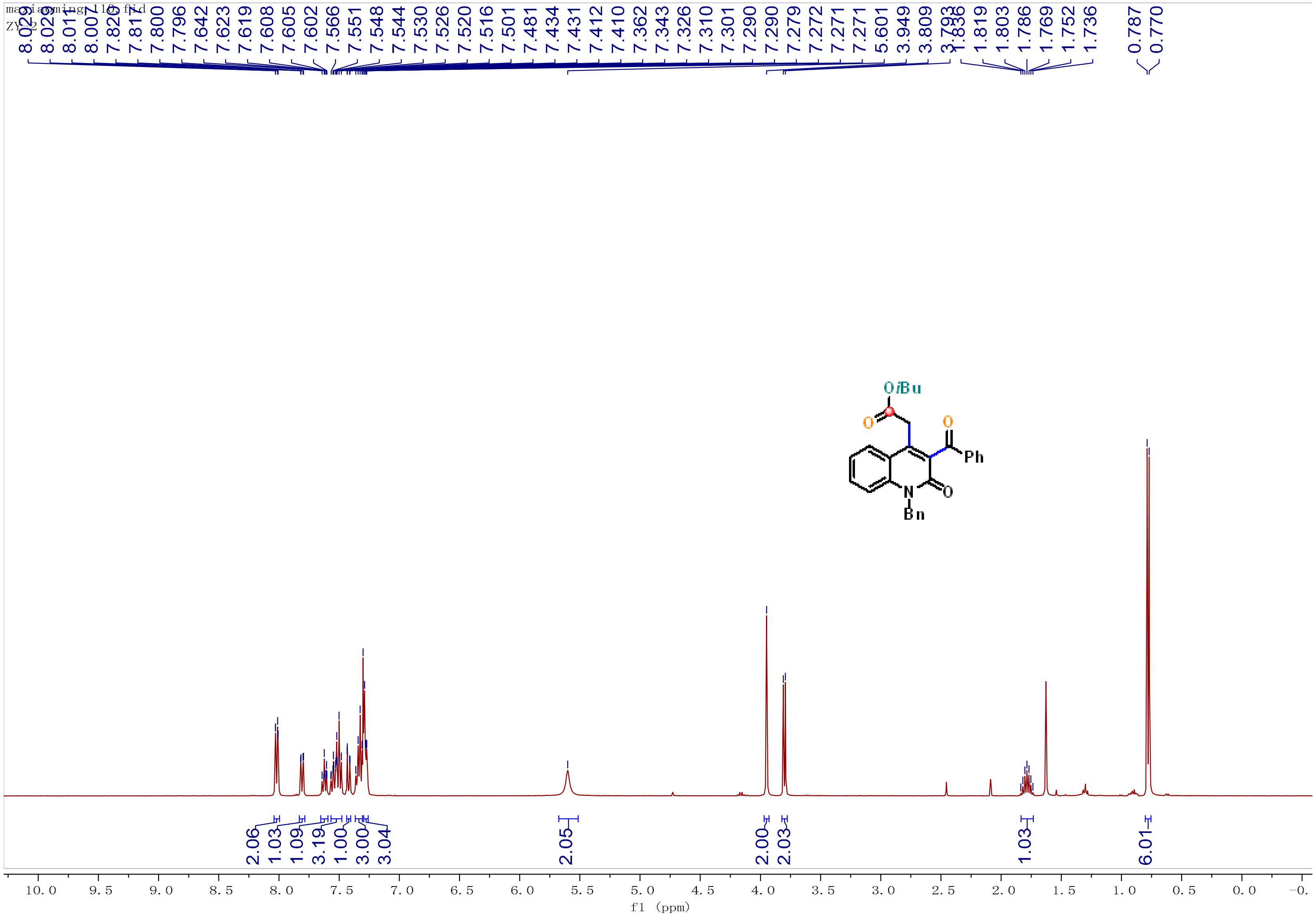

¹H NMR Spectrum of Compound 2r

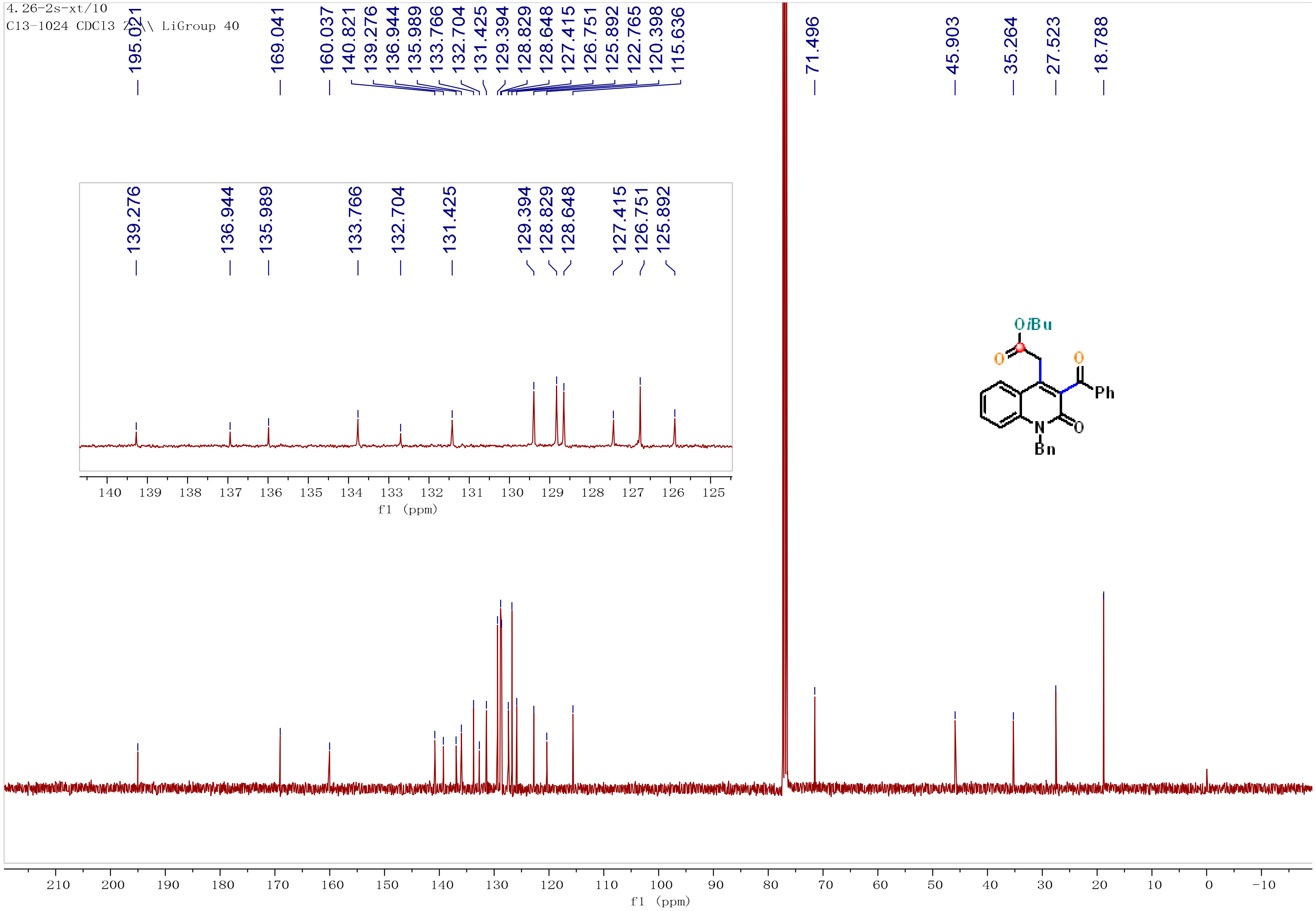

^{13}C NMR Spectrum of Compound 3r

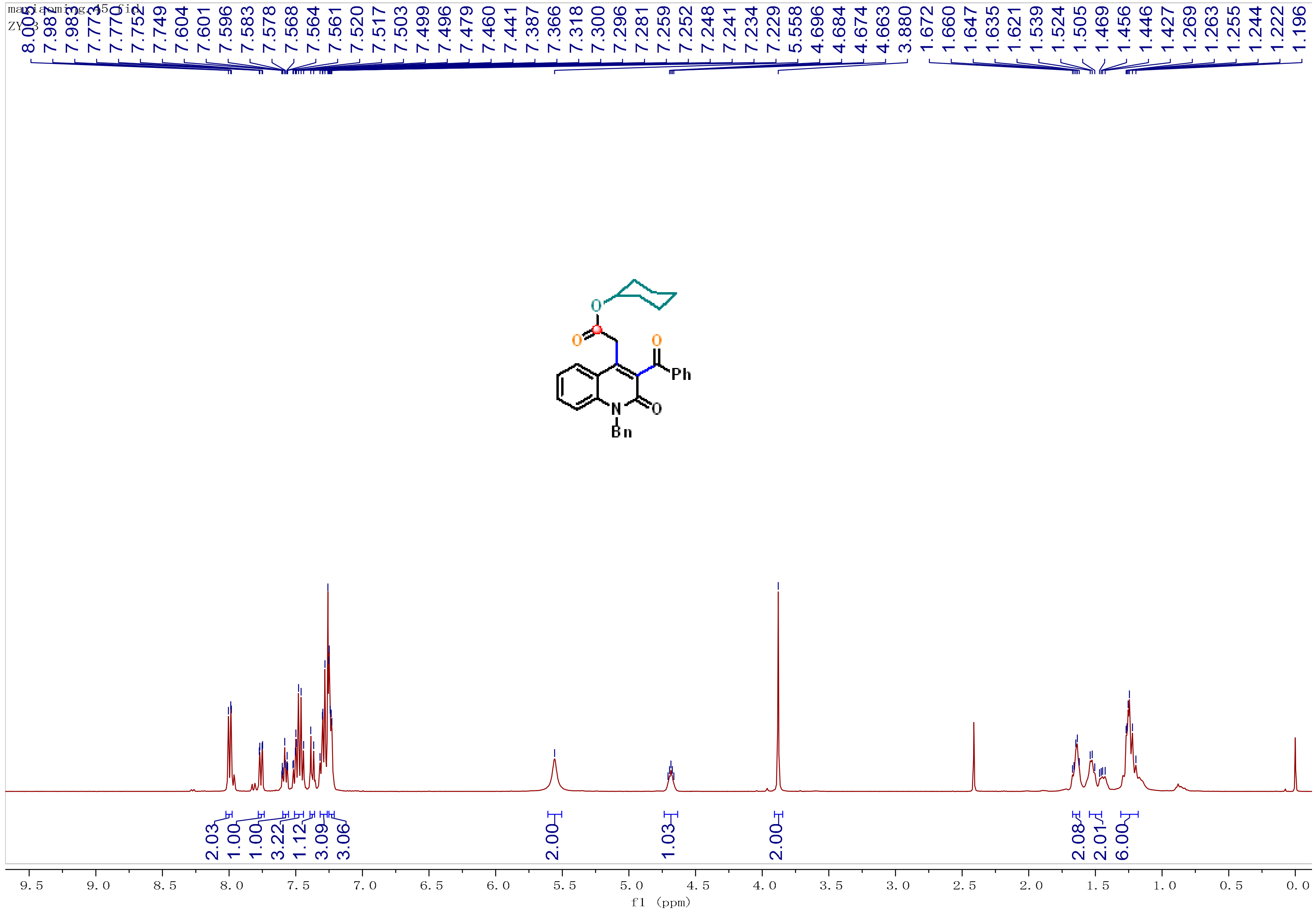

^{1}H NMR Spectrum of Compound 2s

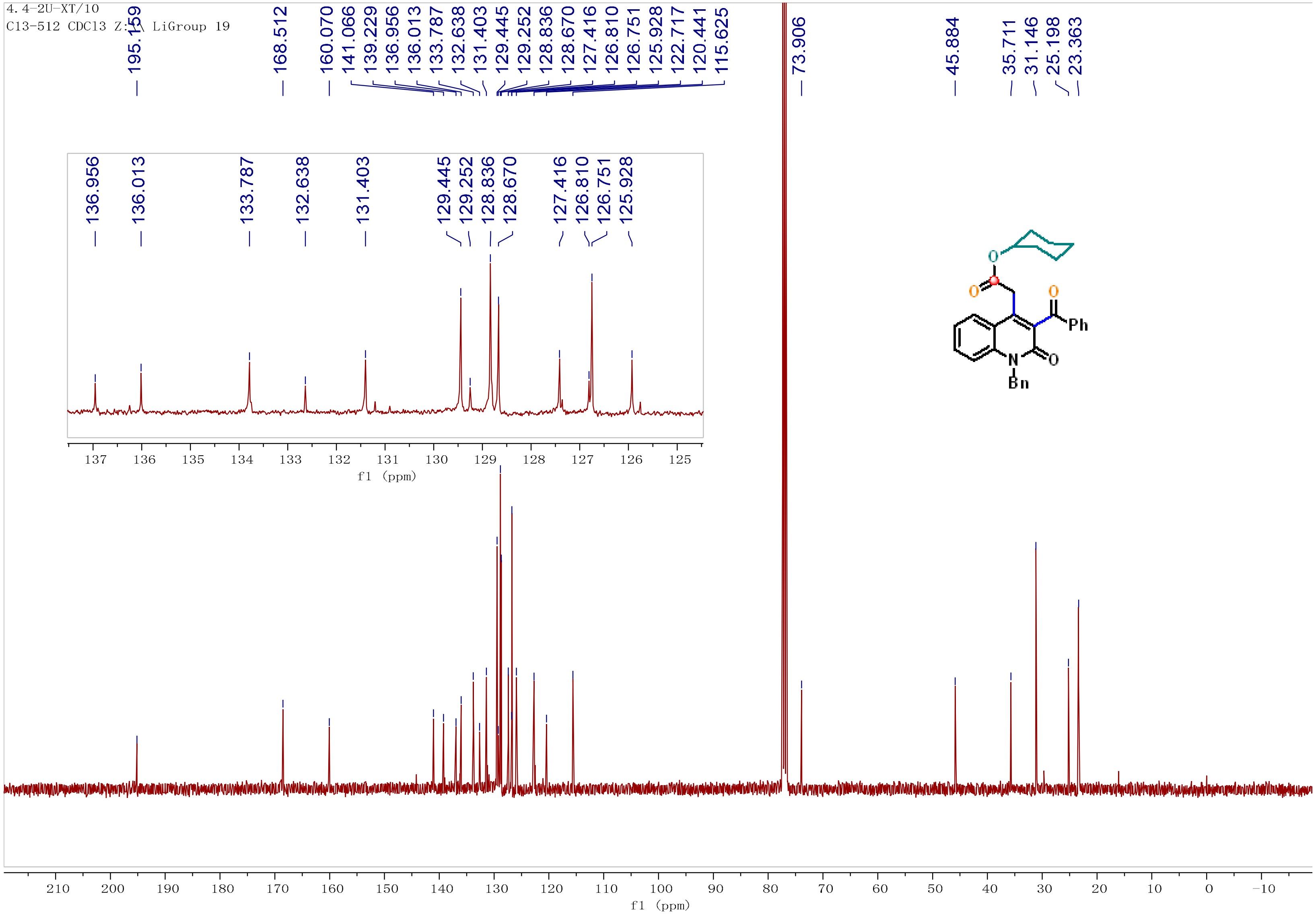

^{13}C NMR Spectrum of Compound 2s

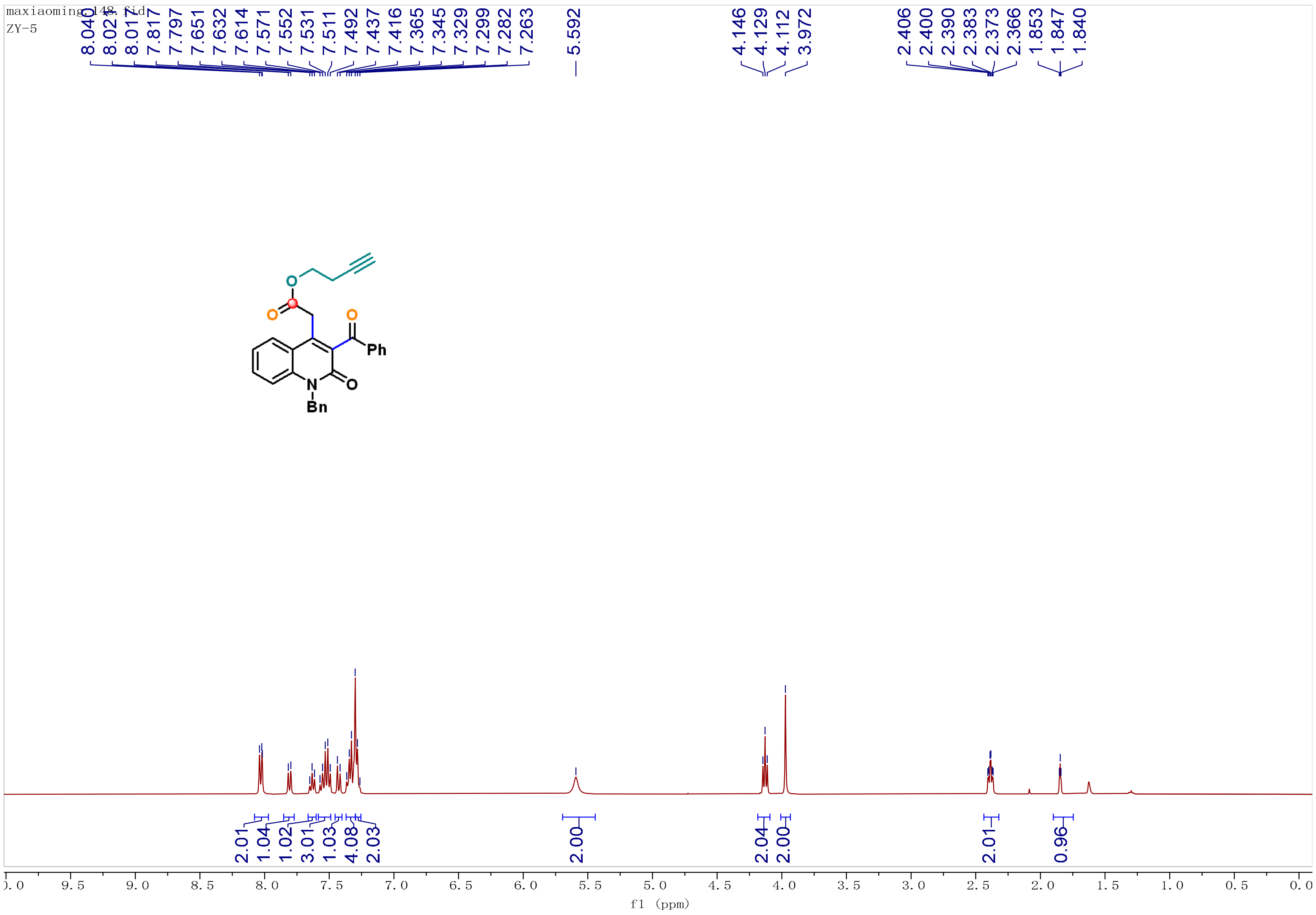

¹H NMR Spectrum of Compound 2t

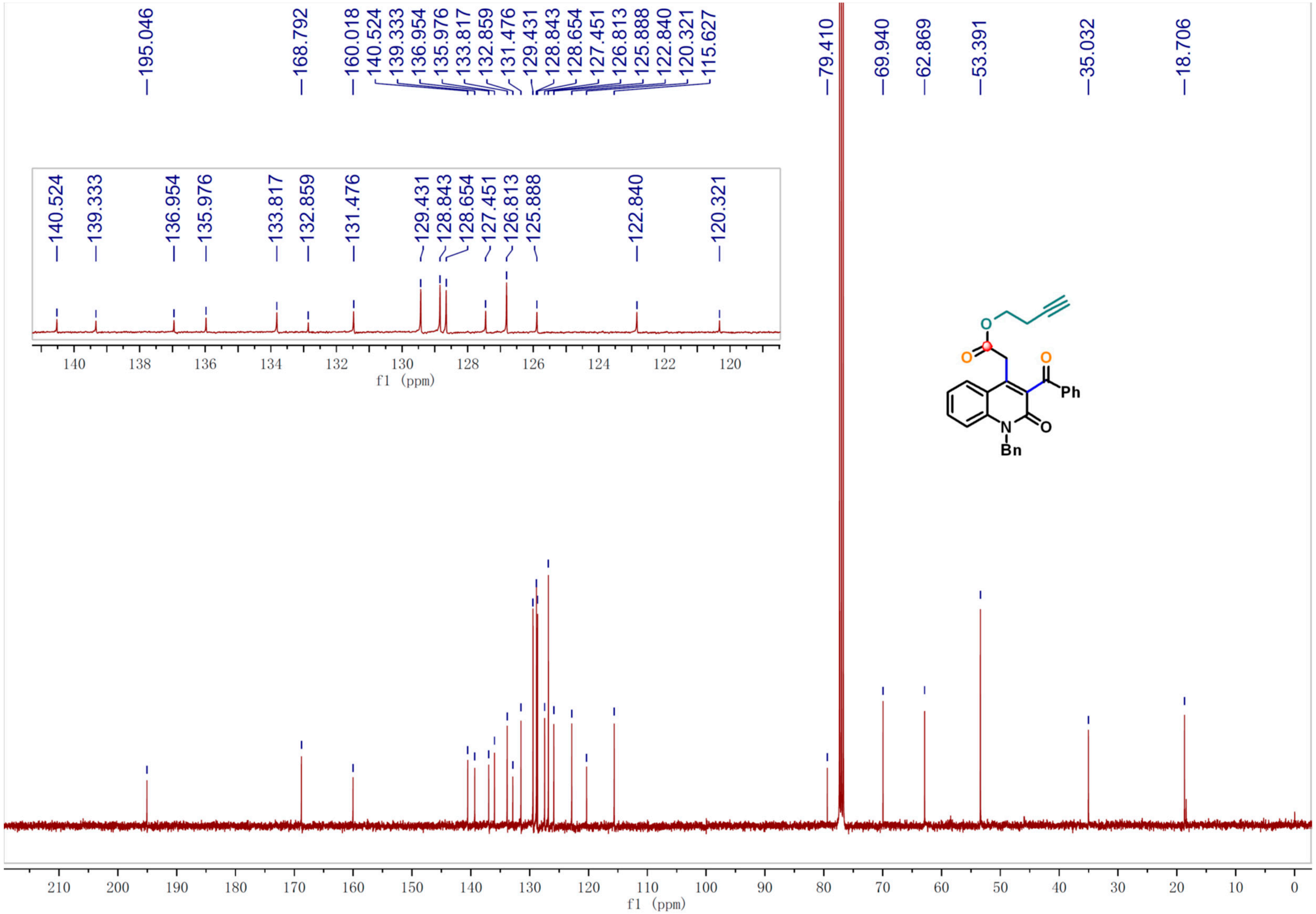

^{13}C NMR Spectrum of Compound 2t

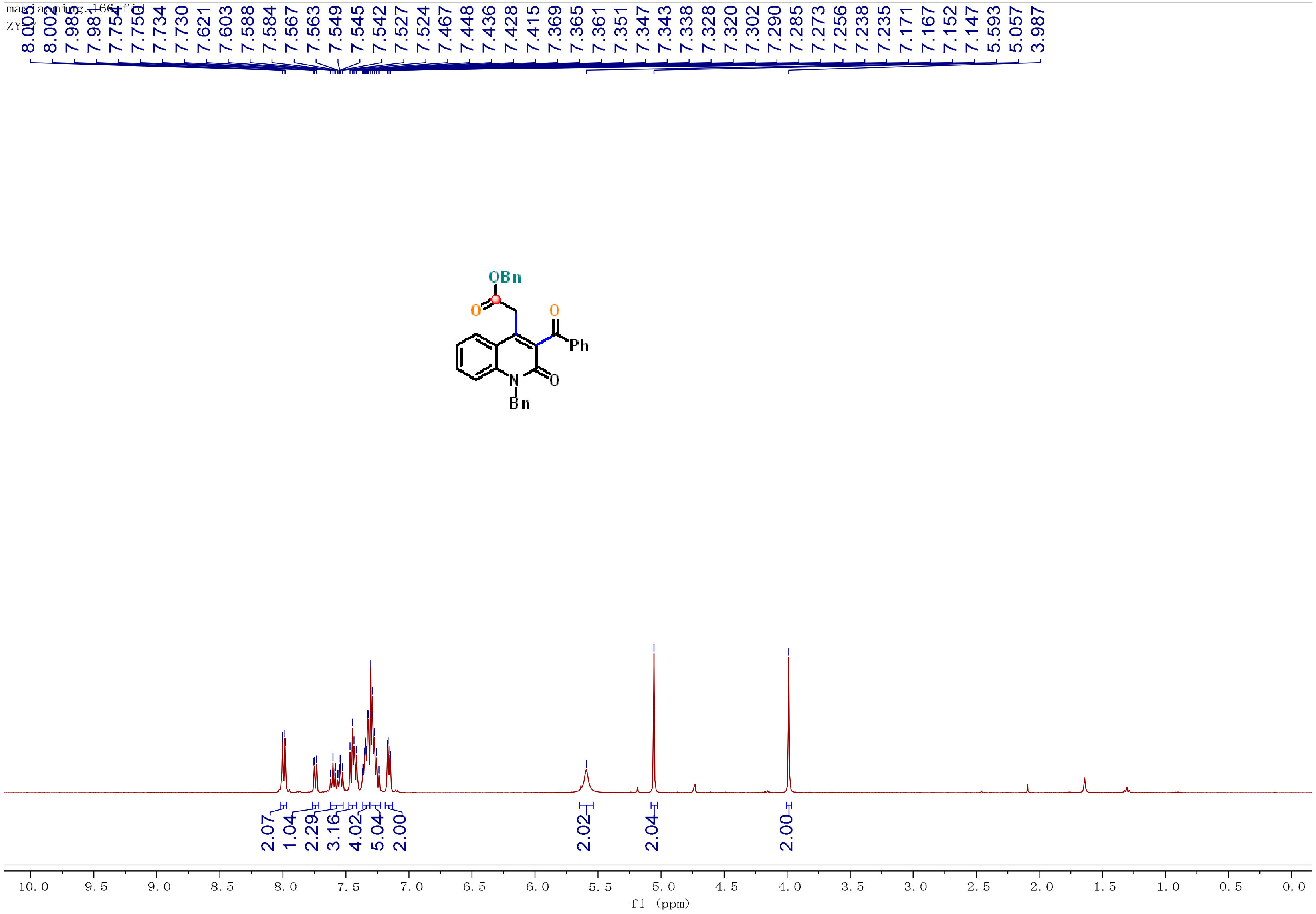

¹H NMR Spectrum of Compound 2u

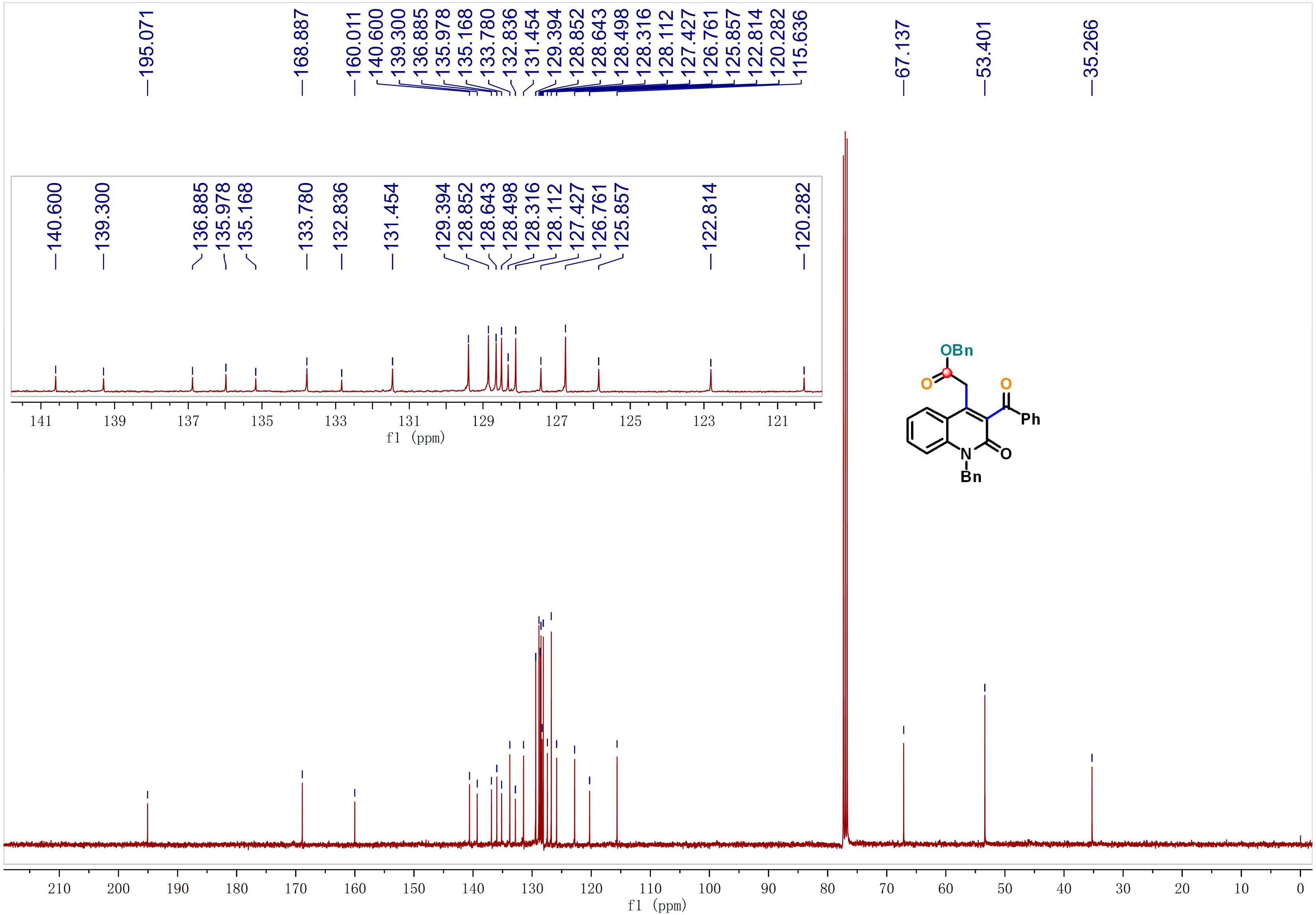

^{13}C NMR Spectrum of Compound 2u

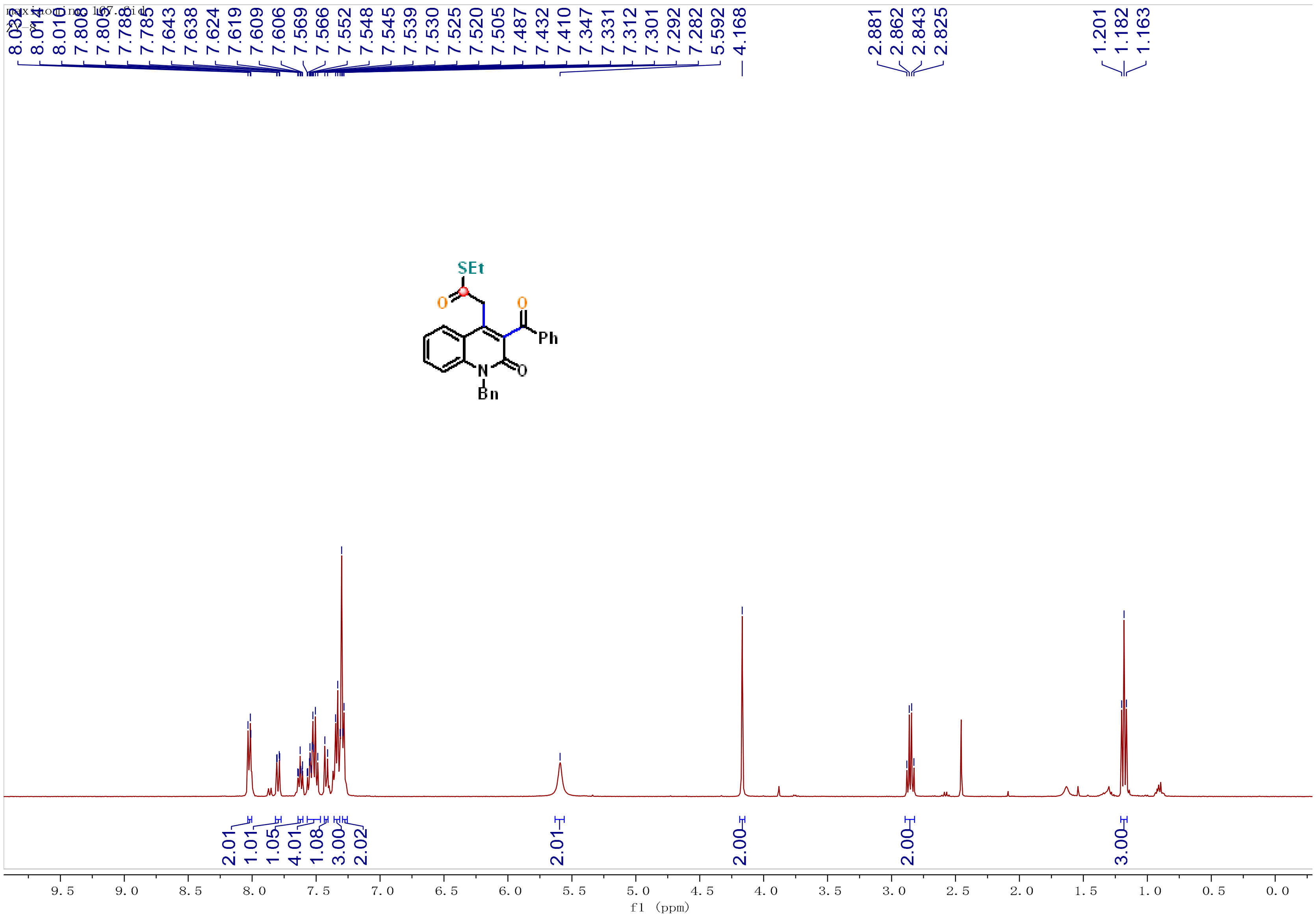

¹H NMR Spectrum of Compound 2v

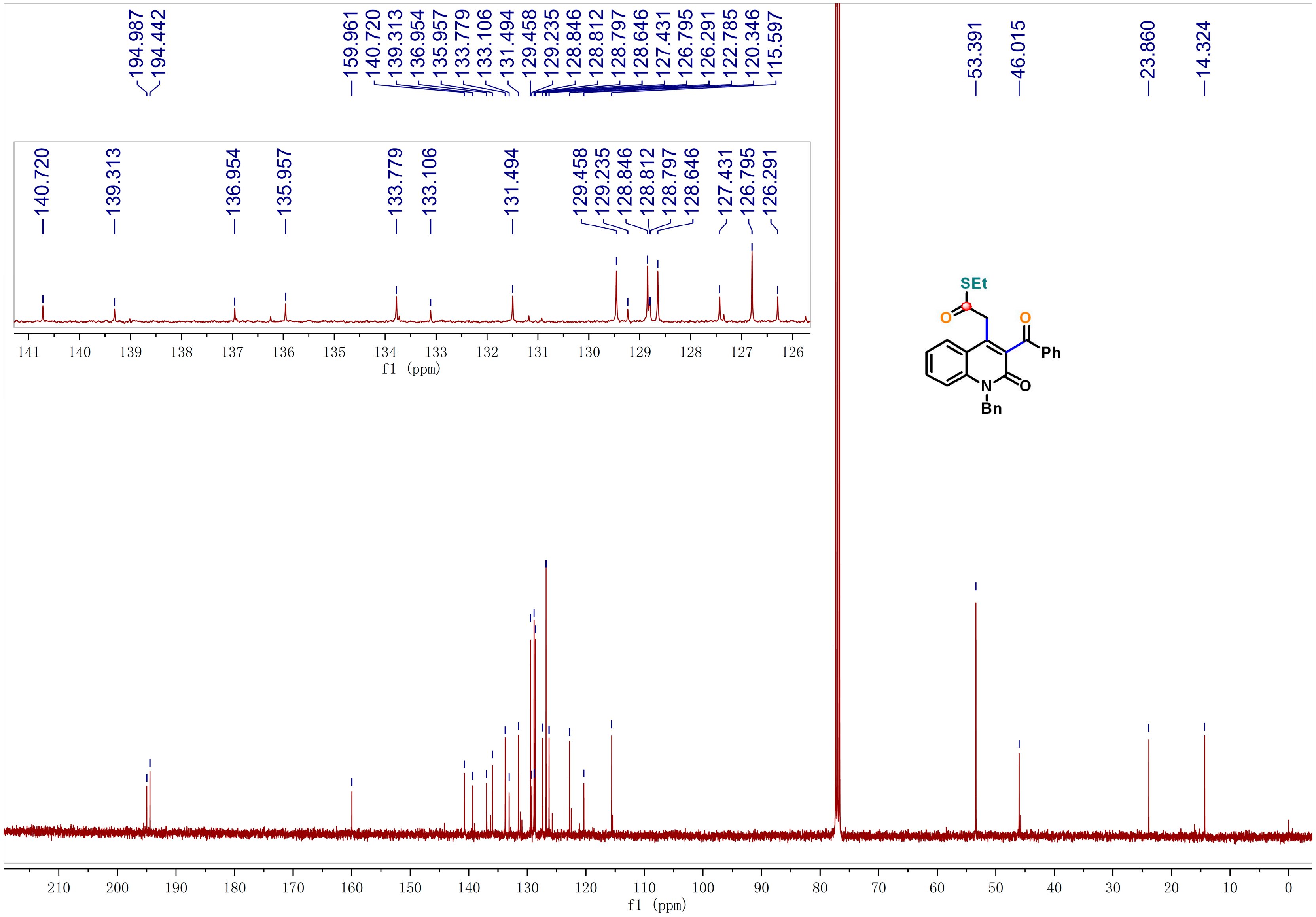

^{13}C NMR Spectrum of Compound 2v

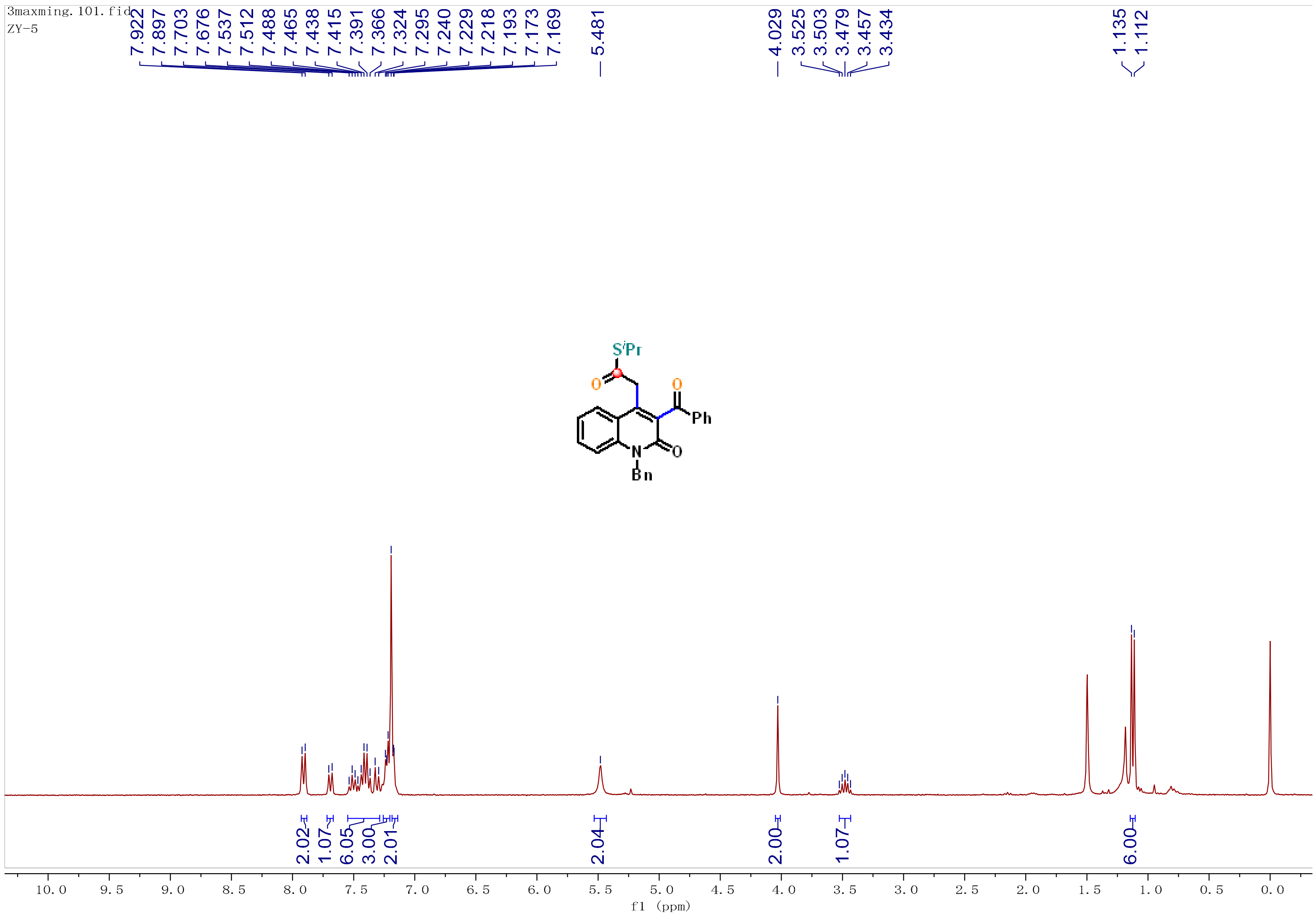

^{1}H NMR Spectrum of Compound 2w

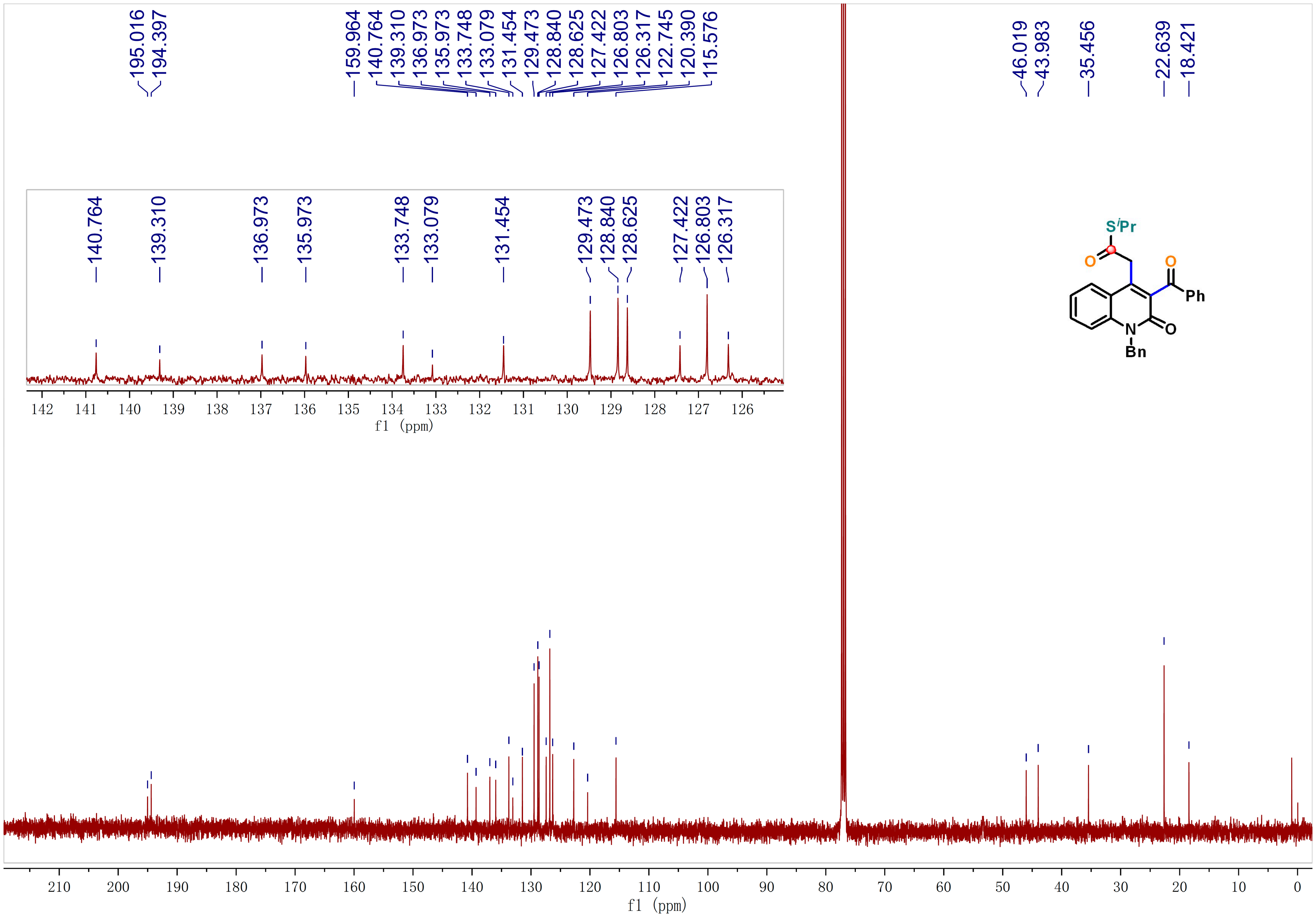

^{13}C NMR Spectrum of Compound 2w

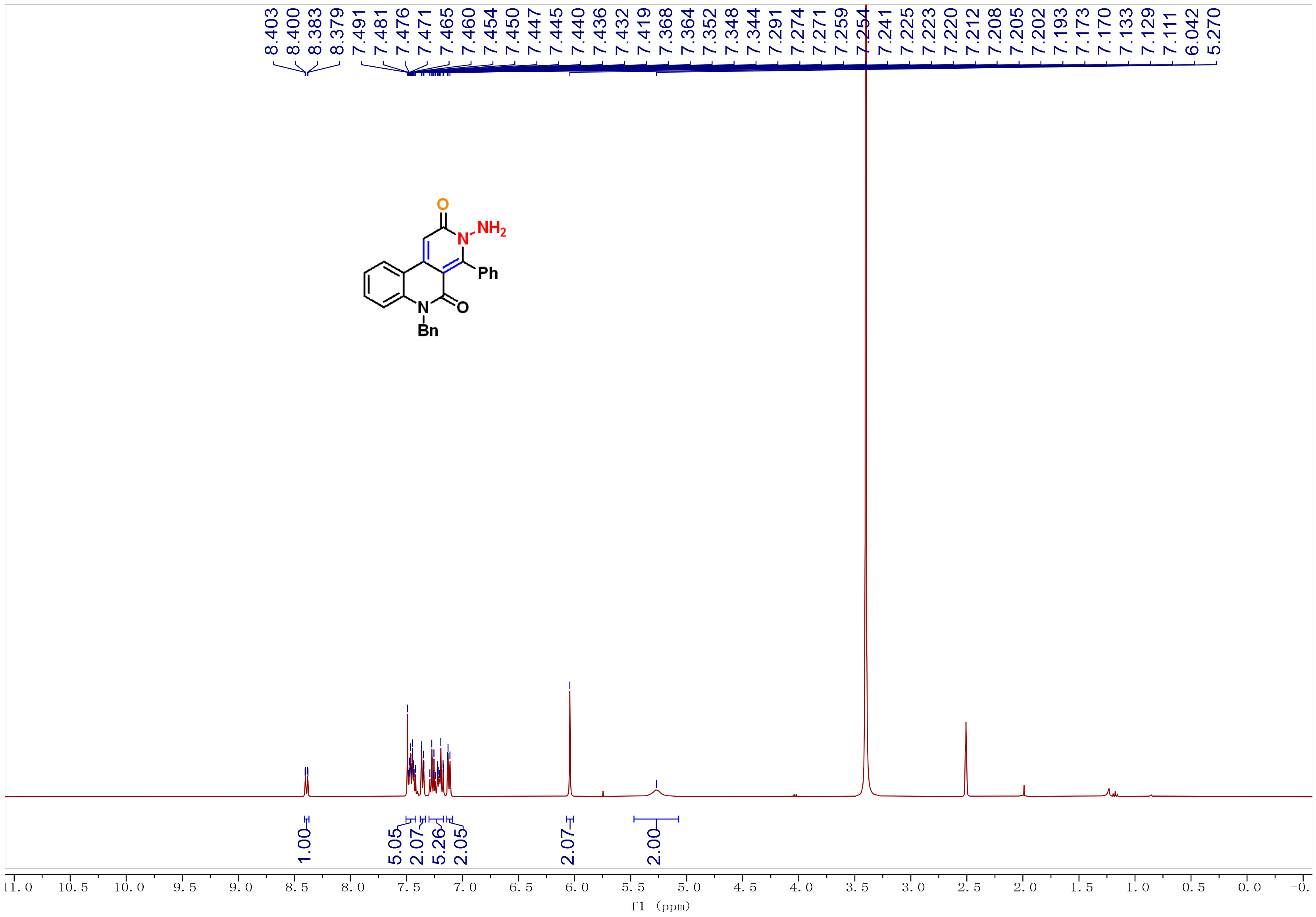

^{1}H NMR Spectrum of Compound 3

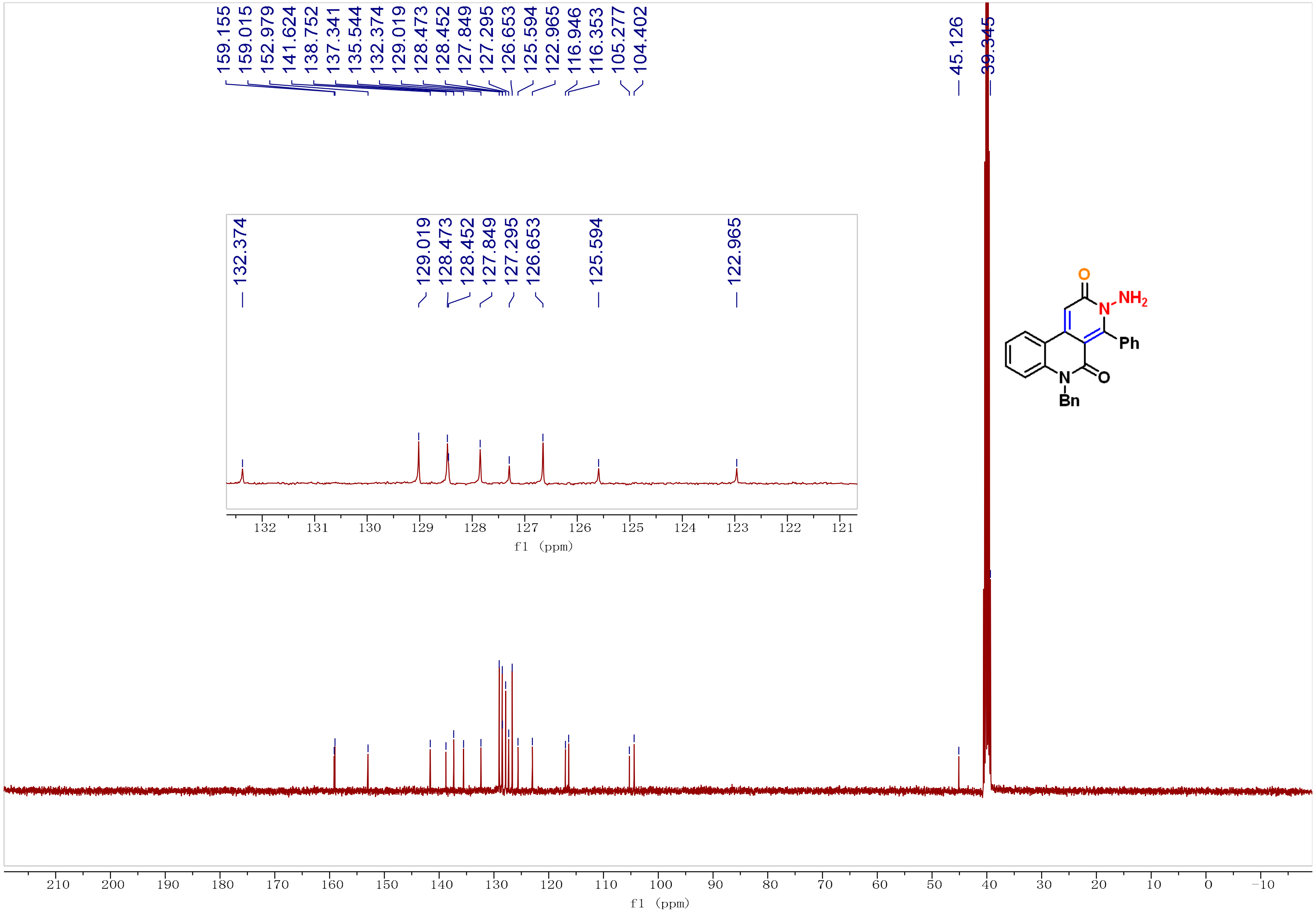

^{13}C NMR Spectrum of Compound 3

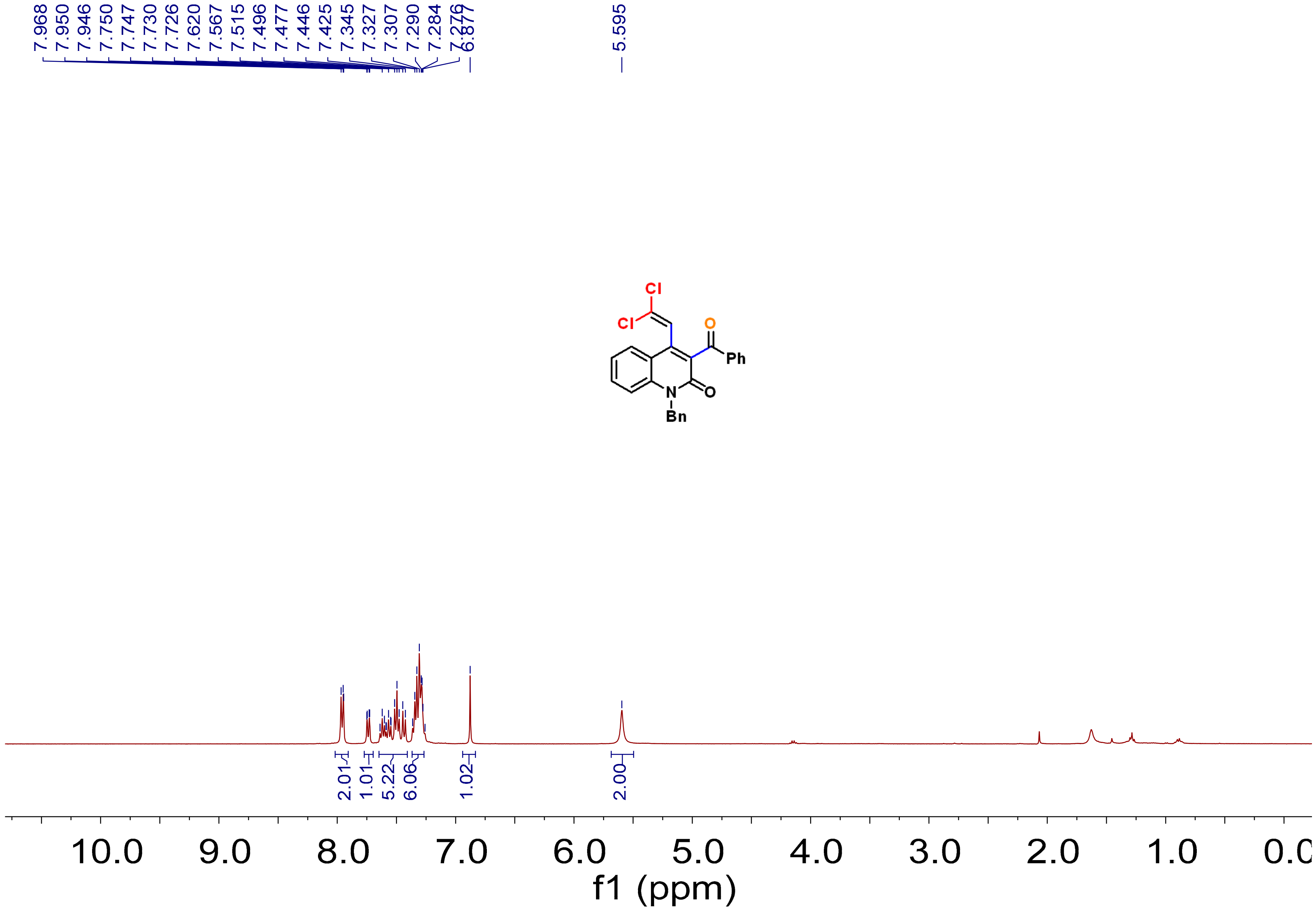

¹H NMR Spectrum of Compound 4

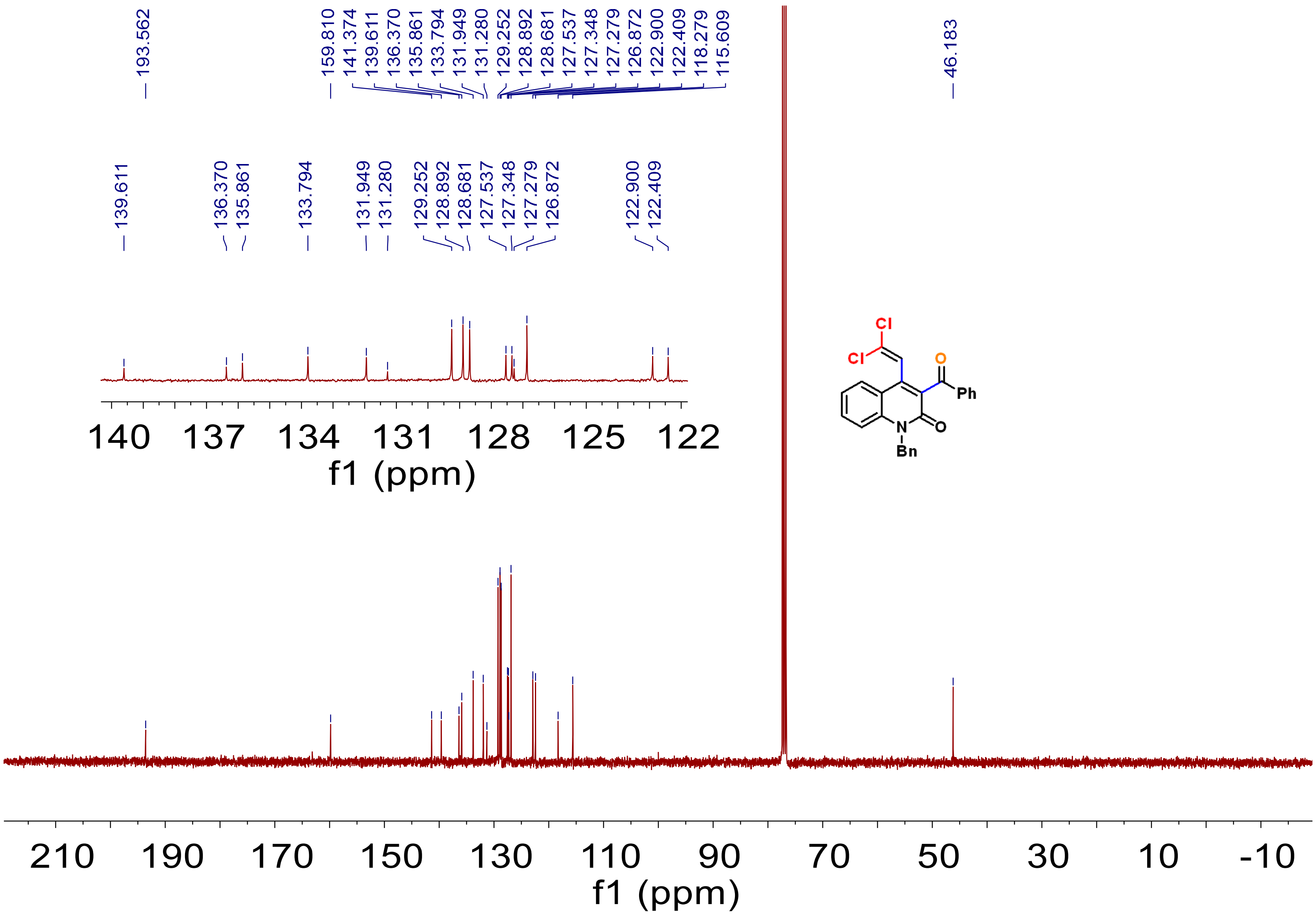

^{13}C NMR Spectrum of Compound 4
